# Global, regional, and national epidemiology of thyroid cancer in middle-aged and elderly adults from 1990 to 2021

**DOI:** 10.3389/fmed.2025.1684535

**Published:** 2025-10-21

**Authors:** Ling Wang, LinZhi Liao, JueZhi Huang, Qi Zhang, YanQing Xiong, FuYu Tian, Xin Liu, YanJia Liu, LuYun Jiang, Yan Xie

**Affiliations:** 1 Clinical Medical College, Chengdu University of Traditional Chinese Medicine, Chengdu, Sichuan, China; 2 Department of Otorhinolaryngology, Hospital of Chengdu University of Traditional Chinese Medicine, Chengdu, China

**Keywords:** thyroid cancer, middle-aged and elderly, global burden of disease, incidence, mortality, disability-adjusted life years

## Abstract

**Background:**

Thyroid cancer (TC) has shown dynamic changes in its global burden over the past decades. This study aimed to evaluate trends in incidence, mortality, and disability-adjusted life years (DALYs) of TC among middle-aged and elderly adults across 204 countries and territories from 1990 to 2021.

**Methods:**

Data were extracted from the Global Burden of Disease Study 2021. Age-standardized rates (ASRs) of incidence, death, and DALYs, estimated annual percentage change (EAPC), and the sociodemographic index (SDI) were used to quantify trends. A Bayesian age-period-cohort (BAPC) model was employed to predict future burden through 2035, and global risk-attributable factors were assessed.

**Results:**

Globally, TC incidence cases among adults aged 55 and older increased by 185% from 1990 to 2021, with deaths and DALYs rising by 116 and 108%, respectively. The age-standardized incidence rate rose significantly (EAPC = 0.95), while mortality and DALY rates slightly declined. Most regions showed rising incidence, with the exception of Central Europe. Females consistently exhibited higher TC burden, but the gender gap is gradually narrowing. Peak incidence occurred at ages 70–74 for females and 85–89 for males in 2021. High body mass index (BMI) emerged as the leading modifiable risk factor.

**Conclusion:**

The rising burden of thyroid cancer in aging populations calls for integrated health policies emphasizing prevention, early detection, and public education. Efforts should focus on reducing modifiable risks, particularly high BMI, and developing age- and sex-specific interventions. Addressing gender disparities and regional inequalities should also be central to global and national cancer control strategies.

## 1 Introduction

Thyroid cancer (TC) is the most common endocrine malignancy, encompassing diverse histological subtypes with distinct frequencies and clinical behaviors. Papillary carcinoma is the predominant and typically indolent form, whereas follicular, medullary, and anaplastic tumors are less common but more aggressive (1). Global cancer statistics for 2022 reported 821,173 new cases of TC and 47,485 deaths, ranking TC as the seventh most common cancer (2). Previous studies have shown that age-standardized death rates (ASDR) and age-standardized years lived with disability (ASYR) are highest among individuals aged 50 to 89 years (3). According to the American Joint Committee on Cancer (AJCC) TNM Staging System, 8th Edition, the age of 55 years serves as a critical prognostic cutoff for differentiated thyroid cancer (DTC) (4). Patients under 55 years old have their disease stage at most limited to stage II, while patients aged 55 and above are subject to the complete I to IV stage classification criteria. This update (changing the previous threshold from 45 years old to 55 years old) reflects the improvement in clinical prognosis and makes 55 years old a widely accepted age cut-off point for distinguishing the clinical risk and disease burden of thyroid cancer. Therefore, we selected the middle-aged and elderly population aged 55 and above as the research subjects.

Based on the most recent GBD 2021 data (5), this study aims to conduct a detailed analysis of global trends in the incidence, mortality, and disability-adjusted life years (DALY) associated with TC among middle-aged and elderly populations. The analysis stratifies data by region, country, age, gender, and Sociodemographic Index (SDI), employing an integrated approach to evaluate the global health burden of TC. As global aging progresses, the disease burden in middle-aged and elderly groups is expected to pose substantial challenges. This research seeks to provide policymakers and public health professionals with practical insights for targeted interventions, offer evidence to inform future TC control efforts, and develop strategies to address the challenges presented by an aging society.

## 2 Methods

### 2.1 Data source and extraction

The GBD 2021 project is a comprehensive, international collaboration aimed at measuring health losses attributed to 371 different diseases and injuries, as well as 88 risk factors, spanning 204 countries and territories from 1990 to 2021 (5, 6). The data required for this study were obtained from the Global Health Data Exchange (GHDx) query tool.^1^

We specifically selected TC data from the GBD, covering 55 years old and above populations in global, 21 GBD regions, 204 countries and regions, and 5 quintiles of the Social Determinants of Health Index (SDI). The data included key indicators such as incidence rates, mortality rates, DALYs, and ASRs. To summarize the age distribution of the burden of TC diseases, we extracted all-age data and age-specific data for the following groups: 55–59, 60–64, 65–69, 70–74, 75–79, 80–84, 85–89, 90–94, 95 + years.

### 2.2 Sociodemographic index

The GBD 2021 study employed a composite metric called the SDI to evaluate the overall sociodemographic status of different locations (5). The SDI assigns a value to each location and year on a scale from 0.0 to 1.0, with 0.0 indicating the least developed sociodemographic conditions and 1.0 indicating the most developed.

### 2.3 Risk factor analysis

The estimates for the burden of TC attributable to risk factor, including attributable deaths and DALYs, were directly obtained from the GBD 2021 results database. This study focusing on evaluating various risk factors linked to an increased incidence of TC among individuals aged 55 and older. Through a pooled analysis, the research aims to assess both the disease burden, as reflected by attributable DALYs, and mortality rates associated with these risk factors.

### 2.4 Bayesian age-period-cohort model for forecasting

In order to project the future burden of TC for individuals aged 55 and above, a BAPC model was applied to forecast age-standardized incidence rates (ASIR), ASDR, ASYR from 2020 to 2035. This model, which accounts for age, period, and cohort factors, provides a detailed approach to understanding future disease trends. Previous research has shown that the BAPC model outperforms other forecasting methods in terms of both coverage and accuracy (7). The BAPC model is expressed as nij = log(λij) = *μ* + αi + βj + γk, where λij represents the number of cases, μ denotes the intercept, and αi, βj, and γk represent the effects of age, period, and cohort, respectively.

### 2.5 Statistical analysis

Due to the differences in population age structure among different countries and regions, it may affect the assessment of TC burden. In this study, ASR was used to control for population differences and was employed as an age-standardization measure. By calculating the ASR indicators of TC incidence, mortality, and DALYs for 204 countries and regions from 1990 to 2021, the burden of TC and its temporal changes were evaluated.

The ASR was calculated using the following equation:


$$\mathrm{ASR}=\frac{\sum_{i=1}^{A}\text{aiwi}}{\sum_{i=1}^{A}\mathrm{wi}}\times 100,000$$

where ai is the age-specific rate in the i -th age group; wi is the number of individuals in the corresponding i -th age group within the standard population; and A is the number of age groups (8). Temporal trends in ASR were analyzed by calculating the estimated annual percentage change (EAPC). The regression equation was first established as follows: Ln (ASR) = *α* + *β*X + *ε* (where α represents the intercept, X is the year, β reflects the linear trend of ASR, and ε is the error term) (9). The rate of change and its 95% CI were subsequently calculated using the following formula: EAPC = 100 × [exp(*β*) − 1]. If both the EAPC and the lower bound of its 95% CI are positive, the ASR is classified as rising. If both the EAPC and the upper bound of its 95% CI are negative, the ASR is considered to be declining. When the 95% CI encompasses zero, the ASR is regarded as stable (10). All data analyses and visualizations presented in this article were conducted using the open-source software R (version 4.4.1) and JD_GBDR (V2.26, Jingding Medical Technology Co., LTD.).

## 3 Results

### 3.1 Global trends

#### 3.1.1 Incidence

From 1990 to 2021, the global number of TC-related incidence increased by 185%, from 42,690 (95% UI: 40508.02 to 45774.31) in 1990 to 121,593 (95% UI: 110,312.35 to 131,189.55) in 2021. The incidence rate of TC rose from 6.36 per 100,000 in 1990 to 8.18 per 100,000 in 2021, with an EAPC of 0.95 (95% CI: 0.84 to 1.06) (Table 1).

**Table 1:** The incident cases and rates for thyroid cancer in middle-aged and elderly patients in 1990/2021 and its temporal trends.

Location	Rate per 100,000 (95% UI)					
	1990		2021		1990–2021	
	Cases	Rate	Cases	Rate	Cases change	EAPC*
Global	42690.39 (40508.02,45774.31)	6.36 (6.03,6.82)	121593.31 (110312.35,131189.55)	8.18 (7.42,8.83)	1.85 (1.64,2.04)	0.95 (0.84,1.06)
Gender						
Male	12233.52 (11641.37,13111.48)	3.93 (3.74,4.21)	41933.05 (36909.83,45964.76)	5.99 (5.28,6.57)	2.43 (2.04,2.75)	1.59 (1.44,1.74)
Female	30456.87 (28466.91,33256.08)	8.46 (7.91,9.24)	79660.26 (70231.07,89854.97)	10.13 (8.93,11.43)	1.61 (1.39,1.86)	0.68 (0.58,0.78)
SDI level						
High	19860.43 (18895.52,20586.95)	10.65 (10.13,11.04)	43875.27 (40234.13,46271.20)	12.72 (11.66,13.41)	1.21 (1.09,1.31)	0.82 (0.59,1.05)
High-middle	12116.08 (11326.32,12791.82)	7.02 (6.57,7.41)	28901.32 (25722.45,32129.83)	8.34 (7.42,9.27)	1.39 (1.16,1.68)	0.63 (0.52,0.75)
Middle	6584.16 (5965.80,7908.10)	3.79 (3.44,4.56)	32829.44 (28136.04,36916.07)	6.99 (5.99,7.86)	3.99 (3.28,4.64)	2.03 (1.95,2.11)
Low-middle	2888.42 (2512.55,3570.63)	2.87 (2.49,3.54)	12281.79 (10614.44,13884.95)	5.09 (4.40,5.76)	3.25 (2.59,3.88)	1.89 (1.86,1.93)
Low	1179.57 (973.84,1474.43)	3.16 (2.61,3.95)	3598.07 (2883.71,4481.26)	4.38 (3.51,5.46)	2.05 (1.49,2.76)	1.05 (0.94,1.15)
GBD Regions						
Andean Latin America	197.98 (161.72,235.72)	5.90 (4.82,7.02)	1236.25 (938.80,1573.09)	12.48 (9.48,15.88)	5.24 (3.83,7.19)	2.48 (2.35,2.62)
Australasia	300.02 (268.65,339.78)	7.62 (6.82,8.62)	1133.78 (932.72,1350.20)	12.83 (10.56,15.28)	2.78 (2.02,3.68)	2.52 (2.09,2.95)
Caribbean	194.05 (179.28,211.56)	4.50 (4.16,4.91)	645.72 (560.24,737.95)	6.97 (6.05,7.97)	2.33 (1.85,2.89)	1.60 (1.39,1.82)
Central Asia	377.05 (344.93,415.73)	4.71 (4.31,5.20)	784.52 (693.27,877.98)	5.39 (4.76,6.03)	1.08 (0.79,1.40)	0.42 (−0.28,1.12)
Central Europe	2507.30 (2374.28,2650.69)	9.45 (8.95,9.99)	3062.26 (2760.35,3340.88)	8.27 (7.45,9.02)	0.22 (0.11,0.35)	−0.51 (−0.73, −0.28)
Central Latin America	773.48 (740.29,807.27)	5.70 (5.46,5.95)	4029.18 (3570.88,4483.74)	9.42 (8.35,10.48)	4.21 (3.63,4.86)	1.43 (1.30,1.57)
Central Sub-Saharan Africa	75.54 (54.07,113.43)	2.01 (1.44,3.02)	211.56 (134.75,332.41)	2.34 (1.49,3.68)	1.80 (1.04,2.68)	0.46 (0.31,0.60)
East Asia	5287.71 (4457.38,6149.04)	3.55 (2.99,4.13)	24471.56 (19717.43,30165.00)	6.24 (5.03,7.69)	3.63 (2.69,5.02)	2.04 (1.89,2.20)
Eastern Europe	3229.67 (3042.50,3460.10)	6.61 (6.22,7.08)	5488.91 (4950.87,6100.19)	8.84 (7.98,9.83)	0.70 (0.50,0.90)	1.05 (0.75,1.35)
Eastern Sub-Saharan Africa	632.34 (517.43,768.46)	5.20 (4.25,6.32)	1682.35 (1240.85,2267.13)	6.22 (4.59,8.39)	1.66 (1.04,2.49)	0.47 (0.34,0.60)
High-income Asia Pacific	3847.21 (3558.24,4220.34)	11.00 (10.18,12.07)	9442.42 (8082.48,10727.31)	13.39 (11.46,15.22)	1.45 (1.19,1.74)	0.91 (0.57,1.26)
High-income North America	6766.46 (6393.53,7004.48)	11.68 (11.04,12.09)	18044.49 (16897.79,18914.66)	16.03 (15.02,16.81)	1.67 (1.55,1.77)	1.07 (0.87,1.28)
North Africa and Middle East	1194.09 (972.67,1665.35)	4.22 (3.44,5.89)	7018.93 (5886.17,8223.09)	9.21 (7.72,10.79)	4.88 (3.37,6.42)	2.82 (2.65,3.00)
Oceania	17.90 (12.66,24.20)	3.72 (2.63,5.03)	53.27 (34.16,73.45)	4.32 (2.77,5.95)	1.98 (1.37,2.87)	0.40 (0.32,0.47)
South Asia	2462.19 (2047.97,3113.29)	2.59 (2.16,3.28)	12716.27 (10419.20,14583.26)	5.12 (4.20,5.87)	4.16 (3.08,5.22)	2.26 (2.15,2.37)
Southeast Asia	2515.12 (2107.85,3038.02)	5.94 (4.98,7.18)	11515.33 (9416.83,13365.36)	10.05 (8.22,11.67)	3.58 (2.90,4.36)	1.67 (1.59,1.76)
Southern Latin America	529.45 (474.96,589.11)	6.68 (6.00,7.44)	1095.19 (959.09,1257.42)	7.44 (6.52,8.54)	1.07 (0.79,1.45)	0.42 (0.21,0.64)
Southern Sub-Saharan Africa	124.42 (99.06,156.43)	2.81 (2.24,3.54)	430.27 (344.82,487.82)	4.42 (3.54,5.01)	2.46 (1.73,3.17)	1.57 (1.35,1.79)
Tropical Latin America	616.92 (577.51,652.26)	4.07 (3.81,4.31)	2208.50 (2024.77,2352.08)	4.99 (4.57,5.31)	2.58 (2.31,2.85)	0.46 (0.32,0.59)
Western Europe	10954.10 (10308.05,11600.34)	11.28 (10.61,11.95)	16112.71 (14563.22,17563.89)	10.80 (9.77,11.78)	0.47 (0.34,0.60)	0.20 (−0.06,0.46)
Western Sub-Saharan Africa	87.41 (64.50,104.17)	0.61 (0.45,0.72)	209.81 (168.23,262.89)	0.65 (0.52,0.82)	1.40 (0.91,2.11)	0.11 (0.04,0.18)

EAPC, estimated annual percentage change; SDI, Sociodemographic Index; UI, uncertainty interval. *EAPC is expressed as 95% Cis.

#### 3.1.2 Mortality

Over the past three decades, global TC-associated deaths rose by 116%, from 16,891 (95% UI: 15,743.67 to 18,564.92) in 1990 to 36,442 (95% UI: 32,220.59 to 39,277.83) in 2021. The TC-related death rate, however, decreased slightly, from 2.52 per 100,000 in 1990 to 2.45 per 100,000 in 2021, showing a negative EAPC of −0.06 (95% CI: −0.08 to −0.03) (Supplementary Table S1).

#### 3.1.3 DALYs

From 1990 to 2021, the global number of TC-related DALYs increased by 108%, from 375,068 (95% UI: 351,621.47 to 412,721.98) in 1990 to 779,088 (95% UI: 695,795.16 to 843,551.32) in 2021. Despite this, the DALY rate decreased from 55.86 per 100,000 in 1990 to 52.43 per 100,000 in 2021, with an EAPC of −0.23 (95% CI: −0.26 to −0.21; Supplementary Table S2).

### 3.2 SDI regional trends

#### 3.2.1 Incidence

**Figure 1:**
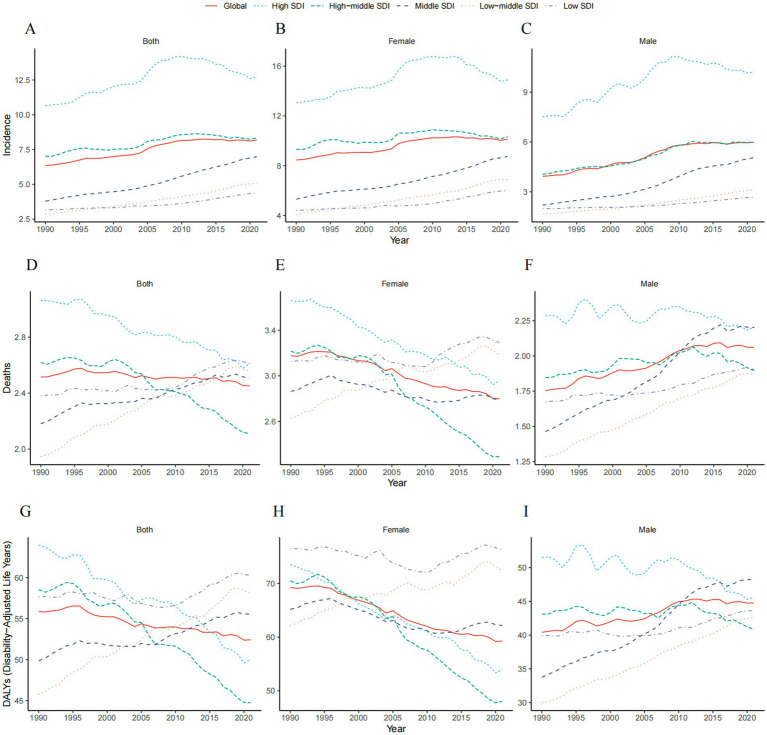
Global trends by SDI quintiles for thyroid cancer in middle-aged and elderly patients from 1990 to 2021. **(A)** Incidence rate of female and male. **(B)** Incidence rate of female. **(C)** Incidence rate of male. **(D)** Deaths rate of female and male. **(E)** Deaths rate of female. **(F)** Deaths rate of male. **(G)** DALYs rate of female and male. **(H)** DALYs rate of female. **(I)** DALYs rate of male.

In 2021, high SDI regions saw the highest number of TC cases (43,875; 95% UI: 40,234.13 to 40,234.13), while low SDI regions reported the lowest (3,598; 95% UI: 2,883.71 to 4,481.26). The incidence rate of TC increased across all SDI regions, with the highest in high SDI (12.72; 95% CI: 11.66 to 13.41) and the greatest rise in middle SDI regions (EAPC: 2.03; 95% CI: 1.95 to 2.11; Table 1; Figures 1A–C).

#### 3.2.2 Mortality

TC-associated mortality increased across all SDI regions, with the most significant rise in low-middle SDI areas (214%). In 2021, the middle SDI region recorded the highest number of TC deaths (11,789; 95% UI: 10,098.38 to 13,014.63), while the low SDI region had the lowest (2,143; 95% UI: 1,724.01 to 2,629.53). Mortality rates were highest in the low SDI region (2.61; 95% UI: 2.10 to 3.20) and lowest in the high-middle SDI region (2.11; 95% UI: 1.88 to 2.32). The highest EAPC in mortality rates was in low-middle SDI regions (0.90; 95% UI: 0.86 to 0.94), while the lowest was in high SDI regions (−0.49; 95% UI: −0.52 to −0.45; Supplementary Table S1; Figures 1D–F).

#### 3.2.3 DALYs

The middle SDI region had the largest number of TC-related DALYs in 2021 (261,020; 95% UI: 222,137.03 to 287,484.24), while the low SDI region had the fewest (49,466; 95% UI: 39,783.96 to 61,160.91). DALY rates were highest in low SDI regions (60.28; 95% UI: 48.48 to 74.53) and lowest in high-middle SDI regions (44.75; 95% UI: 40.08 to 49.54). The greatest EAPC for DALY rates was in low-middle SDI regions (0.78; 95% UI: 0.74 to 0.82), while the lowest was in high-middle SDI regions (−0.95; 95% UI: −1.03 to −0.87; Supplementary Table S2; Figures 1G–I).

### 3.3 Regional trends

#### 3.3.1 Incidence

**Figure 2:**
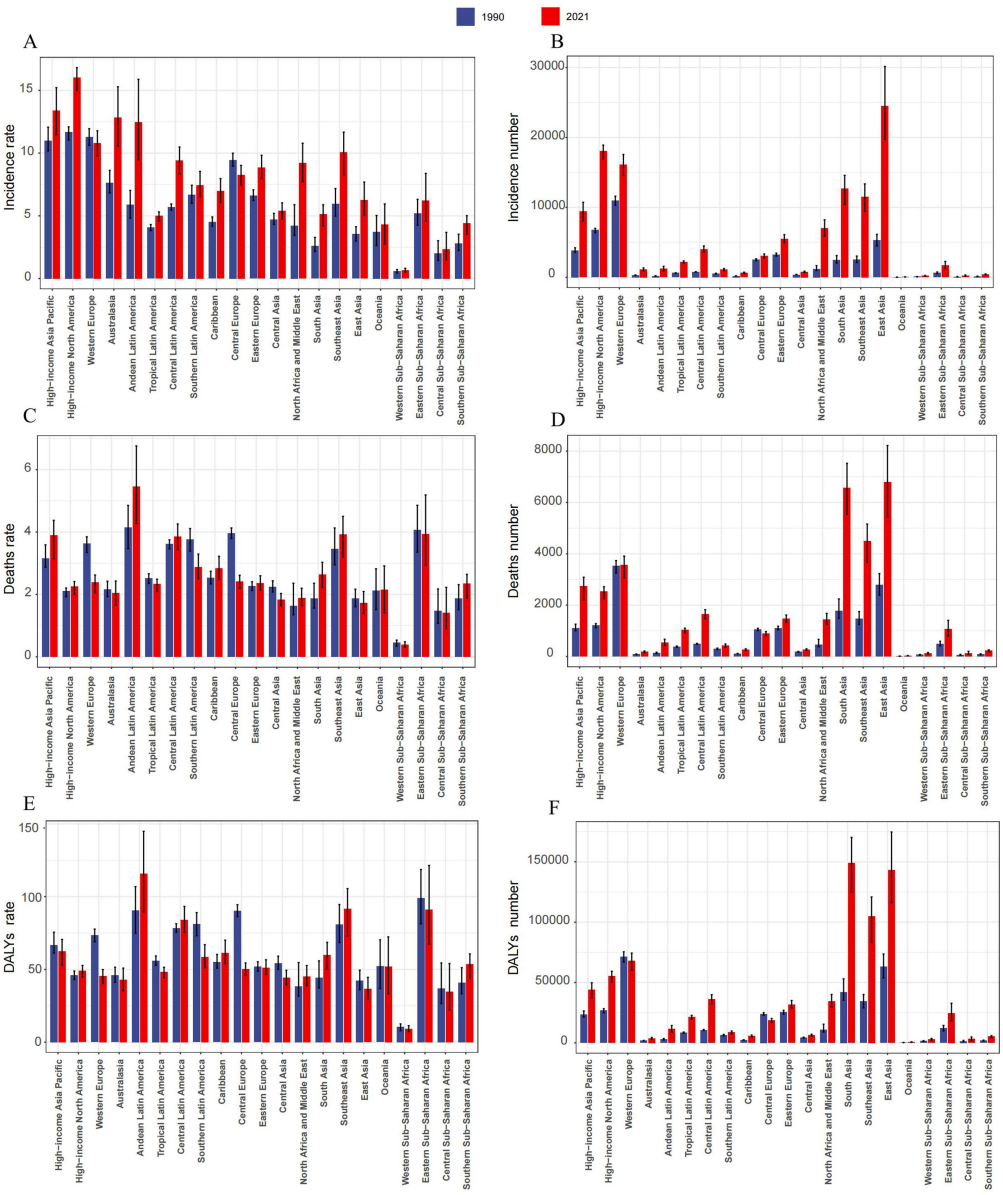
The number of cases and rates for thyroid cancer in middle-aged and elderly patients across 21 Global Burden of Disease regions from 1990 and 2021. **(A)** Incidence rate. **(B)** Incidence number. **(C)** Deaths rate. **(D)** Deaths number. **(E)** DALYs rate. **(F)** DALYs number.

**Figure 3:**
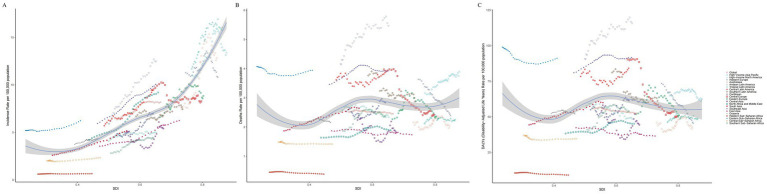
Incidence **(A)**, Deaths **(B)**, and DALYs **(C)** Rates for thyroid cancer in middle-aged and elderly patients for 21 Global Burden of Disease regions from 1990 to 2021 by SDI.

In 2021, The largest incident cases of TC were in East Asia (24,472, 95% UI, 19717.43 to 30165.00), High-income North America (18,044, 95% UI, 16897.79 to 18914.66) and Western Europe (16,113, 95% UI, 14563.22 to 17563.89). The lowest incidence numbers were in Oceania (53, 95% UI, 34.16 to 73.45), Western Sub-Saharan Africa (210, 95% UI, 168.23 to 262.89) and Central Sub-Saharan Africa (212, 95% UI, 134.75 to 332.41). High-income North America (16.03, 95% UI, 15.02 to 16.81), High-income Asia Pacific (13.39, 95% UI, 11.46 to 15.22), and Australasia (12.83, 95% UI, 10.56 to 15.28) had the highest incidence rates in 2021, whereas Western Sub-Saharan Africa (0.65, 95% UI, 0.52 to 0.82), Central Sub-Saharan Africa (2.34, 95% UI, 1.49 to 3.68), and Oceania (4.32, 95% UI, 2.77 to 5.95) had the lowest incidence rates. From 1990 to 2021, the incidence rate of TC increased in all regions except Central Europe (EAPC = -0.51, 95% CI -0.73 to −0.28). The largest increase in the incidence rate was in North Africa and Middle East (EAPC = 2.82, 95% CI 2.65 to 3.00), followed by Australasia (EAPC = 2.52, 95% CI 2.09 to 2.95) and Andean Latin America (EAPC = 2.48, 95% CI 2.35 to 2.62; Table 1; Figures 2A,B). The global SDI was 0.67 in 2021; 10 regions had higher incidences of TC than the global mean, whereas 11 regions had lower incidences than the global mean (Figure 3A).

#### 3.3.2 Mortality

In 2021, The highest numbers of TC deaths were in East Asia (6,797, 95% UI, 5437.46 to 8217.29), South Asia (6,565, 5541.37 to 7524.38) and Southeast Asia (4500.03, 3667.85 to 5158.09). The lowest death numbers were in Oceania (27, 17.43 to 35.98), Western Sub-Saharan Africa (124, 100.47 to 154.63), and Central Sub-Saharan Africa (128, 81.72 to 200.9). Andean Latin America (5.47, 4.27 to 6.76), Eastern Sub-Saharan Africa (3.94, 2.93 t to 5.19), and Southeast Asia (3.93, 3.20 to 4.50) had the highest death rates in 2021, whereas these rates were lowest in Western Sub-Saharan Africa (0.39, 0.31 to 0.48), Central Sub-Saharan Africa (1.41, 0.91 to 2.23), and East Asia (1.73, 1.39 to 2.10). From 1990 to 2021, the largest decrease in the death rate was in Central Europe (EAPC = -1.45, 95% CI -1.66 to −1.24), followed by Western Europe (EAPC = -1.15, 95% CI -1.20 to −1.10) and Central Asia (EAPC = -0.74, 95% CI -1.02 to −0.46). The largest increase in the death rate was in South Asia (EAPC = 1.09, 95% CI 1.04 to 1.14), followed by Andean Latin America (EAPC = 0.89, 95% CI 0.80 to 0.97) and Southern Sub-Saharan Africa (EAPC = 0.87, 95% CI 0.68 to 1.06) (Supplementary Table S2; Figures 2C,D). In 2021, 8 regions had rates of deaths that were higher than the global mean, whereas 13 regions had rates that were lower than the global mean (Figure 3B).

#### 3.3.3 DALYs

In 2021, The highest number of DALYs due to TC were in South Asia (148,932, 95% UI, 124787.50 to 170245.54), East Asia (143,248, 115994.95 to 174708.65) and Southeast Asia (105,000, 83334.61 to 120870.25). Meanwhile, the lowest numbers were in Oceania (641, 410.15 to 893.03), Western Sub-Saharan Africa (2,921, 2340.88 to 3645.36), and Central Sub-Saharan Africa (3,126, 1992.23 to 4875.52). Andean Latin America (115.81, 89.5 to 144.89), Southeast Asia (91.66, 72.75 to 105.51), and Eastern Sub-Saharan Africa (91.15, 67.5 to 121.42) had the highest DALY rates in 2021, whereas Western Sub-Saharan Africa (9.09, 7.28 to 11.34), Central Sub-Saharan Africa (34.65, 22.08 to 54.03), and East Asia (36.53, 29.58 to 44.55) had the lowest DALY rates. From 1990 to 2021, the largest decrease in the DALYs rate was in Central Europe (EAPC = -2.15, 95% CI -2.46 to −1.85), followed by Western Europe (EAPC = -1.39, 95% CI -1.48 to −1.31) and Southern Latin America (EAPC = -0.98, 95% CI -1.24 to −0.73). The largest increase in the DALYs rate was in South Asia (EAPC = 1.00, 95% CI 0.95 to 1.05), followed by Southern Sub-Saharan Africa (EAPC = 0.98, 95% CI 0.67 to 1.28) and Andean Latin America (EAPC = 0.77, 95% CI 0.66 to 0.88; Supplementary Table S3; Figures 2E,F). In 2021, 9 regions had rates of DALYs that were higher than the global mean, whereas 12 regions had rates that were lower than the global mean (Figure 3C).

### 3.4 National trends

#### 3.4.1 Incidence

**Figure 4:**
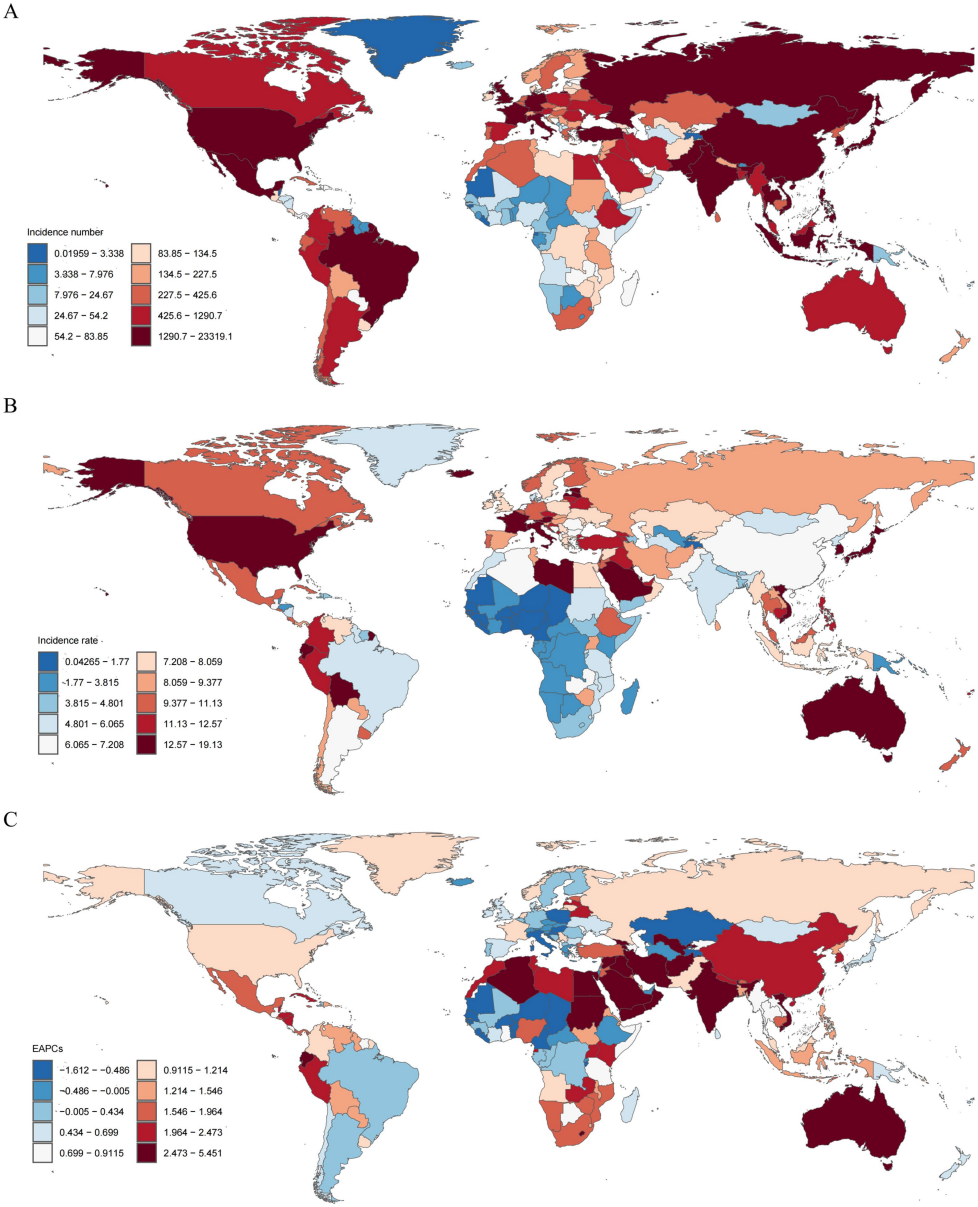
The incidence for thyroid cancer in middle-aged and elderly patients in 204 countries and territories from 1990 to 2021. **(A)** The number of incidence cases. **(B)** Disease burden of incidence rate. **(C)** EAPC for incidence rate.

In 2021, China had the highest TC incidence (23,319, 95% UI, 18653.94 to 28913.04), followed by United States of America (16,884, 15780.67 to 17632.52) and India (10,306, 11871.10 to 8479.27). The highest incidence rates were found in Qatar (19.13, 12.93 to 27.04), Bahrain (18.50, 12.68 to 25.03), and Iceland (18.35, 15.24 to 22.39). The lowest incidence rates were found in Tajikistan (0.04, 0.03 to 0.07), Kiribati (0.16, 0.10 to 0.32), and Nigeria (0.21, 0.14 to 0.39) (Figure 4). The global incidence rate of TC in 2021 was 8.18 (95% UI, 7.42–8.43); the incidence rate in 96 countries were higher than the global average, while that in 108 countries were lower than the global average (Supplementary Figure S1A; Supplementary Tables S3, S6).

#### 3.4.2 Mortality

In 2021, China also had the most cases of death cases (6,489; 95% UI, 5154.56 to 7903.48), followed by India (5,203, 4379.31 to 5947.24) and United States of America (2,286, 2026.96 to 2429.72). The highest death rates were found in Bolivia (Plurinational State of) (7.45, 4.63 to 10.27), Ethiopia (6.77, 4.69 to 9.86), and Ecuador (6.29, 4.95 to 7.75). At the same time, the lowest death rates were found in Tajikistan (0.02, 0.01 to 0.03), Kiribati (0.10, 0.07 to 0.20), and Nigeria (0.11, 0.08 to 0.21; Supplementary Figure S2). The global deaths rate of TC in 2021 was 2.45 (95% UI, 2.17–2.64); the deaths rate in 98 countries were higher than the global average, while that in 106 countries were lower than the global average (Supplementary Figure S1B; Supplementary Tables S4, S7).

#### 3.4.3 DALYs

In 2021, China had the highest number of DALYs (136,672, 95% UI, 109610.49 to 167935.16), followed by India (118,082, 98959.73 to 135808.10) and United States of America (50,212, 45930.29 to 54156.97). The highest estimated DALY rates were observed in Bolivia (Plurinational State of) (163.77, 102.50 to 225.91), Ethiopia (150.92, 102.14 to 225.71), and Cambodia (136.56, 85.05 to 191.99). On the contrary, Tajikistan (0.46, 0.30 to 0.66), Kiribati (2.40, 1.58 to 4.77), and Nigeria (2.65, 1.77 to 4.98) had the lowest DALY rates in 2021 (Supplementary Figure S3). The global deaths rate of TC in 2021 was 52.43 (95% UI, 46.82 to 56.77); the DALYs rate in 101 countries were higher than the global average, while that in 103 countries were lower than the global average (Supplementary Figure S1C; Supplementary Tables S5, S8).

### 3.5 Age and sex trends

Between 1990 and 2021, the global burden of TC showed an increasing trend in both females and males, with females consistently experiencing a significantly higher burden than males. In 2021, the ASIR for TC in women was approximately 1.69 times higher than in men, with women having an incidence rate of 10.13 per 100,000 compared to 5.99 per 100,000 in men. Similarly, the ASDR was 1.36 times higher in females (2.80 per 100,000) than in males (2.06 per 100,000). Furthermore, the ASYR for TC in females was 1.32 times higher, at 59.24 per 100,000, compared to 44.77 per 100,000 in males. These findings highlight significant gender disparities in TC burden (Table 1; Supplementary Figure S4).

Over the past three decades, the gender gap in TC burden has narrowed, primarily due to a faster decrease in rates among females and a rise in rates among males. The ASIR increased for both genders, with an EAPC of 1.59 (95% CI: 1.44 to 1.74) for males and 0.68 (95% CI: 0.58 to 0.78) for females. Notably, the ASDR (EAPC: -0.37, 95% CI: −0.41 to −0.33) and ASYR (EAPC: -0.59, 95% CI: −0.62 to −0.55) for females both showed a declining trend, while for males, both ASDR (EAPC: 0.57, 95% CI: 0.53 to 0.61) and ASYR (EAPC: 0.40, 95% CI: 0.34 to 0.45) increased.

**Figure 5:**
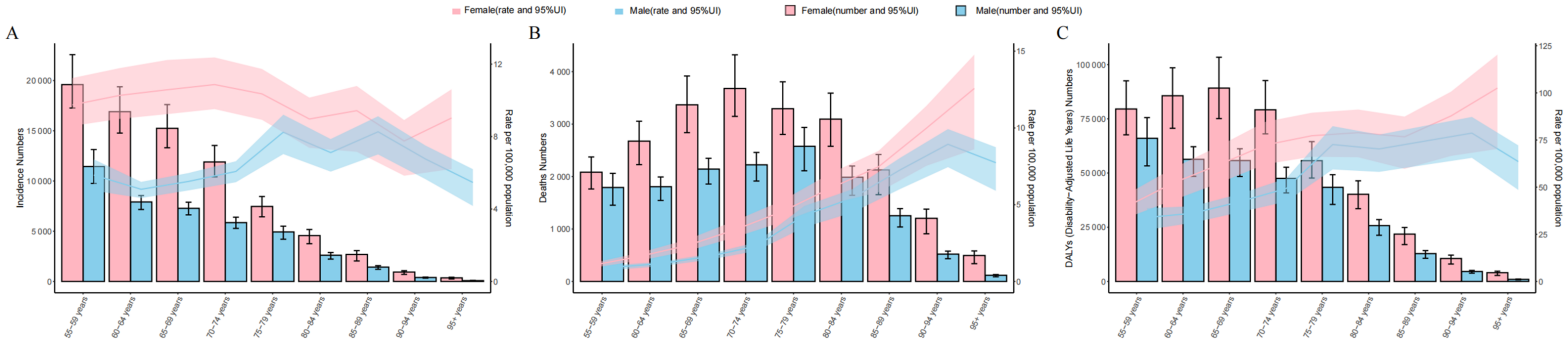
Global counts and incidence. **(A)** Deaths **(B)** and DALYs **(C)** Rates of thyroid cancer in the middle-aged and elderly patients by age and sex, 2021. Error bars indicate the 95% uncertainty intervals (95% UI) for incidence **(A)**, Death **(B)** and DALYs **(C)**. Shading indicates the upper and lower limits of the 95% UI.

In 2021, the highest number of incident cases of TC for both females and males occurred in the 55–59 age group, followed by a gradual decline with advancing age. The ASIR for females was consistently higher than for males across all age groups. The peak ASIR in females was observed in the 70–74 age group, whereas in males, it peaked in the 85–89 age group (Figure 5A). The peak number of deaths from TC in females occurred in the 70–74 age group, while in males, it was in the 75–79 age group. The ASDR increased with age in both sexes, reaching a peak in males at ages 90–94 before gradually declining (Figure 5B). The highest number of DALYs attributed to TC in females was observed in the 65–69 age group, whereas in males, it peaked in the 55–59 age group. The ASYR increased with age in both sexes, peaking in males at ages 90–94 and then showing a gradual decline (Figure 5C). Overall, the largest numbers of incident cases, deaths, and DALYs were recorded in the 55–59, 70–74, and 65–69 age groups, respectively. Consequently, the burden of TC is predominantly concentrated within the 55–74 age subgroup (Supplementary Table S9).

### 3.6 Risk factors

The risk factors for TC align with findings from previous studies. Our analysis identified high BMI as a significant risk factor. This factor showed the strongest association with TC risk in Andean Latin America and the weakest in Western Sub-Saharan Africa, both in terms of mortality and DALYs (Supplementary Figure S5).

### 3.7 Predicted trends

**Figure 6:**
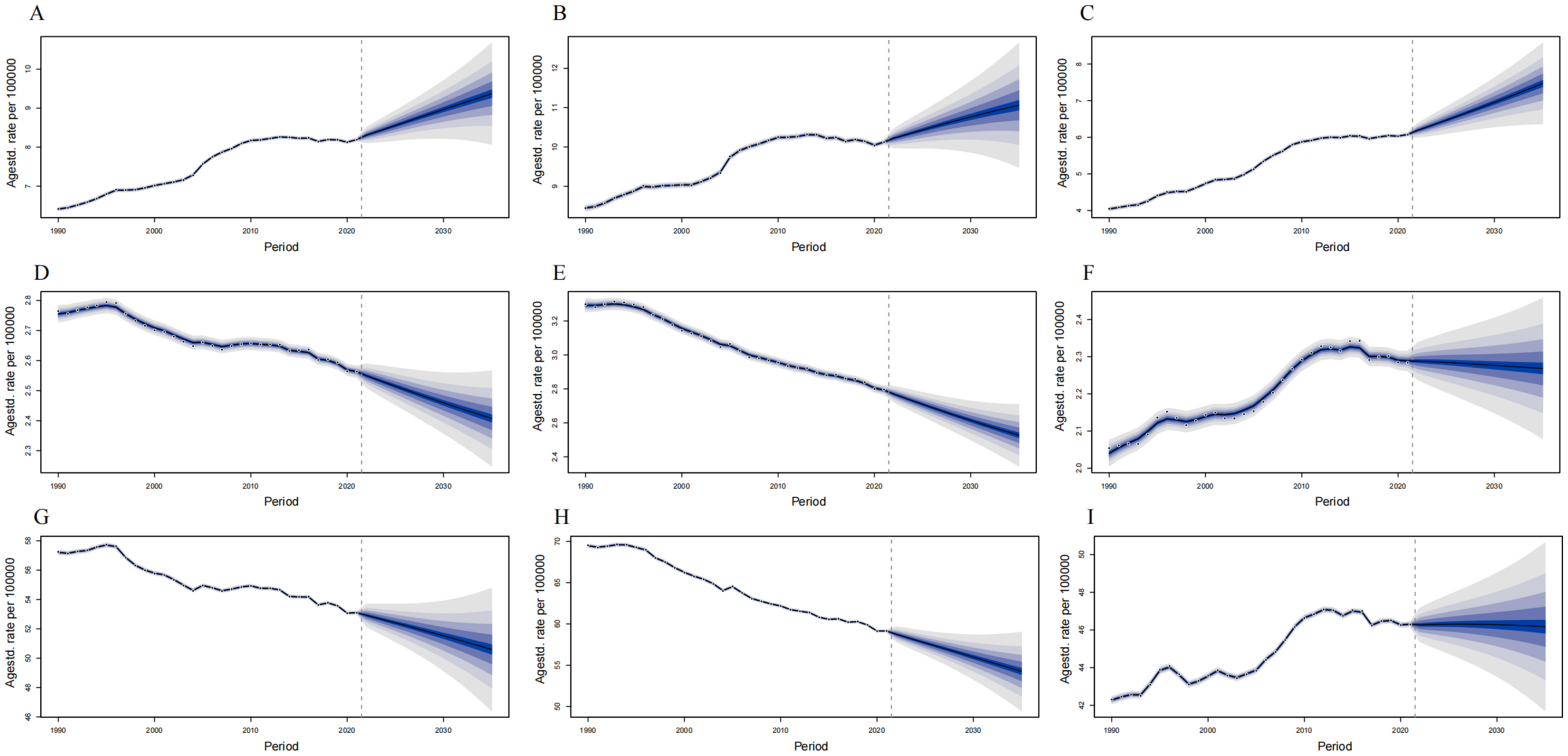
Predict the incidence, deaths and DALYs of thyroid cancer in middle-aged and elderly patients in global in 2035. **(A)** Incidence rate of female and male. **(B)** Incidence rate of female. **(C)** Incidence rate of male. **(D)** Deaths rate of female and male. **(E)** Deaths rate of female. **(F)** Deaths rate of male. **(G)** DALYs rate of female and male. **(H)** DALYs rate of female. **(I)** DALYs rate of male. The blue region shows the upper and lower limits of the 95% uncertainty intervals (95% UIs).

Using the BAPC model, we forecast the global ASIR, ASDR, and ASYR for TC over the next decade. The projections indicate that the global ASIR is expected to reach 9.37 per 100,000 population by 2035, which represents a 14.41% increase compared to the levels in 2021. Specifically, the increase is projected to be 9.18% for females and 22.86% for males. However, the global ASDR is estimated to be 2.41 per 100,000 population, reflecting a 5.86% decrease from 2021 to 2035. Additionally, the global ASYR is projected to be 50.60 per 100,000 population in 2035, a 4.1% decrease compared to the 2021 levels. It is noteworthy that the decline in both ASDR and ASYR is anticipated to be greater in males than in females (Figure 6).

## 4 Discussion

To our knowledge, this study is the first to comprehensively describe the burden of TC and its trends among individuals aged 55 and above at the global, regional, and national levels from 1990 to 2021, stratified by age, sex, and SDI. The results demonstrate a global increase in the incidence of TC among the middle-aged and elderly population over the 30-year study period, while mortality and DALYs have shown a declining trend. Additionally, our findings reveal a positive correlation between TC incidence in the middle-aged and elderly and SDI levels, though this relationship is less pronounced for mortality and DALYs. Females consistently exhibit higher incidence rates, a greater number of incident cases, and elevated mortality and DALY figures across all age groups compared to males. A particularly notable observation is the concentration of TC-related deaths in the 70–79 age group. These findings underscore the pressing need for enhanced awareness, early detection, and optimized management to address the mounting burden of thyroid cancer in an aging global population.

From 1990 to 2021, the incidence of TC increased steadily, with an EAPC of 0.95, indicating a persistent upward trend. Conversely, the EAPC for mortality and DALYs were −0.06 and −0.23, respectively, reflecting a declining trend. Projections for 2035 indicate a continued increase in the overall burden of TC, as reflected by a rising ASIR, despite the observed declines in age-standardized DALY rates and ASDR. Several factors contribute to these trends in the global TC burden. Advances in diagnostic technologies, such as the widespread adoption of imaging techniques and fine-needle aspiration biopsies, combined with increased health awareness, have likely led to an increase in diagnosed cases, inflating disease burden statistics (11, 12). Overdiagnosis also plays a significant role in the rising incidence of TC (13). Additionally, environmental factors, including radiation exposure, and lifestyle changes, such as obesity and dietary patterns, are contributing to the increase in TC incidence (14, 15). At the same time, enhanced understanding of TC and advancements in treatment modalities have contributed to declining global mortality and DALYs. For example, active surveillance has emerged as a viable option for patients with small, low-risk papillary thyroid carcinoma (4, 16). During active surveillance, patients undergo periodic neck ultrasound evaluations to monitor the thyroid and cervical lymph nodes for disease progression (17, 18). Longitudinal studies have shown that active surveillance is a safe alternative to immediate surgery for selected patients (19). Additionally, the development of minimally invasive techniques, including ultrasound-guided radiofrequency ablation, microwave ablation, and laser ablation, has provided effective and safe alternatives to surgery for treating papillary microcarcinoma of the thyroid (20, 21). The introduction of thyroid endoscopic surgery (22) and targeted therapies for advanced TC (23) further expands treatment options, enabling personalized approaches that align with patient needs.

Globally, both males and females have experienced an increasing trend in the incidence of TC. While females exhibit a higher incidence rate overall, the rate of increase in males is significantly steeper. This disparity may be partly influenced by abnormal levels of estrogen in females (24). Additionally, factors related to menopause could lead to increased healthcare engagement among women, resulting in more frequent thyroid examinations (12), which may contribute to the observed trends. Interestingly, despite a global decline in mortality and DALYs for TC, there is a distinct difference between genders. Mortality and DALYs in males are trending upward, whereas in females, they are decreasing. This divergence may partly be explained by the higher thyroid cancer burden observed in females compared with males, which may have contributed to greater emphasis on public health policies targeting female thyroid cancer (25), leading to public health policies that prioritize female TC. Economic development has also raised the risks associated with occupational exposure, which studies suggest may be linked to TC. Males are more likely to be employed in occupations associated with higher exposure risks (26). These findings highlight the need for tailored public health interventions targeting male TC, focusing on early detection and management to reduce the healthcare burden and improve life expectancy.

In 2021, the largest number of TC cases was recorded in East Asia, North America, and Western Europe, partly reflecting their large populations. An increasing incidence of TC was observed across all regions except Central Europe. This rise appears to be associated with the widespread adoption of imaging examinations, ultrasounds, and biopsies. Advances in diagnostic technologies have significantly improved the detection and diagnosis of early-stage TC (27). Consequently, the modernization of TC detection methods has contributed to the rising incidence of TC. To address the effects of overdiagnosis, organizations such as the American Thyroid Association (ATA) have implemented stricter guidelines for thyroid nodule biopsies (28, 29). With increased awareness of overdiagnosis, it is anticipated that the upward trend in incidence could reverse within the next few decades. China has the highest incidence, number of deaths, and DALYs for TC compared to other countries worldwide. Population growth and aging are major factors driving the continuous rise in the global cancer burden (30). A study projects that by 2050, China’s population aged 65 and above will reach 400 million, including 150 million individuals aged 80 and above (31). Managing the growing impact of an aging population on China’s public healthcare system will therefore present a significant challenge.

Our study found that the incidence of TC increases with higher SDI levels, whereas DALYs decrease as SDI rises. While no significant differences in mortality were observed across SDI regions, the ASDR in high-SDI and high-middle-SDI regions exhibits a downward trend. These findings suggest that the burden of TC is decreasing in areas with high SDI, while it continues to rise in low-SDI regions. This pattern may be attributed to superior medical infrastructure and advanced diagnostic and treatment technologies in high-SDI countries, which enable more timely and effective management of TC (32). Additionally, stronger public health awareness and a deeper understanding of disease prevention in these regions contribute to reducing the occurrence of TC. In contrast, countries in middle-SDI and low-middle-SDI regions are undergoing rapid urbanization and industrialization, leading to changes in dietary habits, increased stress levels, and increased exposure to environmental pollutants. These factors are likely driving the sharp increases in the incidence, mortality, and DALY rates of TC among the elderly population in these regions (33). The observed differences based on SDI emphasize the critical role of socioeconomic development in shaping public health outcomes. Tailored strategies that address the unique needs and resources of each SDI category can significantly improve the effectiveness of healthcare interventions aimed at reducing the burden of TC.

Our research identified high BMI as a significant risk factor for TC, with obesity contributing to the increasing incidence of the disease (34). Notably, a study indicated that the impact of obesity on TC mortality is greater than its effect on incidence (35). Additionally, a cross-sectional study demonstrated that aggressive clinical features of TC are associated with overweight and obesity (36). Further research suggests that diet-induced obesity directly influences aggressive and anaplastic changes in TC (37). Over recent decades, the global incidence, mortality, and DALYs associated with obesity have been on the rise, and projections indicate that the burden of malnutrition and obesity will continue to grow over the next decade (38). It is also anticipated that the increase in obesity burden among females will outpace that of males in the coming years. These findings highlight the importance of promoting a healthy lifestyle, including a balanced diet, regular physical activity, and the reduction of unhealthy habits, to lower the risk of developing TC.

Furthermore, the disease burden projected by the BAPC model should be interpreted with caution, as it is based on the core assumption that epidemiological trends observed from 1990 to 2021 will continue into the future. However, these projections have not been externally validated by training the model on earlier data and testing it against more recent observations. As a result, the uncertainty of the estimates increases with longer projection horizons. In addition, the actual future burden may be substantially affected by unpredictable factors, such as revisions to thyroid cancer diagnostic guidelines, the adoption and standardization of screening practices, and the effectiveness of public health policies targeting major risk factors like obesity. Therefore, the predicted values in this study should be considered as reference estimates under a “current trends unchanged” scenario, intended to highlight potential future challenges and to inform the prioritization of health policies, rather than to provide precise forecasts.

While our study explored the epidemiological characteristics, trends, and risk factors of TC in the middle-aged and elderly, several limitations should be acknowledged. First, the accuracy and completeness of GBD estimates may be affected by the data reporting systems and collection methods used by individual countries. Variations in diagnostic criteria across nations could also introduce bias into the analysis. Second, our study was limited to the risk factors included in the GBD database. Among the recognized risk factors for TC, the database only accounts for high BMI, preventing us from investigating associations and interactions between multiple risk factors. Lastly, due to constraints in data availability, the GBD database does not provide stratification by histological features, such as papillary, follicular, medullary, and anaplastic TC. Consequently, we were unable to examine how these histological subtypes vary by age, gender, region, and risk factors.

## 5 Conclusion

The increasing incidence of TC among middle-aged and older adults is placing a significant burden on global healthcare systems, a challenge expected to intensify with the ongoing acceleration of global population aging. A one-size-fits-all approach to early screening and diagnosis is unlikely to address this issue effectively. Instead, tailored screening and treatment strategies must be developed to account for differences across gender groups and regions. It is equally important to deepen our understanding of TC risk factors. Preventive measures, such as controlling obesity and minimizing radiation exposure, can help reduce the public health burden associated with TC. Revising the “Guidelines for the Diagnosis and Treatment of Thyroid Cancer” and implementing targeted strategies to reverse the upward trend in TC incidence are therefore critical steps in addressing this growing challenge.

## Data Availability

The original contributions presented in the study are included in the article/Supplementary material, further inquiries can be directed to the corresponding author/s.

## Footnotes

^1^ https://vizhub.healthdata.org/gbd-results/
