# Correction: Global, regional, and national epidemiology of thyroid cancer in middle-aged and elderly adults from 1990 to 2021

**DOI:** 10.3389/fmed.2025.1735517

**Published:** 2025-11-14

**Authors:** Ling Wang, LinZhi Liao, JueZhi Huang, Qi Zhang, YanQing Xiong, FuYu Tian, Xin Liu, YanJia Liu, LuYun Jiang, Yan Xie

**Affiliations:** 1 Clinical Medical College, Chengdu University of Traditional Chinese Medicine, Chengdu, Sichuan, China; 2 Department of Otorhinolaryngology, Hospital of Chengdu University of Traditional Chinese Medicine, Chengdu, China

**Keywords:** thyroid cancer, middle-aged and elderly, global burden of disease, incidence, mortality, disability-adjusted life years

Authors Ling Wang and LinZhi Liao were erroneously omitted as being equal contributing authors. They should both have the attribution “These authors contributed equally to this work and share first authorship.”

The original version of this article has been updated.

