# Supplementary material for: Global, regional, and national epidemiology of thyroid cancer in middle-aged and elderly adults from 1990 to 2021
Source: Front Med (Lausanne). 2025 Oct 21;12:1684535. doi: 10.3389/fmed.2025.1684535 (PMC12583101; doi:10.3389/fmed.2025.1684535)
Supplement: Supplementary file 1 [file Data_Sheet_1.docx]

**Supplementary Online Content**

**Figure S1**. Incidence, deaths and DALYs rates of thyroid cancer in middle-aged and elderly patients in 204 countries by SDI in 2021. (A) Incidence rate. (B) Deaths rate. (C) DALYs rate. DALYs=disability-adjusted life-years; SDI= sociodemographic index.

**Figure S2**. The deaths for thyroid cancer in middle-aged and elderly patients in 204 countries and territories from 1990 to 2021. (A).The number of deaths cases. (B). Disease burden of deaths rate. (C). EAPC for deaths rate. DALY=disability-adjusted life-year.

**Figure S3**. The DALYs for thyroid cancer in middle-aged and elderly patients in 204 countries and territories from 1990 to 2021. (A).The number of DALYs cases. (B). Disease burden of DALYs rate. (C). EAPC for DALYs rate.

**Figure S4**. Global trends by gender and age groups for thyroid cancer in middle-aged and elderly patients from 1990 to 2021. (A) incidence rate of female and male. (B) incidence rate of female. (C) incidence rate of male. (D) deaths rate of female and male. (E) deaths rate of female. (F) deaths rate of male. (G) DALYs rate of female and male. (H) DALYs rate of female. (I) DALYs rate of male.

**Figure S5**. Percentage of deaths(A) and DALYs(B) due to thyroid cancer in middle-aged and elderly patients attributable to each risk factor for the Global Burden of Disease regions.

**Table S1**. The death cases and rates for thyroid cancer in middle-aged and elderly patients in 1990/2021 and its temporal trends.

**Table S2**. The DALYs cases and rates for thyroid cancer in middle-aged and elderly patients in 1990/2021 and its temporal trends.

**Table S3.** The incidence cases and rates for thyroid cancer in middle-aged and elderly patients in 2021 in various countries.

**Table S4.** The deaths cases and rates for thyroid cancer in middle-aged and elderly patients in 2021 in various countries.

**Table S5.** The DALYs cases and rates for thyroid cancer in middle-aged and elderly patients in 2021 in various countries.

**Table S6.** Estimated annual percentage change (EAPC) in incidence rates of thyroid cancer for both sex by Country, 1990-2021.

**Table S7.** Estimated annual percentage change (EAPC) in deaths rates of thyroid cancer for both sex by Country, 1990-2021.

**Table S8.** Estimated annual percentage change (EAPC) in DALYs rates of thyroid cancer for both sex by Country, 1990-2021.

**Table S9.** Global age and sex structure of thyroid cancer incidence, deaths and DALYs, 2021.

This supplementary material has been provided by the authors to give readers additional information about their work.


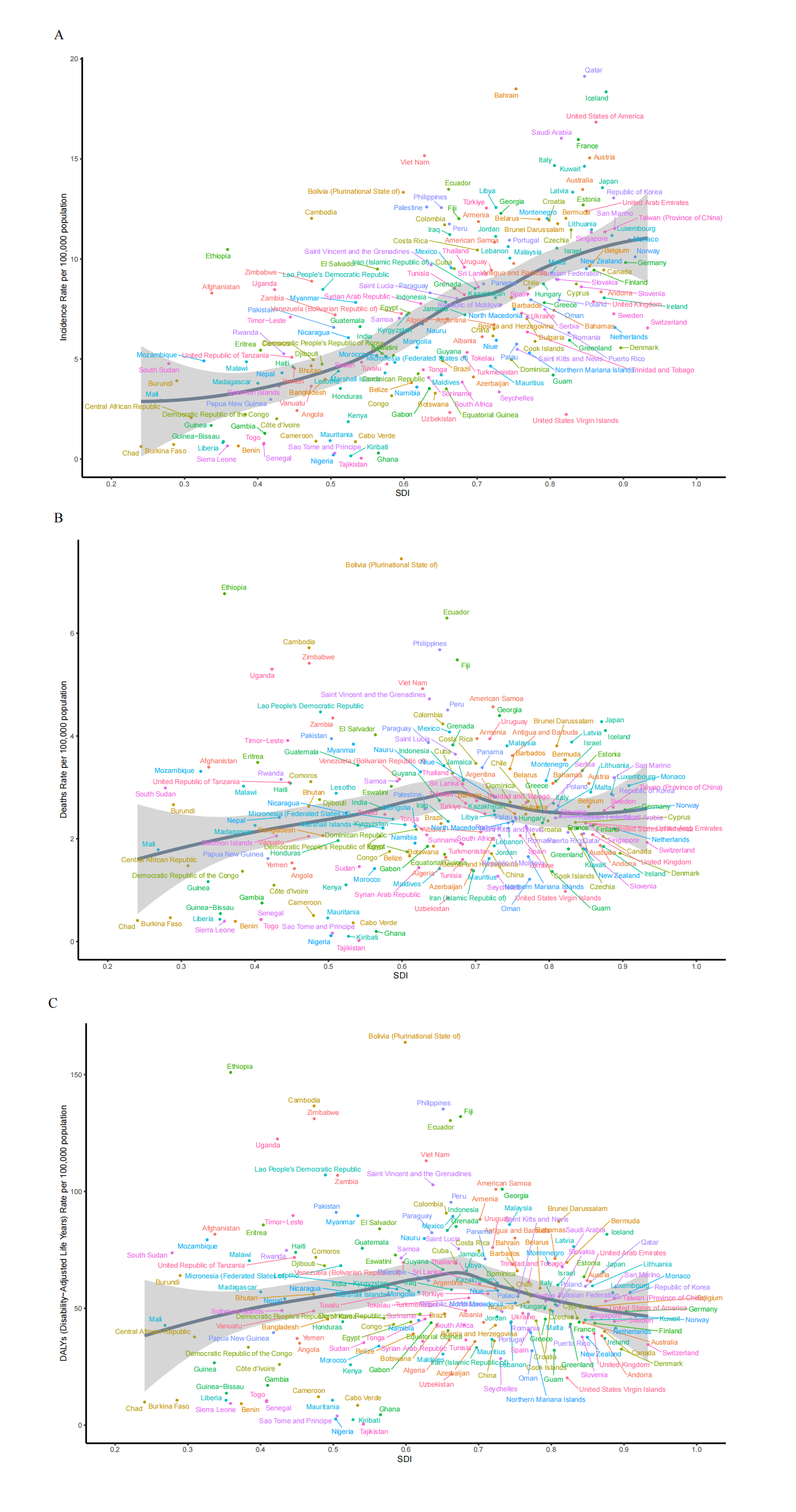


Figure S1. Incidence, deaths and DALYs rates of thyroid cancer in middle-aged and elderly patients in 204 countries by SDI in 2021. (A) Incidence rate. (B) Deaths rate. (C) DALYs rate. DALYs=disability-adjusted life-years; SDI= sociodemographic index.


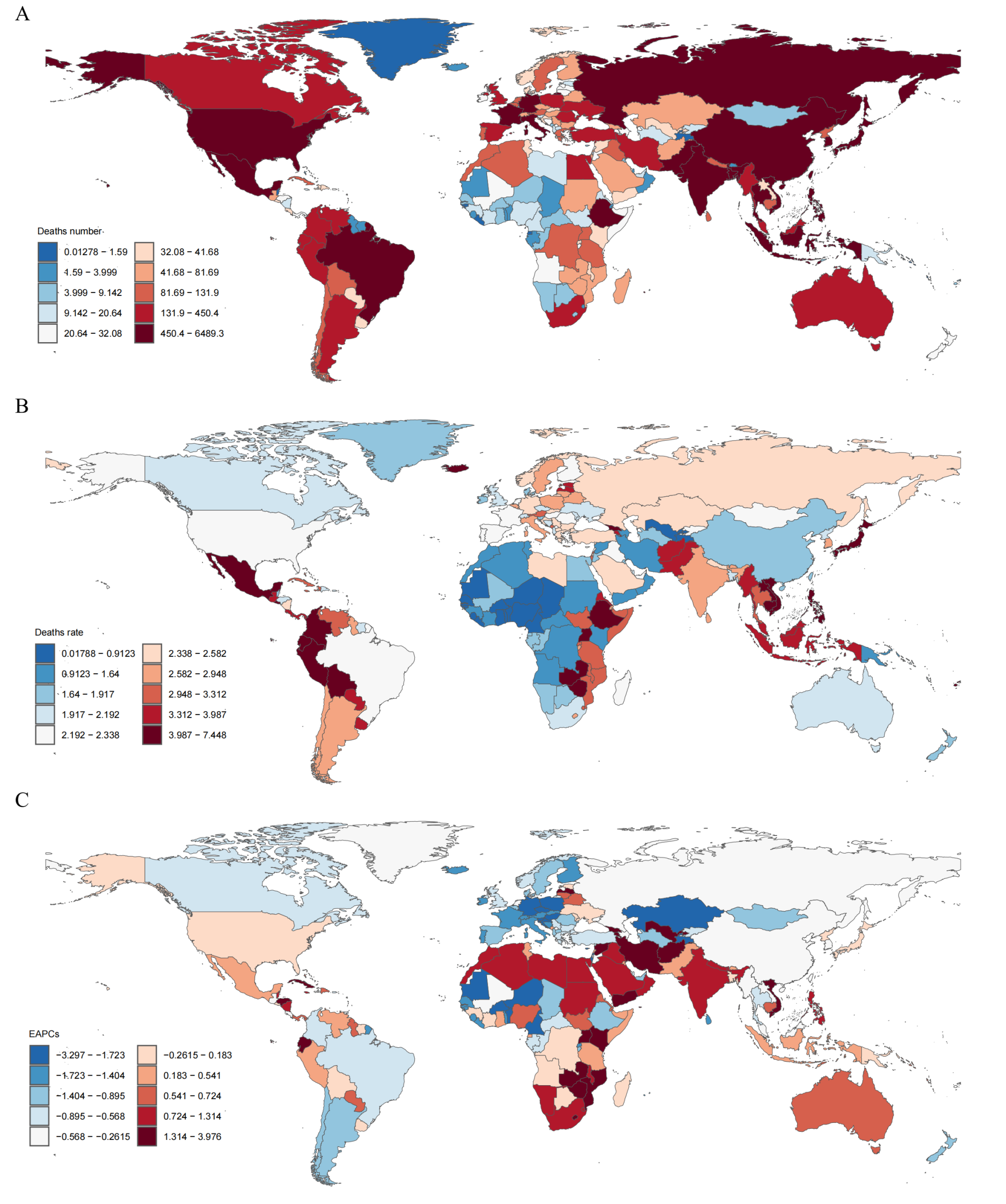


Figure S2. The deaths for thyroid cancer in middle-aged and elderly patients in 204 countries and territories from 1990 to 2021. (A).The number of deaths cases. (B). Disease burden of deaths rate. (C). EAPC for deaths rate. DALY=disability-adjusted life-year.


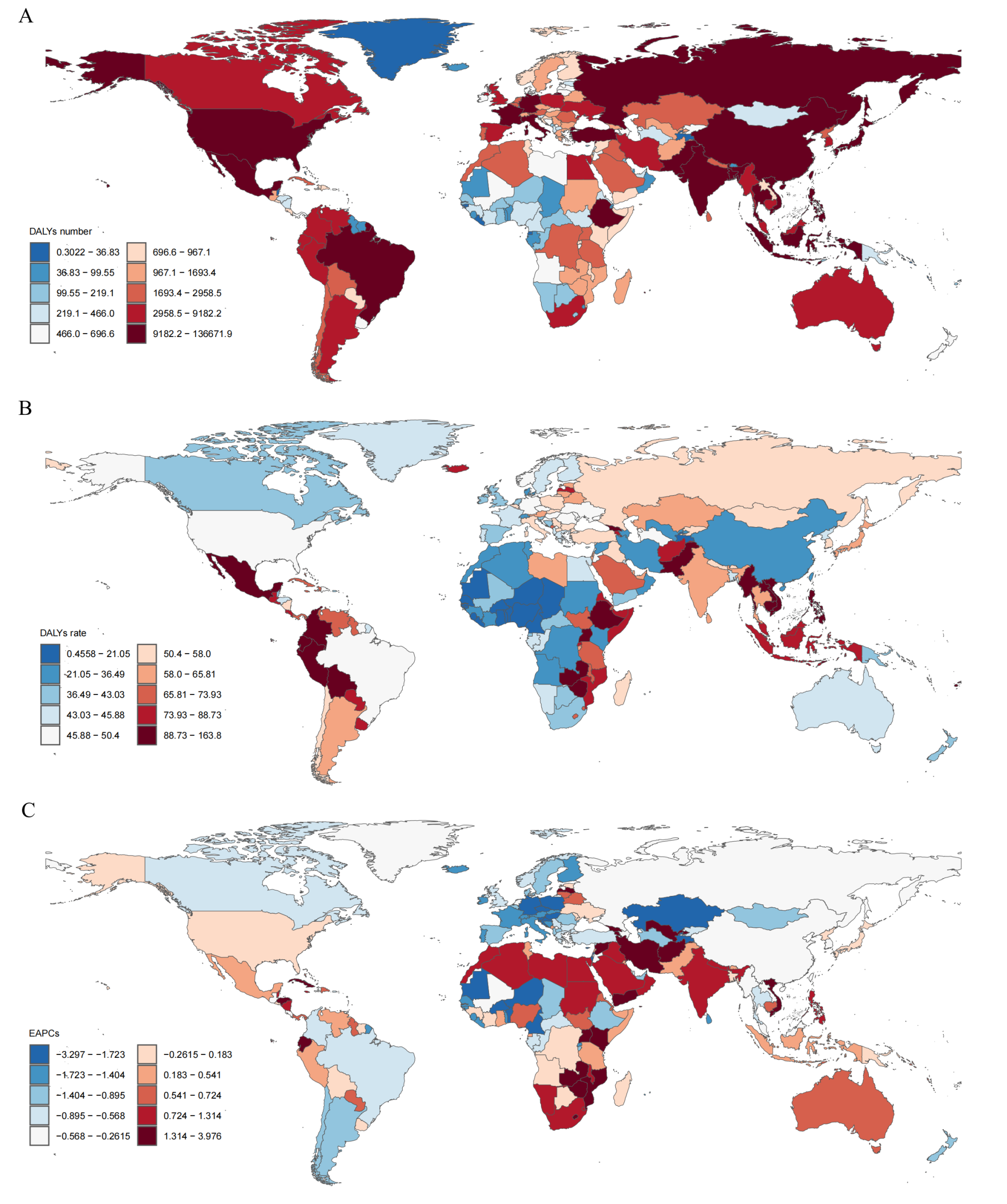


Figure S3. The DALYs for thyroid cancer in middle-aged and elderly patients in 204 countries and territories from 1990 to 2021. (A).The number of DALYs cases. (B). Disease burden of DALYs rate. (C). EAPC for DALYs rate.


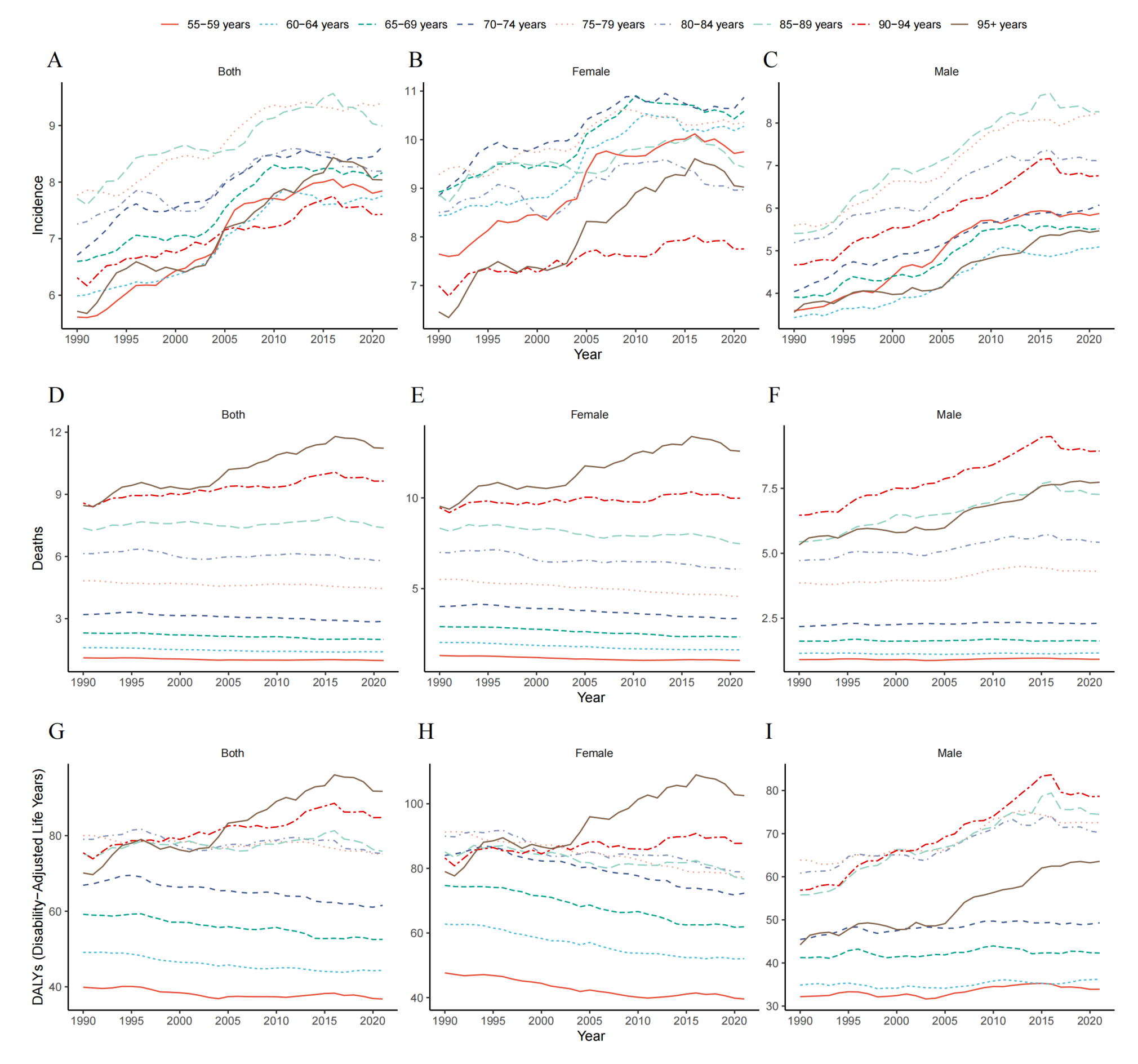


Figure S4. Global trends by gender and age groups for thyroid cancer in middle-aged and elderly patients from 1990 to 2021. (A) incidence rate of female and male. (B) incidence rate of female. (C) incidence rate of male. (D) deaths rate of female and male. (E) deaths rate of female. (F) deaths rate of male. (G) DALYs rate of female and male. (H) DALYs rate of female. (I) DALYs rate of male.


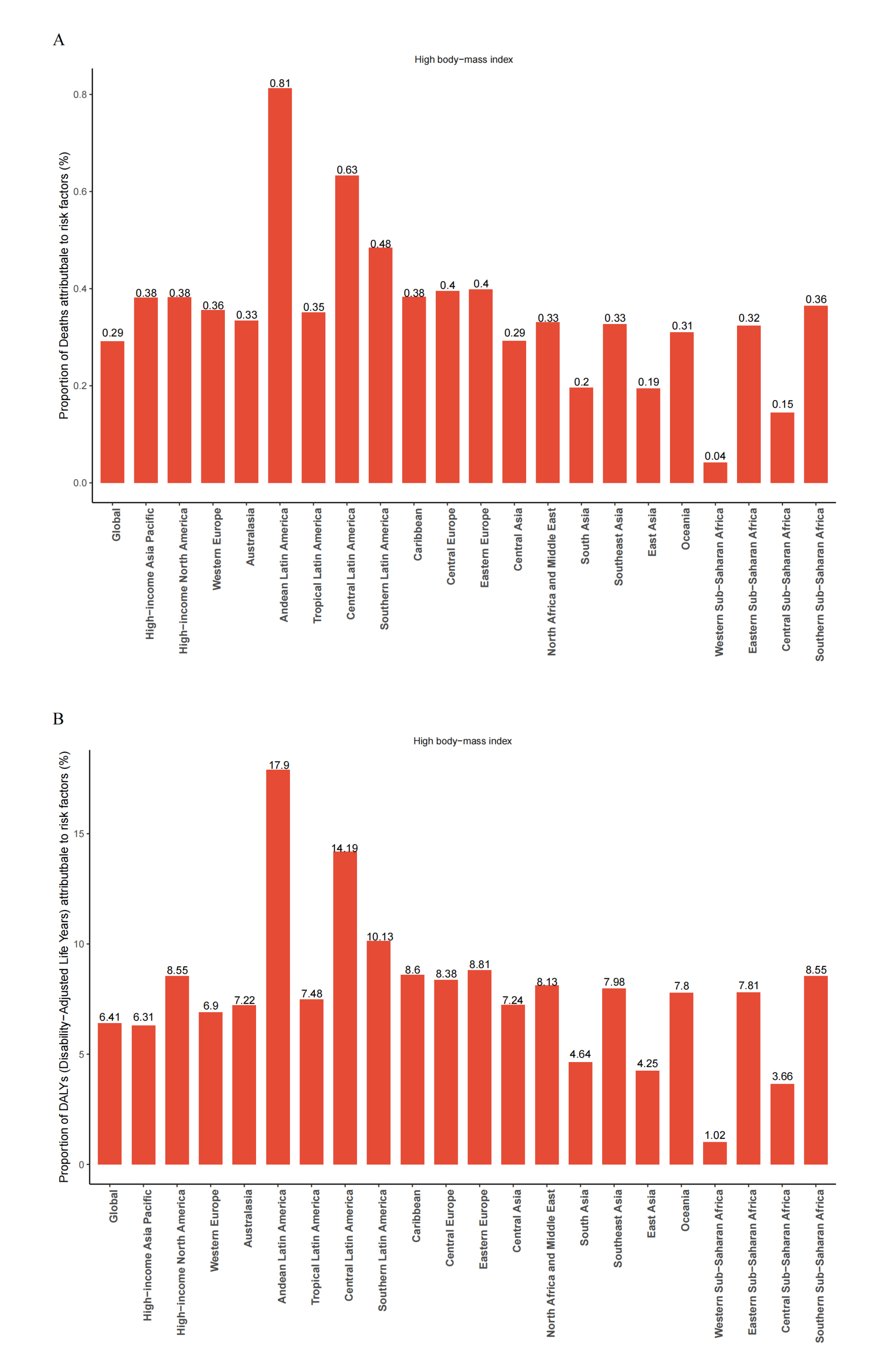


Figure S5. Percentage of deaths(A) and DALYs(B) due to thyroid cancer in middle-aged and elderly patients attributable to each risk factor for the Global Burden of Disease regions.

Table S1. The death cases and rates for thyroid cancer in middle-aged and elderly patients in 1990/2021 and its temporal trends.

| location | Rate per 100,000 (95% UI) | | | | | |
| --- | --- | --- | --- | --- | --- | --- |
|  | 1990 | | 2021 | | 1990-2021 | |
|  | cases | rate | cases | rate | Cases change | EAPC* |
| Global | 16891.09  (15743.67,18564.92) | 2.52  (2.34,2.77) | 36442.04  (32220.59,39277.83) | 2.45  (2.17,2.64) | 1.16  (0.97,1.32) | -0.06  (-0.08, -0.03) |
| Gender: |  |  |  |  |  |  |
| Male | 5460.83  (5136.90,6095.46) | 1.75  (1.65,1.96) | 14420.29  (12243.51,15845.93) | 2.06  (1.75,2.27) | 1.64  (1.26,1.92) | 0.57  (0.53,0.61) |
| Female | 11430.26  (10414.26,12988.58) | 3.18  (2.89,3.61) | 22021.75  (19066.42,25221.61) | 2.80  (2.42,3.21) | 0.93  (0.74,1.10) | -0.37  (-0.41, -0.33) |
| SDI level: |  |  |  |  |  |  |
| High | 5711.35  (5309.13,5992.45) | 3.06  (2.85,3.21) | 9002.16  (7748.65,9697.92) | 2.61  (2.25,2.81) | 0.58  (0.45,0.66) | -0.49  (-0.52, -0.45) |
| High-middle | 4517.54  (4174.97,4787.24) | 2.62  (2.42,2.77) | 7316.50  (6527.17,8030.71) | 2.11  (1.88,2.32) | 0.62  (0.48,0.77) | -0.44  (-0.55, -0.34) |
| Middle | 3787.05  (3462.13,4476.62) | 2.18  (1.99,2.58) | 11789.26  (10098.38,13014.63) | 2.51  (2.15,2.77) | 2.11  (1.66,2.52) | 0.39  (0.35,0.42) |
| Low-middle | 1960.45  (1709.82,2423.57) | 1.94  (1.70,2.40) | 6156.25  (5407.83,6931.74) | 2.55  (2.24,2.88) | 2.14  (1.65,2.63) | 0.90  (0.86,0.94) |
| Low | 887.84  (734.76,1084.20) | 2.38  (1.97,2.91) | 2143.00  (1724.01,2629.53) | 2.61  (2.10,3.20) | 1.41  (0.97,1.96) | 0.27  (0.23,0.31) |
| GBD Regions: |  |  |  |  |  |  |
| Andean Latin America | 139.45  (116.37,162.90) | 4.16  (3.47,4.85) | 541.41  (423.43,669.82) | 5.47  (4.27,6.76) | 2.88  (2.09,3.88) | 0.89  (0.80,0.97) |
| Australasia | 85.06  (75.92,95.50) | 2.16  (1.93,2.42) | 180.24  (146.28,214.74) | 2.04  (1.66,2.43) | 1.12  (0.72,1.61) | 0.01  (-0.19,0.20) |
| Caribbean | 109.13  (100.84,118.19) | 2.53  (2.34,2.74) | 262.27  (229.75,298.43) | 2.83  (2.48,3.22) | 1.40  (1.12,1.73) | 0.11  (-0.07,0.30) |
| Central Asia | 179.09  (166.05,194.72) | 2.24  (2.08,2.43) | 265.97  (237.70,295.48) | 1.83  (1.63,2.03) | 0.49  (0.31,0.70) | -0.74  (-1.02, -0.46) |
| Central Europe | 1052.91  (1005.85,1095.68) | 3.97  (3.79,4.13) | 896.50  (814.54,968.45) | 2.42  (2.20,2.62) | -0.15  (-0.22, -0.08) | -1.45  (-1.66, -1.24) |
| Central Latin America | 490.79  (469.75,508.99) | 3.62  (3.46,3.75) | 1652.39  (1467.22,1821.40) | 3.86  (3.43,4.26) | 2.37  (2.02,2.72) | -0.19  (-0.31, -0.07) |
| Central Sub-Saharan Africa | 55.63  (40.33,81.82) | 1.48  (1.07,2.18) | 127.58  (81.72,200.90) | 1.41  (0.91,2.23) | 1.29  (0.69,2.03) | -0.05  (-0.10,0.01) |
| East Asia | 2783.37  (2389.84,3228.68) | 1.87  (1.60,2.17) | 6796.79  (5437.46,8217.29) | 1.73  (1.39,2.10) | 1.44  (0.98,2.09) | -0.27  (-0.33, -0.22) |
| Eastern Europe | 1107.99  (1038.75,1177.23) | 2.27  (2.12,2.41) | 1467.81  (1327.30,1612.94) | 2.36  (2.14,2.60) | 0.32  (0.19,0.47) | 0.70  (0.40,1.00) |
| Eastern Sub-Saharan Africa | 495.31  (407.97,591.10) | 4.07  (3.35,4.86) | 1065.74  (793.39,1403.45) | 3.94  (2.93,5.19) | 1.15  (0.66,1.81) | -0.20  (-0.25, -0.15) |
| High-income Asia Pacific | 1104.98  (1004.02,1255.13) | 3.16  (2.87,3.59) | 2746.64  (2212.36,3084.84) | 3.90  (3.14,4.38) | 1.49  (1.14,1.74) | 0.53  (0.44,0.61) |
| High-income North America | 1221.89  (1118.21,1278.03) | 2.11  (1.93,2.21) | 2537.62  (2246.18,2716.31) | 2.25  (2.00,2.41) | 1.08  (0.99,1.17) | 0.23  (0.13,0.33) |
| North Africa and Middle East | 463.26  (381.12,666.60) | 1.64  (1.35,2.36) | 1438.47  (1250.06,1676.44) | 1.89  (1.64,2.20) | 2.11  (1.32,2.87) | 0.64  (0.55,0.73) |
| Oceania | 10.23  (7.28,13.58) | 2.13  (1.51,2.82) | 26.53  (17.43,35.98) | 2.15  (1.41,2.92) | 1.59  (1.09,2.27) | 0.23  (0.14,0.32) |
| South Asia | 1772.98  (1480.65,2239.39) | 1.87  (1.56,2.36) | 6565.24  (5541.37,7524.38) | 2.64  (2.23,3.03) | 2.70  (1.96,3.41) | 1.09  (1.04,1.14) |
| Southeast Asia | 1464.33  (1248.05,1748.74) | 3.46  (2.95,4.13) | 4500.03  (3667.85,5158.09) | 3.93  (3.20,4.50) | 2.07  (1.63,2.56) | 0.60  (0.49,0.72) |
| Southern Latin America | 297.98  (268.25,325.77) | 3.76  (3.39,4.11) | 423.00  (369.67,484.65) | 2.87  (2.51,3.29) | 0.42  (0.23,0.64) | -0.61  (-0.76, -0.47) |
| Southern Sub-Saharan Africa | 82.58  (66.90,102.33) | 1.87  (1.51,2.31) | 228.13  (183.52,257.38) | 2.34  (1.89,2.64) | 1.76  (1.19,2.33) | 0.87  (0.68,1.06) |
| Tropical Latin America | 381.09  (356.21,403.45) | 2.52  (2.35,2.66) | 1032.73  (927.47,1102.10) | 2.33  (2.09,2.49) | 1.71  (1.50,1.89) | -0.35  (-0.42, -0.27) |
| Western Europe | 3527.97  (3248.84,3736.61) | 3.63  (3.35,3.85) | 3563.17  (3064.55,3908.40) | 2.39  (2.05,2.62) | 0.01  (-0.07,0.10) | -1.15  (-1.20, -1.10) |
| Western Sub-Saharan Africa | 65.07  (49.02,77.32) | 0.45  (0.34,0.54) | 123.80  (100.47,154.63) | 0.39  (0.31,0.48) | 0.90  (0.51,1.45) | -0.30  (-0.41, -0.18) |

Abbreviations: EAPC, estimated annual percentage change; SDI, Sociodemographic Index; UI, uncertainty interval.

*EAPC is expressed as 95% Cis.

| location | Rate per 100,000 (95% UI) | | | | | |
| --- | --- | --- | --- | --- | --- | --- |
|  | 1990 | | 2021 | | 1990-2021 | |
|  | DALYs cases | DALYs rate | DALYs cases | DALYs rate | Cases change | EAPC* |
| Global | 375067.84  (351621.47,412720.98) | 55.86  (52.37,61.47) | 779087.94  (695795.16,843551.32) | 52.43  (46.82,56.77) | 1.08  (0.90,1.24) | -0.23  (-0.26, -0.21) |
| Gender: |  |  |  |  |  |  |
| Male | 125872.48  (118064.31,139544.41) | 40.41  (37.90,44.80) | 313195.58  (265312.97,345077.71) | 44.77  (37.93,49.33) | 1.49  (1.13,1.75) | 0.40  (0.34,0.45) |
| Female | 249195.36  (227141.72,283305.16) | 69.23  (63.11,78.71) | 465892.36  (401264.29,530861.47) | 59.24  (51.02,67.50) | 0.87  (0.69,1.06) | -0.59  (-0.62, -0.55) |
| SDI level: |  |  |  |  |  |  |
| High | 119284.92  (112437.77,125161.98) | 63.97  (60.30,67.12) | 172679.98  (154919.68,185861.18) | 50.05  (44.90,53.87) | 0.45  (0.37,0.52) | -0.78  (-0.83, -0.72) |
| High-middle | 100985.33  (94028.67,107106.05) | 58.53  (54.50,62.08) | 155149.69  (138937.36,171739.29) | 44.75  (40.08,49.54) | 0.54  (0.40,0.69) | -0.95  (-1.03, -0.87) |
| Middle | 86544.33  (78990.37,101667.85) | 49.86  (45.51,58.58) | 261019.53  (222137.03,287484.24) | 55.55  (47.28,61.19) | 2.02  (1.58,2.41) | 0.31  (0.27,0.36) |
| Low-middle | 46135.20  (40380.15,56434.99) | 45.77  (40.06,55.99) | 140020.18  (121161.89,156960.63) | 58.08  (50.26,65.11) | 2.03  (1.57,2.51) | 0.78  (0.74,0.82) |
| Low | 21515.00  (17930.74,26150.18) | 57.67  (48.06,70.09) | 49465.69  (39783.96,61160.91) | 60.28  (48.48,74.53) | 1.30  (0.87,1.84) | 0.11  (0.04,0.18) |
| GBD Regions: |  |  |  |  |  |  |
| Andean Latin America | 3043.20  (2508.07,3585.55) | 90.68  (74.74,106.84) | 11472.36  (8865.84,14353.10) | 115.81  (89.50,144.89) | 2.77  (2.00,3.84) | 0.77  (0.66,0.88) |
| Australasia | 1808.81  (1621.78,2028.44) | 45.91  (41.17,51.49) | 3787.21  (3118.86,4496.57) | 42.87  (35.30,50.90) | 1.09  (0.71,1.54) | 0.33  (0.06,0.59) |
| Caribbean | 2369.89  (2194.38,2596.70) | 54.99  (50.92,60.25) | 5672.40  (4989.71,6491.15) | 61.27  (53.89,70.11) | 1.39  (1.11,1.74) | 0.51  (0.27,0.75) |
| Central Asia | 4333.25  (4003.87,4727.05) | 54.18  (50.06,59.10) | 6448.63  (5738.96,7185.34) | 44.32  (39.44,49.38) | 0.49  (0.30,0.71) | -0.83  (-1.38, -0.28) |
| Central Europe | 23956.73  (22873.87,25051.92) | 90.33  (86.25,94.46) | 18616.38  (16967.58,20156.14) | 50.28  (45.82,54.43) | -0.22  (-0.29,-0.16) | -2.15  (-2.46, -1.85) |
| Central Latin America | 10636.96  (10241.44,11044.68) | 78.39  (75.47,81.39) | 35987.84  (32246.05,39899.45) | 84.15  (75.40,93.30) | 2.38  (2.02,2.75) | 0.03  (-0.16,0.21) |
| Central Sub-Saharan Africa | 1388.62  (999.24,2047.69) | 36.93  (26.57,54.46) | 3126.27  (1992.23,4875.52) | 34.65  (22.08,54.03) | 1.25  (0.65,2.00) | -0.24  (-0.33, -0.16) |
| East Asia | 63107.80  (53790.84,73648.14) | 42.37  (36.11,49.44) | 143247.88  (115994.95,174708.65) | 36.53  (29.58,44.55) | 1.27  (0.83,1.91) | -0.51  (-0.60, -0.43) |
| Eastern Europe | 25345.29  (23836.40,27007.86) | 51.84  (48.75,55.24) | 31766.91  (28842.71,35142.01) | 51.17  (46.46,56.61) | 0.25  (0.12,0.39) | -0.20  (-0.60,0.19) |
| Eastern Sub-Saharan Africa | 12041.15  (9907.71,14436.60) | 98.98  (81.44,118.67) | 24643.94  (18251.55,32829.55) | 91.15  (67.50,121.42) | 1.05  (0.57,1.71) | -0.39  (-0.49, -0.30) |
| High-income Asia Pacific | 23309.71  (21416.70,26427.45) | 66.66  (61.25,75.58) | 44081.65  (37262.70,49751.55) | 62.52  (52.85,70.57) | 0.89  (0.67,1.06) | -0.18  (-0.32, -0.04) |
| High-income North America | 26736.21  (24977.75,28316.94) | 46.15  (43.12,48.88) | 55149.98  (50345.89,59339.49) | 49.01  (44.74,52.73) | 1.06  (0.98,1.14) | 0.07  (-0.03,0.18) |
| North Africa and Middle East | 10827.60  (8926.07,15455.57) | 38.31  (31.58,54.68) | 34446.96  (29672.19,40124.65) | 45.19  (38.92,52.63) | 2.18  (1.35,3.00) | 0.75  (0.62,0.89) |
| Oceania | 250.99  (177.34,338.37) | 52.17  (36.86,70.33) | 641.37  (410.15,893.03) | 51.97  (33.23,72.36) | 1.56  (1.04,2.25) | -0.06  (-0.11, -0.01) |
| South Asia | 42088.04  (35379.11,53041.85) | 44.33  (37.26,55.87) | 148932.10  (124787.50,170245.54) | 59.98  (50.26,68.57) | 2.54  (1.83,3.20) | 1.00  (0.95,1.05) |
| Southeast Asia | 34278.76  (28963.21,40048.74) | 80.96  (68.40,94.59) | 104999.83  (83334.61,120870.25) | 91.66  (72.75,105.51) | 2.06  (1.59,2.54) | 0.33  (0.22,0.44) |
| Southern Latin America | 6422.34  (5791.97,7055.42) | 81.08  (73.12,89.07) | 8627.64  (7537.52,9859.90) | 58.63  (51.22,67.00) | 0.34  (0.17,0.57) | -0.98  (-1.24, -0.73) |
| Southern Sub-Saharan Africa | 1815.10  (1471.34,2265.36) | 41.02  (33.25,51.20) | 5229.41  (4271.33,5905.39) | 53.72  (43.87,60.66) | 1.88  (1.28,2.51) | 0.98  (0.67,1.28) |
| Tropical Latin America | 8491.99  (7993.59,8964.63) | 56.08  (52.79,59.21) | 21448.08  (19641.72,22805.74) | 48.42  (44.34,51.48) | 1.53  (1.34,1.71) | -0.67  (-0.78, -0.56) |
| Western Europe | 71294.90  (66944.95,75453.71) | 73.41  (68.94,77.70) | 67839.83  (60113.32,74412.75) | 45.49  (40.31,49.90) | -0.05  (-0.12,0.03) | -1.39  (-1.48, -1.31) |
| Western Sub-Saharan Africa | 1520.51  (1131.53,1813.37) | 10.53  (7.84,12.56) | 2921.26  (2340.88,3645.36) | 9.09  (7.28,11.34) | 0.92  (0.52,1.50) | -0.61  (-0.71, -0.52) |

Table S2. The DALYs cases and rates for thyroid cancer in middle-aged and elderly patients in 1990/2021 and its temporal trends.

Abbreviations: EAPC, estimated annual percentage change; SDI, Sociodemographic Index; UI, uncertainty interval.

*EAPC is expressed as 95% Cis.

Table S3. The incidence cases and rates for thyroid cancer in middle-aged and elderly patients in 2021 in various countries.

| location | Number | | | Rate | | |
| --- | --- | --- | --- | --- | --- | --- |
|  | val | upper | lower | val | upper | lower |
| Micronesia (Federated States of) | 0.6359976214527305 | 0.8853672210146046 | 0.4212893433564926 | 4.833115616 | 6.728141737420001 | 3.201490124 |
| Azerbaijan | 78.6362124378086 | 102.7419966427632 | 58.03635690803856 | 4.104616742 | 5.362879346 | 3.029354986 |
| Thailand | 1863.166519201095 | 2880.84915880964 | 1332.121851286338 | 9.585182734 | 14.82071803 | 6.853188503 |
| Samoa | 1.732783321340659 | 3.155497737895772 | 1.14380062167262 | 7.037674879 | 12.8160093 | 4.64553 |
| Kazakhstan | 255.7612784705646 | 311.896308720394 | 206.4352963735808 | 8.059418825 | 9.828317238 | 6.505083661480001 |
| Indonesia | 3283.740797509753 | 4374.516530418382 | 2040.362109721588 | 7.840917049 | 10.4454716 | 4.871977126 |
| Viet Nam | 2648.762778968866 | 3603.176292813905 | 1905.617973094846 | 15.16300101 | 20.62659828 | 10.90882411 |
| China | 23319.10681771843 | 28913.04274902585 | 18653.94082658323 | 6.153527233 | 7.629674556 | 4.922466961 |
| Malaysia | 520.58678053375 | 703.8302747916343 | 416.2830727079033 | 10.6181744 | 14.35570953 | 8.490738586 |
| Tonga | 0.5992159376640883 | 0.8638833611823353 | 0.3840654101039668 | 4.461601777 | 6.432244701 | 2.859648432 |
| Mongolia | 22.01906676617323 | 29.55259568361834 | 16.39845974027114 | 5.577185571 | 7.485344951 | 4.153548105550001 |
| Fiji | 16.42780066018526 | 23.35369098402081 | 9.929672761446412 | 12.01384086 | 17.07882466 | 7.261684679249999 |
| Myanmar | 660.7962384856535 | 882.2221585262616 | 439.3453448388592 | 7.827370211 | 10.45024024 | 5.204204359 |
| Taiwan (Province of China) | 854.8143788206842 | 1001.66511581597 | 726.0680676254681 | 11.35221443 | 13.30244023 | 9.642421322 |
| Turkmenistan | 36.13725864451904 | 47.80229605517968 | 27.26028666735289 | 5.106288749 | 6.754588911 | 3.851949493 |
| Marshall Islands | 0.2522223877839449 | 0.3616409021395896 | 0.1614830181416877 | 4.304876851 | 6.172408253 | 2.756157028 |
| Armenia | 93.35687610783413 | 121.291862291412 | 71.49579800537934 | 11.86811975 | 15.41939283 | 9.089000483 |
| Sri Lanka | 414.161905376963 | 598.5611694040525 | 257.3401234768799 | 8.599163955 | 12.4278104 | 5.343103471 |
| Papua New Guinea | 24.47726080242808 | 42.12945337750902 | 13.619545218924 | 2.980296282 | 5.129587591 | 1.658285227 |
| Timor-Leste | 10.07712182451417 | 14.91898642110835 | 6.272177990631778 | 7.095011124 | 10.50402848 | 4.416059802 |
| Uzbekistan | 108.2910415490486 | 144.8231661680883 | 81.41260012106247 | 2.329515516 | 3.115380624 | 1.751316752 |
| Cambodia | 261.4956526965892 | 372.6815492615194 | 157.0452233366051 | 12.02936492 | 17.14415635 | 7.224419529 |
| Bulgaria | 166.7734780886826 | 211.3724504595677 | 129.6664499654817 | 7.016405185440001 | 8.892749461 | 5.455258008 |
| Georgia | 128.9119286523143 | 163.2317512330361 | 103.8379700532681 | 12.27886391 | 15.54782773 | 9.890568828 |
| Solomon Islands | 1.974903896050321 | 3.085008925928178 | 1.194455190225889 | 3.640662833 | 5.687100703 | 2.201934294 |
| Lao People's Democratic Republic | 65.54870794413316 | 92.27386855496226 | 40.7386194947679 | 8.478146809 | 11.93481045 | 5.269180854 |
| Kyrgyzstan | 60.97318567638379 | 80.1651771252327 | 44.75928399218603 | 7.209359506 | 9.478585962 | 5.292257014 |
| Albania | 48.0575436422592 | 69.07535275336264 | 34.58877387951399 | 6.111904781 | 8.784926294 | 4.39896167 |
| Vanuatu | 1.044101748031119 | 1.442807446851314 | 0.6813221084286292 | 3.641193816 | 5.031637542 | 2.376038401 |
| Maldives | 2.286506348132064 | 2.980544455483388 | 1.642918650526552 | 4.203414655 | 5.479304379 | 3.020270789 |
| Kiribati | 0.0195852668475054 | 0.040274916513552 | 0.0128256234494683 | 0.15572481 | 0.320230701 | 0.101978073 |
| Tajikistan | 0.4381114379869845 | 0.6767169788300179 | 0.2816621967967564 | 0.042653321 | 0.065883299 | 0.027421855 |
| Philippines | 1752.236890060359 | 2141.529661361932 | 1349.836826859412 | 12.56527668 | 15.35689202 | 9.679669053 |
| Democratic People's Republic of Korea | 297.6396303202893 | 399.040074044293 | 208.123541687448 | 5.281260057 | 7.080489926 | 3.692903886 |
| Czechia | 400.1964354134203 | 500.0191538957788 | 307.7166062887306 | 11.45557648 | 14.3129902 | 8.808352114 |
| Bosnia and Herzegovina | 71.21196772654119 | 95.46303620135156 | 52.76668905047024 | 6.501781341 | 8.715947717 | 4.817694065 |
| Belarus | 344.704241549249 | 452.9274456104985 | 264.017428460003 | 11.98303303 | 15.74522123 | 9.178098742 |
| Brunei Darussalam | 7.044004502851169 | 9.786640611399656 | 5.134501924419731 | 11.76459689 | 16.34523114 | 8.575426857 |
| Latvia | 87.76692510201816 | 111.3669157020977 | 66.06329168989572 | 13.33804345 | 16.92456194 | 10.0397166 |
| Montenegro | 20.96686613463494 | 26.60058224866656 | 15.897635569603 | 12.0241346 | 15.25497322 | 9.117018663 |
| Republic of Korea | 2182.351119516629 | 3416.167505424384 | 1714.536644062531 | 13.04940587 | 20.42703206 | 10.2521012 |
| Austria | 444.8039993640595 | 534.6675658665426 | 367.4386785020015 | 15.0517066 | 18.09259661 | 12.43374428 |
| Romania | 426.7930060990026 | 536.1386484269913 | 342.2334524153439 | 7.106879628 | 8.927683406389999 | 5.698809297 |
| Republic of Moldova | 81.32369411190405 | 98.49224573267136 | 66.70312745964219 | 7.702325079 | 9.328391962 | 6.317582804 |
| Croatia | 178.0006859589581 | 215.2531821020879 | 143.7558517823742 | 11.93573034 | 14.43367436 | 9.639463313 |
| Slovakia | 147.2176259547786 | 194.2887060877079 | 114.3767055319274 | 8.975170354320001 | 11.84487403 | 6.973012980320001 |
| Ukraine | 989.820233711458 | 1409.518953196662 | 692.3139157003355 | 7.289844861 | 10.38084912 | 5.098765279 |
| Cyprus | 30.58297932931139 | 40.92768236447351 | 23.41898870186464 | 8.750453337809999 | 11.71029712 | 6.700680324 |
| New Zealand | 135.7171661165943 | 161.9808104900117 | 110.3957173222534 | 9.514512785 | 11.35573735 | 7.739341262979999 |
| Greece | 287.2794068091192 | 329.0201569188964 | 249.7739435092128 | 7.816170463 | 8.951834247 | 6.795738481 |
| Andorra | 2.210573783131397 | 3.165567871131326 | 1.454434365212072 | 8.353841957 | 11.96280075 | 5.49636249 |
| North Macedonia | 44.43132679037642 | 55.64043081646693 | 33.97933689238776 | 7.493825876 | 9.384363023 | 5.730984249960001 |
| Hungary | 278.414906085858 | 343.1046823712015 | 221.3328294290172 | 8.754845164 | 10.78903573 | 6.959881130839999 |
| Poland | 962.9087981658904 | 1083.04614227343 | 854.696237991375 | 7.947839888 | 8.939452362 | 7.054654465 |
| Finland | 190.0807274643741 | 229.7176046804832 | 154.1288712056362 | 9.444221875 | 11.41359283 | 7.657942372430001 |
| Estonia | 55.55283468011025 | 71.13361539463152 | 41.86941035862048 | 12.69850668 | 16.26002877 | 9.570690501 |
| Australia | 998.0659639413932 | 1203.443763947487 | 813.1623043982503 | 13.47312806 | 16.24557146 | 10.97706991 |
| Japan | 7080.832942911077 | 7839.380444408867 | 6120.970440687875 | 13.56457787 | 15.0177087 | 11.72579284 |
| Lithuania | 110.9389758444408 | 134.8323115809049 | 88.36220264075658 | 11.46324085 | 13.93212124 | 9.13039987 |
| Serbia | 209.6828779083732 | 278.3860442432676 | 150.3870029981767 | 7.474739724709999 | 9.923858564 | 5.360970417 |
| Singapore | 172.1906099583308 | 215.8710893329837 | 139.9864398727532 | 11.33707605 | 14.21301055 | 9.216744834 |
| Netherlands | 384.8318871220004 | 454.511458172187 | 320.1007537756434 | 6.577341388 | 7.768267457 | 5.470991377450001 |
| Ireland | 105.7365543598711 | 127.8507644905426 | 87.23323907848274 | 8.056926768 | 9.741988029 | 6.647008910829999 |
| Belgium | 364.5801400956547 | 440.3046635632498 | 294.8155756441139 | 9.643005865 | 11.64589067 | 7.797759703 |
| Russian Federation | 3818.802526485612 | 4188.275552501465 | 3469.053191994936 | 8.984313909 | 9.853555412 | 8.161475392 |
| Iceland | 17.87340615251139 | 21.80097045113352 | 14.83687779807925 | 18.35312326 | 22.38610226 | 15.23509535 |
| Germany | 3094.972754990837 | 3736.561796837146 | 2548.854793973131 | 9.820976068 | 11.85686818 | 8.088033051 |
| Slovenia | 63.03181314917907 | 83.61727440225685 | 46.41564986555092 | 8.608683094169999 | 11.42017944 | 6.339300749 |
| Italy | 3332.196817841144 | 3727.941967495484 | 2898.63481292933 | 14.668429 | 16.41051086 | 12.75987622 |
| Norway | 163.8908838306629 | 183.9478400424211 | 143.9274945952228 | 10.10589679 | 11.34265582 | 8.874907331 |
| Malta | 14.6898414264305 | 17.84479997570908 | 11.93201868715137 | 9.308963075 | 11.30826258 | 7.561328822 |
| France | 3530.209005958731 | 4206.387683462888 | 2864.534740848834 | 15.96648342 | 19.02471471 | 12.95576164 |
| Denmark | 107.0281158180247 | 129.3255653528215 | 88.50760193338004 | 5.559259044 | 6.717434136820001 | 4.597265707 |
| Israel | 210.6624965480491 | 256.156827470106 | 175.3648031903833 | 10.540796 | 12.8171692 | 8.774625983049999 |
| United States of America | 16883.55702693117 | 17632.52348304276 | 15780.67165801382 | 16.84182984 | 17.58894525 | 15.74167022 |
| United Kingdom | 1655.009939673739 | 1733.397639177218 | 1535.454579351342 | 7.869687499 | 8.242426468 | 7.301193436319999 |
| Portugal | 425.5869254962778 | 522.6308097449801 | 338.6592247642213 | 10.93872789 | 13.43301655 | 8.704452333 |
| Luxembourg | 19.85321867242699 | 23.47393485483521 | 17.20193929751179 | 11.17219783 | 13.2097192 | 9.680217197 |
| Chile | 382.7540940089776 | 461.9484021019487 | 323.5132232860007 | 8.548039617 | 10.31668453 | 7.225014422209999 |
| Antigua and Barbuda | 1.844180823570347 | 2.047931084237032 | 1.659633986922343 | 9.774312505 | 10.85420592 | 8.796198846 |
| Sweden | 244.35 | 294.6563993399549 | 197.0322072974272 | 7.258365091139999 | 8.752678405500001 | 5.852781578 |
| Barbados | 7.351604160622302 | 9.291258338151875 | 5.781127897560783 | 8.068148112 | 10.19685592 | 6.344601139 |
| Dominican Republic | 71.22537519885198 | 95.41617973167531 | 53.20557917921014 | 4.262379496 | 5.710043182 | 3.184010883 |
| Spain | 1276.897884230953 | 1505.464676462501 | 1060.506022071968 | 8.251317774 | 9.728317038 | 6.852992942999999 |
| Bolivia (Plurinational State of) | 206.035071890878 | 287.6344144401112 | 127.2224386896106 | 13.33153316 | 18.61143202 | 8.231948786390001 |
| Cuba | 325.0093528130077 | 389.1740706149664 | 265.1802941686086 | 9.511409464 | 11.38919205 | 7.760510084 |
| Argentina | 626.9319662858952 | 747.8812031968313 | 518.6159987114162 | 6.717145223519999 | 8.013033186960001 | 5.55661406 |
| Haiti | 54.51741016910273 | 75.727243561551 | 37.54410879793296 | 4.600282540419999 | 6.390008536900001 | 3.168043157 |
| Uruguay | 85.44739820036601 | 104.8780114920241 | 70.399559602813 | 9.449090676 | 11.59780007 | 7.785044790400001 |
| Suriname | 4.548748332340463 | 5.970766211084239 | 3.316378428177864 | 4.072191998 | 5.345230074 | 2.968933146 |
| Colombia | 1119.536089387327 | 1400.033011215668 | 888.9814148512869 | 11.70633893 | 14.6393324 | 9.295562551 |
| Peru | 657.074405588399 | 945.2147000036676 | 436.6225063467231 | 11.74722652 | 16.89862077 | 7.805970593 |
| Saint Lucia | 3.375736152317384 | 4.138438454965513 | 2.684969854300871 | 8.021829477 | 9.834254245 | 6.380347678 |
| Bahamas | 5.350665957962126 | 6.6496286184969 | 4.30040387354564 | 7.435756932 | 9.240909914 | 5.976220187 |
| Guyana | 5.690304854969919 | 7.60736958510323 | 4.332627980219916 | 5.052774048 | 6.755054534 | 3.847208678 |
| El Salvador | 97.0982141214533 | 124.0000277928814 | 74.37083150580627 | 9.495383893 | 12.12615368 | 7.272838146130001 |
| Paraguay | 82.25348919182952 | 106.5287830186912 | 58.79743538186288 | 8.307550746 | 10.75934018 | 5.938504044 |
| Egypt | 807.8912441155211 | 1064.766476750589 | 595.3951499386594 | 7.300762398320001 | 9.622095936 | 5.380474853 |
| Algeria | 425.4215408625948 | 604.6701327194376 | 319.3433440449078 | 7.004702914349999 | 9.956088806 | 5.258091182159999 |
| Brazil | 2126.24758103731 | 2266.18579211476 | 1942.737771745432 | 4.909623906 | 5.232749017 | 4.485889551 |
| Guatemala | 121.167506426581 | 142.9833850881014 | 101.4879963053755 | 6.618379794 | 7.810001003 | 5.543450747 |
| Nicaragua | 49.36933802478507 | 63.44935820163138 | 35.53282928779875 | 6.064779404 | 7.794440359 | 4.365032626 |
| Ecuador | 373.1450083090802 | 472.9695335658416 | 287.7888678654865 | 13.48301257 | 17.09001602 | 10.39880164 |
| Bahrain | 30.04189493885197 | 40.64190970512222 | 20.58881310177241 | 18.50185396 | 25.03006816 | 12.67999951 |
| Iraq | 435.8612574891726 | 610.1196385558271 | 300.5927097460365 | 11.21852574 | 15.70371937 | 7.736881849 |
| Libya | 105.1345432630606 | 149.7093690167627 | 72.96925378263182 | 12.57114316 | 17.90104234 | 8.725076526 |
| Belize | 1.910686405366954 | 2.248908628742698 | 1.637445835281211 | 3.81478163 | 4.490059331 | 3.269243071 |
| Mexico | 2059.624271029841 | 2325.266589030718 | 1802.614426311963 | 9.557224197 | 10.78987776 | 8.364627692 |
| Panama | 65.11698057083277 | 80.8068192417966 | 51.94204258313187 | 8.757406211 | 10.86749008 | 6.985544512780001 |
| Iran (Islamic Republic of) | 1107.519225476488 | 1422.383707230421 | 444.0841527218838 | 8.525319616 | 10.94904309 | 3.418414102 |
| Lebanon | 98.4221071447761 | 132.2186245887198 | 74.38899842367596 | 10.0427804 | 13.49130444 | 7.59049361 |
| Kuwait | 68.22877627573648 | 83.75860114647787 | 54.44733738293988 | 14.6343411 | 17.96532206 | 11.6783702 |
| Jordan | 139.920038151789 | 196.9897362616316 | 99.70216962933176 | 11.12912825 | 15.66840653 | 7.930231063310001 |
| Morocco | 310.8694701528542 | 419.8297928610569 | 217.0690032642561 | 5.174272107 | 6.987864025 | 3.613008664 |
| Oman | 24.85743846423455 | 32.80996258457255 | 17.2584343631056 | 7.958084 | 10.50407662 | 5.525270456319999 |
| Grenada | 1.714385245804228 | 2.01201508022109 | 1.42288468062255 | 8.534740254619999 | 10.01643367 | 7.083560238930001 |
| Trinidad and Tobago | 22.12009210385041 | 28.11698732257945 | 16.89269796951237 | 6.350166202 | 8.071735947 | 4.849502397939999 |
| Dominica | 0.749300029148332 | 0.9511353269146884 | 0.5876162221074863 | 4.967909938 | 6.306091632 | 3.895935348 |
| Qatar | 29.30281182422613 | 41.4181729096856 | 19.80681528750645 | 19.12914898 | 27.03816974 | 12.93007383 |
| Saudi Arabia | 482.7151261989674 | 668.4364972277531 | 354.9027671932149 | 16.0333063 | 22.20201217 | 11.78803908 |
| Syrian Arab Republic | 183.5566882462019 | 290.8524976589944 | 112.0047054906086 | 7.76892678 | 12.31015758 | 4.740532008 |
| Turkey | 2072.92625977561 | 2680.548839242753 | 1571.882430456267 | 12.54702659 | 16.22484998 | 9.514304026 |
| Switzerland | 192.1225541093886 | 225.6745009230888 | 160.4843651086898 | 6.555215979 | 7.700007432 | 5.475721886870001 |
| Canada | 1159.844206064728 | 1397.232073842679 | 955.6027424385936 | 9.451769957 | 11.38628453 | 7.787371136 |
| Yemen | 101.9575733862694 | 161.5317409719027 | 65.0182455607578 | 4.533612295 | 7.182617853640001 | 2.891080159 |
| Bhutan | 4.640437616727648 | 6.867724748857323 | 3.204811287391604 | 4.659536403 | 6.895990446 | 3.218001424 |
| Palestine | 54.19953982164528 | 72.54991440998344 | 32.10173244015599 | 12.59691389 | 16.86185949 | 7.460999867 |
| Central African Republic | 7.392919792391546 | 11.0746592101463 | 4.849866170440825 | 2.06680167 | 3.096087174 | 1.355852867 |
| Jamaica | 38.11996343957028 | 49.51140145243074 | 28.87502966586145 | 7.207513804 | 9.361344483 | 5.459532385139999 |
| Honduras | 37.09527603814398 | 51.064187554523 | 24.71633948573699 | 3.527275482 | 4.85553623 | 2.350200553 |
| Costa Rica | 100.0030200865712 | 116.8134285316899 | 81.74445717664854 | 10.44458337 | 12.20030747 | 8.537610135 |
| Tunisia | 192.0046540336029 | 282.7093895719459 | 128.6863509840172 | 8.215388002 | 12.09641161 | 5.506159781 |
| Gabon | 6.301749702923804 | 9.260339146219737 | 4.168569835464358 | 3.5254154 | 5.180552033180001 | 2.332041256 |
| Eritrea | 23.97095891401621 | 32.9586625134513 | 17.16369501933699 | 5.440414477 | 7.480250803 | 3.89544762 |
| Saint Vincent and the Grenadines | 2.468663182189933 | 2.867964430132421 | 2.130220270261571 | 9.709333059 | 11.27979793 | 8.378226014 |
| United Arab Emirates | 88.95415780725978 | 118.0780665468921 | 67.24043786198973 | 12.38779853 | 16.44360798 | 9.363935515 |
| India | 10306.13882186446 | 11871.09577911148 | 8479.267156334508 | 5.126275171180001 | 5.904685023 | 4.2175889 |
| Nepal | 170.4522108747168 | 243.1068518178165 | 121.8987060649307 | 4.306680722 | 6.142387868 | 3.079917854 |
| Bangladesh | 930.621643588708 | 1548.335062182529 | 609.927384599401 | 3.982777469 | 6.626402946 | 2.61030362 |
| Democratic Republic of the Congo | 134.4644436004513 | 222.4078380685021 | 78.88953658756054 | 2.226300841 | 3.682361996 | 1.306158245 |
| Angola | 46.885815511173 | 75.4083096964772 | 27.92893702462983 | 2.424756008 | 3.899830898 | 1.444378371 |
| Equatorial Guinea | 2.774277887146696 | 4.549316319707305 | 1.53395425326448 | 3.507440169 | 5.751570481 | 1.939334481 |
| Venezuela (Bolivarian Republic of) | 380.1732754024695 | 497.3684818902508 | 286.6119232324068 | 7.26604662 | 9.505935347 | 5.477858994 |
| Burundi | 31.30998015801424 | 46.11717579068068 | 20.61181342676808 | 3.911164828670001 | 5.76084287 | 2.574776455 |
| Djibouti | 4.921184962061039 | 7.678967540678356 | 3.128412597563369 | 4.745420643 | 7.404706664110001 | 3.016678673 |
| Ethiopia | 717.0274980699324 | 1085.431627097246 | 492.7521638503736 | 10.47431794 | 15.85595531 | 7.198110038 |
| Comoros | 4.009765432077701 | 6.223564400069256 | 2.707865461628258 | 4.960705765 | 7.699520663 | 3.350052274 |
| Kenya | 69.7589931377066 | 99.72450543879695 | 52.23761405218179 | 1.864828576 | 2.665880041 | 1.396439241 |
| Madagascar | 69.96064536085245 | 96.43512989716774 | 48.05144342207053 | 3.78845431 | 5.22207995 | 2.602044292 |
| Malawi | 56.80840748521295 | 81.8983310845203 | 38.21802043549836 | 4.859041208 | 7.005078706 | 3.268934026 |
| Rwanda | 55.64141403540337 | 78.24524676850612 | 39.37557217238127 | 5.261768127 | 7.399314928 | 3.723577738 |
| Seychelles | 0.6956629518135089 | 0.863818088815417 | 0.5378578198812096 | 3.353731851 | 4.164393447 | 2.592966748 |
| Eswatini | 4.693032084831443 | 7.137433219181954 | 2.957760248962596 | 5.18341447 | 7.883234966 | 3.266821321 |
| Afghanistan | 101.941387509307 | 169.8387042639722 | 49.57071242849155 | 8.296340396 | 13.82205734 | 4.034234907 |
| Pakistan | 1304.414607025016 | 1707.955843840931 | 985.4590261386124 | 6.579313635 | 8.614728102 | 4.970539254 |
| Congo | 13.73838793367528 | 20.31545139795558 | 8.899597141330897 | 3.16167749 | 4.675286918629999 | 2.048104631 |
| Uganda | 200.8083997036798 | 264.6114577227113 | 142.6160619371798 | 8.467596594 | 11.15801471 | 6.013768757 |
| Lesotho | 7.937236291396829 | 11.54490761947085 | 5.407882808681488 | 4.396034613689999 | 6.394141694 | 2.995153368 |
| Somalia | 41.60069444224518 | 58.32095162083041 | 26.61449261186469 | 4.411242132 | 6.184219817 | 2.822139695 |
| Mauritius | 15.71723745840743 | 17.65657931204598 | 13.61861942124645 | 4.645507948 | 5.218714786530001 | 4.025224211879999 |
| Cameroon | 18.18587517279942 | 26.60271145055157 | 11.94981074090268 | 0.893444954 | 1.306951581 | 0.587076399 |
| Mozambique | 87.82302396147759 | 142.4562717308082 | 55.67247833551417 | 4.908688164 | 7.962301723 | 3.111699224 |
| United Republic of Tanzania | 209.7194713994864 | 293.1856565588524 | 148.7000320082556 | 5.087584862 | 7.11239113 | 3.607314222 |
| Namibia | 8.125217443560599 | 11.11579944438406 | 5.481051201761476 | 3.596073782 | 4.919651102 | 2.42581379 |
| Guinea | 15.30623133960869 | 20.48554733242954 | 10.9782668540219 | 1.675452138 | 2.242390914 | 1.201704081 |
| Guinea-Bissau | 1.004968981776754 | 1.35259630306408 | 0.7092580437044425 | 0.874872532 | 1.177498385 | 0.617442321 |
| Togo | 4.899964008101327 | 7.336550479998591 | 3.166373293045702 | 0.7843863547600001 | 1.174435175 | 0.506873112 |
| Botswana | 7.975824988443777 | 12.11317001632005 | 5.203984839185286 | 3.290038732 | 4.996699223 | 2.146650874 |
| Burkina Faso | 10.94761552948796 | 14.67913553194865 | 7.214572661379773 | 0.7281835024600001 | 0.976386529 | 0.479879182 |
| Cote d'Ivoire | 36.09854766016463 | 49.73740184973019 | 25.72238075246376 | 2.013099368 | 2.773694199 | 1.434454065 |
| Ghana | 8.176656235203323 | 10.96394126734956 | 5.011183175197498 | 0.299821904 | 0.402026165 | 0.183750232 |
| Niger | 7.836044243613544 | 11.43966053016776 | 5.023984145567615 | 0.562847411 | 0.821687973 | 0.360862749 |
| Sierra Leone | 3.987830503363557 | 5.69301611297662 | 2.604151056381709 | 0.660145451 | 0.942421872 | 0.431091159 |
| Senegal | 9.603136710443376 | 14.31192886391889 | 6.567136632380025 | 0.74375725 | 1.108450413 | 0.508620842 |
| Cook Islands | 0.2772408367863633 | 0.3824683586294435 | 0.2121171473533523 | 5.882551728 | 8.11529041 | 4.500744213 |
| Tokelau | 0.0134633847029227 | 0.0181333030980919 | 0.0086090983325166 | 5.326950001 | 7.174659351 | 3.406293245 |
| Tuvalu | 0.0865424998565038 | 0.1174395522848568 | 0.0558109670326623 | 4.74905083 | 6.444537703 | 3.062646905 |
| Gambia | 1.980088438214422 | 2.713515228488495 | 1.404925831467166 | 1.280448072 | 1.754727353 | 0.908512235 |
| South Africa | 303.1217825833204 | 341.5590125571071 | 238.2094471274001 | 3.842552636 | 4.329805905070001 | 3.019685129 |
| Liberia | 2.712496134155704 | 3.778649411845477 | 1.791133065470642 | 0.8216656699200001 | 1.144623383 | 0.5425676120299999 |
| Zimbabwe | 98.41388112356478 | 131.5922139697841 | 69.71047960154598 | 8.887964139 | 11.88436901 | 6.295699709340001 |
| Guam | 1.581569517722777 | 1.958724647370274 | 1.169790455484007 | 4.196766653 | 5.197564945 | 3.104092182 |
| Chad | 5.782388822261372 | 7.792991032889812 | 4.093637302552423 | 0.622751732 | 0.839289575 | 0.440876565 |
| American Samoa | 0.9154411941276432 | 1.257038437515175 | 0.5680207156905082 | 10.83848452 | 14.88286931 | 6.725154795 |
| Cabo Verde | 0.6649668280195781 | 1.115952612953162 | 0.230598466004744 | 0.8675797896799999 | 1.455979295 | 0.300860976 |
| Mali | 42.61241663076616 | 60.6898782538348 | 30.51029291309682 | 2.944121504 | 4.193105901 | 2.107977358 |
| Mauritania | 3.299521506237959 | 4.835259952894042 | 2.203091260146425 | 0.919707413 | 1.347778583 | 0.614088849 |
| Sao Tome and Principe | 0.0546810430093834 | 0.080443033017283 | 0.0395575612974852 | 0.299067172 | 0.439967291 | 0.216352274 |
| Nigeria | 31.25550831412876 | 58.01620340548875 | 20.33895661291384 | 0.208349048 | 0.386735696 | 0.135579374 |
| Bermuda | 2.802135914774012 | 3.584141517455391 | 2.237441543398897 | 12.03105317 | 15.38861728 | 9.606521237 |
| Greenland | 0.8062220324691474 | 1.166954044308491 | 0.4913019342041421 | 5.938584798 | 8.595715905 | 3.618901593 |
| Monaco | 1.714622221267712 | 2.195441387492769 | 1.282343921527582 | 10.95930552 | 14.03254467 | 8.196323739 |
| Niue | 0.0232190168655148 | 0.030554140788282 | 0.0152708586909672 | 5.93437142 | 7.809099795 | 3.902962296 |
| Palau | 0.2406812437719253 | 0.3204939019992653 | 0.1644214809974609 | 5.747822226 | 7.653865936 | 3.926626887 |
| Puerto Rico | 73.64953949653051 | 88.6656782692724 | 59.81016913235248 | 6.309045338440001 | 7.59537382 | 5.123522446 |
| Saint Kitts and Nevis | 0.706382010094082 | 0.8476257402640571 | 0.5692875586737465 | 5.48460291 | 6.581269817 | 4.420152491 |
| San Marino | 1.36237260939309 | 1.954922630897653 | 0.8453887352632067 | 11.51917543 | 16.52932287 | 7.147957233 |
| United States Virgin Islands | 0.717464181093331 | 0.9754953209884132 | 0.5108265845563192 | 2.228893851 | 3.030500448 | 1.586947841 |
| Zambia | 77.82441426835592 | 127.7013760813571 | 43.51276200810898 | 7.204907488 | 11.8224674 | 4.028368575 |
| Benin | 5.397592053382059 | 7.602802937324205 | 3.719157156089722 | 0.652622525 | 0.919254437 | 0.449683064 |
| South Sudan | 29.70044768558989 | 42.24781577448504 | 20.11483821023817 | 4.769451661 | 6.784372992200001 | 3.230144863 |
| Nauru | 0.0623465454008774 | 0.0967033296108084 | 0.0377260663330421 | 6.745433311 | 10.46258227 | 4.081680274 |
| Northern Mariana Islands | 0.5155507108479223 | 0.6629816551294933 | 0.3624934566056282 | 5.303831226 | 6.820556601959999 | 3.729224058 |
| Sudan | 150.6578028508606 | 228.8783397221565 | 99.70237565577364 | 4.838335668 | 7.350367613 | 3.201915541 |

Table S4. The deaths cases and rates for thyroid cancer in middle-aged and elderly patients in 2021 in various countries.

| location | Number | | | Rate | | |
| --- | --- | --- | --- | --- | --- | --- |
|  | val | upper | lower | val | upper | lower |
| Tajikistan | 0.1837001947391486 | 0.2608518026164029 | 0.1216735408299541 | 0.01788454428558849 | 0.02539581203217106 | 0.01184580034031898 |
| New Zealand | 25.64588673062273 | 30.50026680296038 | 20.69799632256441 | 1.797916388555047 | 2.138234880171457 | 1.451042312924034 |
| Antigua and Barbuda | 0.6826909636570219 | 0.7276994831613243 | 0.633478624361935 | 3.618319168103626 | 3.856868083381496 | 3.357489656570662 |
| Chad | 3.80445178548435 | 4.968961419427942 | 2.78165866745067 | 0.4097318616770699 | 0.5351472242996362 | 0.2995790848797991 |
| Micronesia (Federated States of) | 0.3139230706886557 | 0.4161123189053869 | 0.2051854839284771 | 2.385585172013188 | 3.162148534337034 | 1.559259237934734 |
| Indonesia | 1432.854393770793 | 1880.434066866152 | 946.8510455002202 | 3.42137005871521 | 4.490100907484873 | 2.260891149317896 |
| Democratic Republic of the Congo | 82.57738972327867 | 136.7407538375337 | 48.15461333606638 | 1.367217290168848 | 2.263989253521971 | 0.7972862810884619 |
| Nauru | 0.03052308501535238 | 0.04503838899714853 | 0.01886055921523054 | 3.302371175246228 | 4.872819294933676 | 2.040572473918487 |
| Estonia | 14.5476128967445 | 18.66414665211182 | 11.08314811870952 | 3.325356134005738 | 4.266331184099062 | 2.533433824658098 |
| Portugal | 89.80648469371955 | 109.3169961160509 | 72.80635462673187 | 2.308268040758076 | 2.809740625155567 | 1.871319004659105 |
| United Arab Emirates | 16.56371889798523 | 21.74509676956467 | 12.76280806356383 | 2.306671410457195 | 3.028232569322411 | 1.777354750988695 |
| Cote d'Ivoire | 19.64908396494947 | 26.09896237097841 | 14.47255401304898 | 1.095765926416253 | 1.455454805524399 | 0.8070875763983062 |
| Uganda | 125.7079889118022 | 162.4057111896563 | 92.1789155896176 | 5.3007968806018 | 6.848249619760034 | 3.886958278821271 |
| Bosnia and Herzegovina | 21.94439506382505 | 28.14425992433597 | 16.32262001738195 | 2.003562925181883 | 2.569621790761401 | 1.490284704296501 |
| Yemen | 34.41974407314878 | 53.44491380458508 | 23.11342696838041 | 1.530497144462612 | 2.376464153543164 | 1.02775412561675 |
| Cyprus | 8.103798741202336 | 10.32975558801261 | 6.265868745926444 | 2.318672487082804 | 2.955566993345782 | 1.792800874358356 |
| Iran (Islamic Republic of) | 178.7357325771952 | 216.6798559849157 | 81.89062885422182 | 1.375849025476977 | 1.667930437852328 | 0.6303671922799012 |
| Dominica | 0.4091135342009169 | 0.4970635933262922 | 0.3236849063600713 | 2.712450438340707 | 3.295565286625237 | 2.146052850232689 |
| Burundi | 21.32417295197972 | 31.03044838791039 | 14.26992723415835 | 2.663762635085585 | 3.876246415361849 | 1.782563809501097 |
| United Kingdom | 437.459454483612 | 462.5150822328048 | 389.7873852993689 | 2.080150165744814 | 2.199291420279701 | 1.853466157435844 |
| Iraq | 90.8345498851146 | 122.976414279359 | 65.95414545456596 | 2.337968145928067 | 3.165259690825097 | 1.697577533655513 |
| Saint Kitts and Nevis | 0.3077029971234187 | 0.365876041751823 | 0.250000790253221 | 2.389116270611344 | 2.840792623237108 | 1.941095670966433 |
| Vanuatu | 0.5866907975699776 | 0.7892058791716081 | 0.3976148945787226 | 2.046021767658279 | 2.752271579232337 | 1.386639662361818 |
| Bangladesh | 481.6771711712441 | 741.9443213416091 | 319.9141078864405 | 2.061431730029827 | 3.175295939830389 | 1.369135039714882 |
| Guatemala | 62.78300874513126 | 72.05755182799965 | 54.28948721331984 | 3.429317056399069 | 3.93590871901758 | 2.965386148338018 |
| Guinea-Bissau | 0.6248864329768591 | 0.8030797407671637 | 0.4671354667657697 | 0.5439928851767044 | 0.6991184992218701 | 0.4066632861969788 |
| Philippines | 791.7784609697748 | 945.7200072121274 | 635.6360131736591 | 5.677836989622831 | 6.781750582351815 | 4.558140749513974 |
| Ukraine | 280.0409356316051 | 377.550498750667 | 202.6801896917106 | 2.062450236821471 | 2.780590322640307 | 1.492702501817783 |
| Germany | 808.0266847055411 | 949.3445552618616 | 672.5572773116405 | 2.564032500846973 | 3.012462756821666 | 2.134160604283283 |
| Namibia | 4.327278603660126 | 5.783038713483418 | 3.031725496100475 | 1.915174977358024 | 2.559467982437787 | 1.341786684000162 |
| Haiti | 36.51473518354301 | 49.67171979726268 | 25.8134142112679 | 3.081182657282076 | 4.191393990053592 | 2.178184883258957 |
| Greece | 92.68041657112144 | 102.3978090987412 | 80.26310831568844 | 2.521607596374464 | 2.785994094851133 | 2.183762990341692 |
| China | 6489.260513189762 | 7903.481865772088 | 5154.558580113463 | 1.712408695705903 | 2.085598358363559 | 1.360202894793792 |
| North Macedonia | 13.8638533897614 | 17.19882195694824 | 10.96437619587248 | 2.338289463156879 | 2.900768136392952 | 1.84926114032846 |
| Kenya | 41.2540885486311 | 58.34710790753382 | 30.55191071744929 | 1.102822729317955 | 1.559760960772236 | 0.8167273293064662 |
| Libya | 20.63556124765284 | 27.86330638890374 | 15.65556637881307 | 2.467434456406731 | 3.331670092628028 | 1.871966720655099 |
| Azerbaijan | 26.75913739381236 | 34.43436001371919 | 19.82899915966733 | 1.396761109820822 | 1.797388839591434 | 1.03502495111436 |
| Slovenia | 13.78907794698118 | 17.90613866698535 | 10.08236869768768 | 1.883268087584408 | 2.445563050194916 | 1.377017614130766 |
| Guam | 0.5137863078833335 | 0.613769006676571 | 0.3752865294801207 | 1.363355337582179 | 1.628663976551561 | 0.9958398759150956 |
| Nigeria | 17.23064865592622 | 31.89311510772584 | 11.79321950694942 | 0.1148594099960521 | 0.2125993314215513 | 0.07861353693473766 |
| Georgia | 46.15658058659463 | 57.5623165883061 | 36.15564407110694 | 4.396415270274707 | 5.482811863986189 | 3.443825857130268 |
| Venezuela (Bolivarian Republic of) | 165.2801408819769 | 212.4757554909677 | 128.1727455218768 | 3.158910125116293 | 4.060934433988864 | 2.44969650577761 |
| Viet Nam | 859.5130548521166 | 1190.665365710603 | 640.8147053855638 | 4.920333909831502 | 6.816035127210248 | 3.668382122910013 |
| Democratic People's Republic of Korea | 115.548487094331 | 156.5213248150378 | 83.14480982933159 | 2.050270015556023 | 2.777284126631558 | 1.475305430896895 |
| Italy | 610.9359677967105 | 679.0104049197306 | 517.2846183997784 | 2.689358209404823 | 2.989023896117712 | 2.277102197975672 |
| Seychelles | 0.2640086597989504 | 0.3163149985424585 | 0.2029389784171182 | 1.272763267146982 | 1.524927672066053 | 0.9783515336138568 |
| Trinidad and Tobago | 9.431630994647373 | 11.91100012311575 | 7.252425710622342 | 2.707602847551235 | 3.419372308865134 | 2.082003474995898 |
| Japan | 2233.779153769622 | 2510.682585844207 | 1756.738235171938 | 4.279195897758831 | 4.809653006112304 | 3.36534031875881 |
| Turkmenistan | 13.4172813048075 | 17.23676319589288 | 10.42599284286343 | 1.89589678741323 | 2.435599524680663 | 1.473219938326521 |
| Serbia | 71.00253467724674 | 93.7257881131692 | 52.39266886770934 | 2.531086332851684 | 3.341121023458572 | 1.867684987803385 |
| Benin | 3.256254284729001 | 4.564720272038763 | 2.360701661031947 | 0.3937135061041416 | 0.551920048479445 | 0.2854322932301676 |
| Bahamas | 2.214631143455586 | 2.693989426315418 | 1.819109721224042 | 3.077646596954393 | 3.743805109321149 | 2.527995174074958 |
| Taiwan (Province of China) | 191.9852139750294 | 213.2508603691327 | 163.7941762947014 | 2.549626409999973 | 2.832041146788912 | 2.175240212714917 |
| Rwanda | 33.35806702392161 | 44.88664586380565 | 24.27414822745645 | 3.15452827491732 | 4.244736166578077 | 2.295501321408505 |
| Latvia | 25.54667459141626 | 32.11975451486316 | 19.66147323892591 | 3.882358362378367 | 4.88127709507127 | 2.987977349954994 |
| Saudi Arabia | 73.47273375359028 | 92.89027561320074 | 58.09143423703696 | 2.440385190613032 | 3.085335761680987 | 1.929497768369186 |
| Algeria | 99.5870499964196 | 127.6140597773598 | 75.52883310435335 | 1.639732905689704 | 2.101206663447839 | 1.243606603208015 |
| Equatorial Guinea | 1.343163674998044 | 2.142124229442041 | 0.7891003737681574 | 1.698123410353701 | 2.708226383435145 | 0.9976370287236326 |
| Togo | 2.681668847796508 | 3.876524440257925 | 1.807619534630766 | 0.4292816128260362 | 0.620554124436675 | 0.2893637780219415 |
| Papua New Guinea | 13.26819271137433 | 21.58529389396946 | 7.429762911228938 | 1.615505334810114 | 2.628176888722611 | 0.904631239579079 |
| Niue | 0.01098890879110491 | 0.01407679050048751 | 0.0076130326822699 | 2.808571381047454 | 3.597779514620456 | 1.94575695556868 |
| Gambia | 1.158292528249672 | 1.522920454018035 | 0.8377782255560771 | 0.7490238346643696 | 0.9848148809879148 | 0.541759394798636 |
| Zambia | 46.99517966199828 | 73.43449169389514 | 26.97038532092193 | 4.350767365410887 | 6.798492787031312 | 2.496891662736002 |
| Spain | 341.1496882565706 | 410.2098846819967 | 277.0815633188423 | 2.204510259698433 | 2.650777446207311 | 1.790501853398378 |
| Northern Mariana Islands | 0.1606664519800381 | 0.1931424463355371 | 0.1200192616796855 | 1.65288831340461 | 1.986991611727695 | 1.234722199743661 |
| Colombia | 405.09462807191 | 495.1822478538721 | 332.0108514353717 | 4.235839345410598 | 5.177833284513361 | 3.471644722387125 |
| Lao People's Democratic Republic | 34.54110539226581 | 47.98008101361651 | 22.78914741361951 | 4.467587106518345 | 6.205799984447961 | 2.947575070264817 |
| Bulgaria | 58.90663818657303 | 74.63210521366848 | 46.90966271187363 | 2.478288792473199 | 3.139882288374663 | 1.973558412709305 |
| Dominican Republic | 36.47731706634857 | 47.37152563964219 | 27.34761086129727 | 2.18293224693583 | 2.834880392031907 | 1.636578192337823 |
| Comoros | 2.515326954361888 | 3.762542739627744 | 1.733033458156557 | 3.111852085603196 | 4.654852702618967 | 2.144032914620732 |
| Denmark | 33.09949345818828 | 39.28455496488645 | 27.64719914817687 | 1.719255327906882 | 2.040520061527511 | 1.436052019866996 |
| American Samoa | 0.3858008926792654 | 0.5048589108969319 | 0.2447856216318562 | 4.567739611511526 | 5.977342430479094 | 2.898171055259446 |
| Jordan | 23.41121553659141 | 31.69987337284926 | 17.43570003738044 | 1.862109414150816 | 2.521382648530593 | 1.386821676609235 |
| Djibouti | 2.865274297218938 | 4.3582137260259 | 1.88277382686973 | 2.76293858135494 | 4.202556404849339 | 1.815528953466258 |
| Argentina | 265.0272877831549 | 311.7210100378549 | 220.2507814454095 | 2.839585275547833 | 3.339876423995358 | 2.359835778238836 |
| South Africa | 151.4027797238735 | 168.6315862147945 | 116.7693455708024 | 1.919272001594367 | 2.137674635807682 | 1.480237919061138 |
| San Marino | 0.3771634532938302 | 0.5187570706967733 | 0.2507707842297667 | 3.189004207650247 | 4.386210982938277 | 2.120324965424108 |
| Bhutan | 2.62101063273216 | 3.723794168784948 | 1.89346587796601 | 2.631798003605115 | 3.739120298428064 | 1.901258871403666 |
| Liberia | 1.429859635616909 | 1.951818928264811 | 1.012500429214843 | 0.4331311520043041 | 0.5912423568334384 | 0.3067052641999141 |
| Paraguay | 38.33615710981778 | 49.36469380454097 | 27.39709473703106 | 3.871927789724958 | 4.985803069033127 | 2.767089360734888 |
| Cameroon | 10.3071278336729 | 14.88988540768822 | 7.08783229339322 | 0.5063738349415368 | 0.7315178871750069 | 0.3482146411440238 |
| Malta | 3.953140199586107 | 4.741061561622439 | 3.220910907031173 | 2.50510778699474 | 3.004414120674325 | 2.041093557791064 |
| Honduras | 20.66094604464569 | 28.16673325290846 | 14.09484250550785 | 1.964585688408226 | 2.678287863406742 | 1.340235137677286 |
| Jamaica | 16.58422991779145 | 21.15797096777833 | 12.66752181608166 | 3.135655317639876 | 4.000433213025506 | 2.395105611826038 |
| Sri Lanka | 131.890263419759 | 185.7022295893013 | 85.27910776477808 | 2.738412163253729 | 3.855699663228468 | 1.770633706532635 |
| Mexico | 878.6739028536289 | 978.1369725048958 | 782.3872592681712 | 4.077289049074706 | 4.538825101709719 | 3.630492488726031 |
| Montenegro | 5.461726997583709 | 6.751685186539284 | 4.397629891247266 | 3.132205839621486 | 3.871974519766374 | 2.521964578594369 |
| Saint Lucia | 1.534185984394917 | 1.828402764090823 | 1.254581984909388 | 3.645716903738162 | 4.344870134188131 | 2.981288315779694 |
| Kiribati | 0.01277883467876287 | 0.02491697389620131 | 0.00857692614588331 | 0.1016060501176058 | 0.1981178536321433 | 0.06819617044439212 |
| Madagascar | 41.74005417781683 | 56.4768075654486 | 29.08983600885508 | 2.260274863314503 | 3.058288040466331 | 1.575250114162533 |
| Netherlands | 128.6040369712686 | 147.1448495664982 | 105.3417902533053 | 2.198031616845555 | 2.51492129811915 | 1.800445701433209 |
| Republic of Korea | 476.3111397316319 | 641.3848086347723 | 370.9029616701911 | 2.848110611402794 | 3.835171439606651 | 2.217820606775623 |
| Morocco | 85.32129732488048 | 108.4034510204989 | 63.02650980401515 | 1.420131763658702 | 1.804323057796948 | 1.04904579901562 |
| Sao Tome and Principe | 0.03019412743640083 | 0.04407340483809054 | 0.0222039781626418 | 0.1651408203387945 | 0.2410507886149947 | 0.1214402759704465 |
| Bolivia (Plurinational State of) | 115.1070813136478 | 158.7744730402447 | 71.49334720770277 | 7.448022597938382 | 10.27352835015625 | 4.625988770875828 |
| Singapore | 34.26975068043718 | 41.52929054244901 | 27.95947518568644 | 2.256329597561166 | 2.734299653663234 | 1.840859362590978 |
| Iceland | 3.998542869480046 | 4.764212473536222 | 3.257761877308772 | 4.105862616185388 | 4.89208257337007 | 3.345199274108411 |
| Burkina Faso | 6.960790514029322 | 9.3671497089154 | 4.854143295236235 | 0.4629987966530107 | 0.6230584061616766 | 0.3228746074093085 |
| Canada | 251.2580656313155 | 300.6987681465002 | 208.6850262653614 | 2.047545199505353 | 2.450445989339826 | 1.700610178083907 |
| Kazakhstan | 80.80422801644649 | 95.28420164587278 | 66.52511135531938 | 2.546261577755782 | 3.00254711385447 | 2.096305343892372 |
| Slovakia | 41.67688219677569 | 53.5585413621811 | 33.50618268088486 | 2.540844651767296 | 3.265213859665431 | 2.042715303508253 |
| Andorra | 0.4952370174064759 | 0.6919325775028478 | 0.3458725055869692 | 1.871519424648682 | 2.614839387664229 | 1.307065283705623 |
| Tunisia | 37.30525490098842 | 52.15227999530705 | 26.02396108816059 | 1.596196431054021 | 2.231462656421455 | 1.11349872614622 |
| Somalia | 29.4591356744969 | 40.61177003141387 | 19.59192290434959 | 3.123779115034145 | 4.306378858172891 | 2.077482525902775 |
| Fiji | 7.495042008155486 | 10.08344223322705 | 4.871935293349302 | 5.481211015562122 | 7.37413807466439 | 3.562902698604607 |
| Bahrain | 4.039730266019657 | 5.325629030650561 | 2.808532545048539 | 2.487942240807082 | 3.279886663640804 | 1.729686462554941 |
| Austria | 90.95334358629454 | 108.3969137791374 | 75.55701881565017 | 3.077766935738623 | 3.668039282679119 | 2.556771253309162 |
| Uzbekistan | 39.49949564662059 | 52.00743859069883 | 29.88105095921894 | 0.8496980604457373 | 1.118764150677158 | 0.6427897528433587 |
| Luxembourg | 5.099575341714526 | 5.877951235824428 | 4.418265349421389 | 2.869734398310883 | 3.307757552880489 | 2.486334097347943 |
| Barbados | 3.062495398903728 | 3.715710448698427 | 2.42945977943542 | 3.360989782750619 | 4.077872201279253 | 2.666253637216551 |
| Samoa | 0.768261211756713 | 1.37520808887834 | 0.541262512002251 | 3.120282013491871 | 5.585388145164452 | 2.198330014496266 |
| Syrian Arab Republic | 33.92882436681251 | 51.08501337531364 | 22.81612593853774 | 1.436017149531035 | 2.162142563440208 | 0.9656788510965466 |
| Palau | 0.1011992523264451 | 0.1294583995413734 | 0.07175725674981384 | 2.416787044412214 | 3.091657058815766 | 1.713668870755017 |
| Gabon | 3.31248411060074 | 4.657369044140695 | 2.292722432095899 | 1.853117474760012 | 2.605492335641192 | 1.28262773852843 |
| Sweden | 87.97764663781223 | 104.8802103499692 | 70.54847762989293 | 2.613349140282232 | 3.115434636244409 | 2.095620994743236 |
| Ghana | 5.365519682703831 | 7.127140336176008 | 3.177028715634717 | 0.1967430551243181 | 0.2613382201465361 | 0.1164953951704969 |
| Lithuania | 28.13830328781827 | 34.62262693816255 | 22.5040570073713 | 2.907509693020442 | 3.577529973675383 | 2.325327266962229 |
| Solomon Islands | 1.114985674507972 | 1.696124231045798 | 0.7015770653391716 | 2.055435159222821 | 3.126742754286727 | 1.2933315646758 |
| Malaysia | 186.6963836930001 | 241.9882396720708 | 154.9963142608199 | 3.807962160405193 | 4.935725276015927 | 3.161390103184967 |
| Costa Rica | 33.20743948092063 | 38.57458103736167 | 28.25328012027616 | 3.468273956071879 | 4.02883260105415 | 2.950848278171448 |
| Botswana | 4.498668451162234 | 6.633200067992547 | 3.100158835338741 | 1.855706898006685 | 2.736204113653248 | 1.278819765917221 |
| Maldives | 0.7726179120943971 | 0.9905167183740438 | 0.5510869629804646 | 1.420347447239497 | 1.820923214913412 | 1.01309450483027 |
| Croatia | 35.04827683813109 | 40.85120565686346 | 29.98475303756623 | 1.807193926323084 | 2.173170526643153 | 1.482064387668091 |
| Kuwait | 8.42556758989483 | 10.13183748013868 | 6.909736409019535 | 2.260641591625746 | 2.726360018848309 | 1.801800570990147 |
| Finland | 45.49918499940375 | 54.87254562247893 | 36.26424366215237 | 2.690388199115787 | 3.50090734834421 | 1.763391956359202 |
| Tokelau | 0.00679971302831934 | 0.00884822692699097 | 0.00445681380242077 | 1.78694428085251 | 2.480483725221431 | 1.325956608115545 |
| Mali | 25.86374716438255 | 35.90184909620479 | 19.1915365412971 | 2.350141400012701 | 2.739253347547671 | 2.010609817087338 |
| Grenada | 0.7683241810732018 | 0.8832979771649552 | 0.6618977443188366 | 3.824955524348093 | 4.39733065889371 | 3.295131789487005 |
| Bermuda | 0.8237841210335243 | 1.033001008 | 0.6810662808309823 | 3.536941412608281 | 4.435220287142369 | 2.924178157719273 |
| Eswatini | 2.73435652599255 | 4.0112757736552 | 1.785259060187161 | 3.020073787458008 | 4.430420357814897 | 1.971803618233745 |
| Chile | 122.2649217132633 | 145.683035445736 | 103.4933182916098 | 2.73054008036023 | 3.253536351546769 | 2.311314232118495 |
| Eritrea | 15.35940553743899 | 21.0113868718948 | 11.22104311859583 | 3.48594866564931 | 4.768714248151544 | 2.546711862716049 |
| Mauritania | 1.646243541063418 | 2.325221291031836 | 1.160004614860986 | 0.4588733198869788 | 0.6481313284900905 | 0.3233392602176172 |
| India | 5202.504430159832 | 5947.238528969336 | 4379.307683955253 | 2.587726572411822 | 2.958157437534851 | 2.178268373370728 |
| Nicaragua | 20.37124866211473 | 25.86879555112037 | 14.91682330403609 | 2.502507310412326 | 3.177853800323704 | 1.832458087652838 |
| Palestine | 11.11076900258935 | 14.20904385154298 | 6.627697384079018 | 2.582335586069014 | 3.302428443369079 | 1.540391921082613 |
| Thailand | 595.8466927779214 | 830.0835859036903 | 437.7902622933129 | 3.065372511319853 | 4.270419618281503 | 2.252240806273311 |
| Nepal | 95.57968276422348 | 131.2686802460273 | 70.62241800359756 | 2.414935981566354 | 3.316661554117631 | 1.784360581765242 |
| South Sudan | 18.61292626445634 | 25.75639035616493 | 13.14382467494072 | 2.988960066678735 | 4.136094515314248 | 2.110703417540885 |
| United States of America | 2286.073419725582 | 2429.721162516695 | 2026.962739759263 | 2.280423460136612 | 2.423716181984468 | 2.02195316418344 |
| Oman | 3.786239269028743 | 4.807324700821976 | 2.682767859685615 | 1.212160705518996 | 1.539060182665656 | 0.8588854402681204 |
| Poland | 320.0634948513707 | 351.0575748870133 | 285.8980882030588 | 2.641800984615634 | 2.897625820851455 | 2.359800049253676 |
| Saint Vincent and the Grenadines | 1.200663392134589 | 1.368072041769283 | 1.050561278093993 | 4.722248401687712 | 5.380672097575265 | 4.131891876485446 |
| Malawi | 35.34243476016015 | 48.72852306137182 | 24.74262276127581 | 3.022974142341718 | 4.167937670640603 | 2.116331524090889 |
| Brazil | 994.3934962881676 | 1061.888178007992 | 891.7095052078089 | 2.296109881443605 | 2.45195885493374 | 2.059006835752169 |
| Ireland | 24.27311846543524 | 28.98947861601416 | 19.63433293650928 | 1.849566019073804 | 2.208943800739821 | 1.496099277818853 |
| Kyrgyzstan | 19.22854557205838 | 24.35382531540917 | 15.01889285572303 | 2.273548548833182 | 2.879552381998389 | 1.775806803964884 |
| Senegal | 5.545441991960159 | 8.158850420191479 | 3.827419738453397 | 0.4294911974921327 | 0.6318981322332086 | 0.2964313916107383 |
| Angola | 27.45149449819955 | 43.21750903794241 | 17.00749441084225 | 1.419686860212536 | 2.235045152689497 | 0.8795629083798873 |
| Belarus | 75.12982974634265 | 95.2753645069196 | 58.92838856827085 | 2.611755594608124 | 3.312079464566994 | 2.048541159271233 |
| Cabo Verde | 0.2793750851857325 | 0.4550480432564402 | 0.1110428928619405 | 0.3644996523655756 | 0.5936995185749608 | 0.144877256391481 |
| Marshall Islands | 0.1294107524970503 | 0.1789034368992199 | 0.08561692618810197 | 2.208754574042917 | 3.053484945713124 | 1.461291072685596 |
| Monaco | 0.4747415026526342 | 0.5983441857308468 | 0.3647126362439263 | 3.034392710645748 | 3.824420712101549 | 2.331124114313462 |
| Ecuador | 174.2052281956038 | 214.4987654205666 | 136.8995427312477 | 6.294634069736349 | 7.750578158404295 | 4.94665134182916 |
| Congo | 7.621116341028908 | 11.05844824796902 | 5.1250858195447 | 1.75388204950921 | 2.544930822420823 | 1.179459231281083 |
| Norway | 41.4240733794996 | 46.02643490534346 | 35.19504788573742 | 2.554305649048512 | 2.838098069381668 | 2.170209308231915 |
| Belize | 0.8340702497388922 | 0.9517030730474187 | 0.7293812202023611 | 1.665263257046389 | 1.900123112723585 | 1.456246337479235 |
| Cambodia | 124.2654452896957 | 169.0332162691941 | 79.22360810850257 | 5.716478926291368 | 7.775893100398557 | 3.644457114857935 |
| United Republic of Tanzania | 127.6229303771114 | 175.9014692633887 | 92.61606984582218 | 3.096004792970351 | 4.267193915080708 | 2.24677332906758 |
| Peru | 252.0953645812758 | 339.7385380639171 | 169.167954780789 | 4.506980226495113 | 6.073871591315088 | 3.024397645787829 |
| Turkey | 410.3484536686696 | 520.5799283940304 | 325.6521195591582 | 2.483760788429939 | 3.150970843995842 | 1.971110060239737 |
| Egypt | 193.2751024378725 | 243.2886610751141 | 150.3885483963558 | 1.746591030274869 | 2.198554225772394 | 1.359033245213601 |
| Belgium | 99.2519226659696 | 120.4673868540343 | 79.58031420845425 | 2.625175556994545 | 3.186316505409607 | 2.104869004715102 |
| Albania | 16.77129549955773 | 23.15098771035505 | 12.00094208104182 | 2.132954649438263 | 2.944316786809464 | 1.52626642408587 |
| Republic of Moldova | 21.83957682134118 | 26.03957559895435 | 18.29554909220914 | 2.06846875455902 | 2.466258799290546 | 1.732807002366231 |
| Cuba | 112.017501639016 | 130.4668796209268 | 93.65280632231882 | 3.278195892076871 | 3.818117549199491 | 2.74075247595384 |
| Puerto Rico | 24.80712262744351 | 29.46036050914132 | 20.38706113715252 | 2.125054174712081 | 2.523664796948898 | 1.746418157004976 |
| Russian Federation | 1022.565126534333 | 1113.085377073898 | 935.7775732461986 | 2.40574002607338 | 2.618702686585069 | 2.201559103712118 |
| Tonga | 0.2945223739795405 | 0.4306612280895774 | 0.1898490271364625 | 2.192934908100586 | 3.206588443120144 | 1.413565133443496 |
| Sudan | 45.4285402035143 | 67.34783037138338 | 31.91699511247977 | 1.458925606557055 | 2.162857838590008 | 1.025005893769639 |
| Afghanistan | 41.70193295452565 | 68.55106847692312 | 21.70945869085379 | 3.393846595757779 | 5.578921500824953 | 1.766790344084095 |
| El Salvador | 41.14598656730467 | 50.99118208665252 | 32.21422487595662 | 4.023729392395077 | 4.986506224110534 | 3.150278661433549 |
| Guinea | 10.65029272661819 | 13.84605830335964 | 7.91881828499357 | 1.165803346579681 | 1.515618539446434 | 0.8668104337197186 |
| Switzerland | 56.47806667412848 | 67.1695545530075 | 46.2414992716147 | 1.927029998255996 | 2.291823254857107 | 1.577758615125422 |
| Lesotho | 5.210525899302167 | 7.446254813585456 | 3.656609838316035 | 2.885847336269641 | 4.124104751470205 | 2.025211651494713 |
| Guyana | 3.089406388327161 | 4.02191168079792 | 2.373769574645779 | 2.743275241012584 | 3.571304434780578 | 2.107816998954539 |
| Czechia | 76.58071276860566 | 93.6890713822449 | 61.78665905707066 | 2.192114008207183 | 2.681838786399181 | 1.768635939033084 |
| France | 487.5243313359808 | 584.5813829227545 | 387.880160041879 | 2.204982520282309 | 2.64395364123645 | 1.754310334651241 |
| Myanmar | 324.3075789340592 | 429.7446277242743 | 226.6478382330437 | 3.841540454796902 | 5.090480395372139 | 2.684725538719317 |
| Brunei Darussalam | 2.279808875165757 | 2.960948489080702 | 1.752605326078757 | 3.807639873085193 | 4.945250302333046 | 2.927126915791899 |
| Armenia | 31.06751861176443 | 40.01018173320295 | 23.89898649458675 | 3.949500523631995 | 5.08634872583799 | 3.03819113634977 |
| Lebanon | 21.58399108687471 | 27.45267638749898 | 17.18697231566828 | 2.202384087533501 | 2.801212129521363 | 1.75372173702966 |
| Hungary | 73.4425261486309 | 90.13448604404812 | 59.71076200689387 | 2.309423564780443 | 2.834307546124339 | 1.877623879258244 |
| Ethiopia | 463.3978999909773 | 675.3136015700186 | 321.1800970201996 | 6.769303759770861 | 9.864962491675811 | 4.691790011056892 |
| Tuvalu | 0.04508759169647033 | 0.05910589228808077 | 0.02966877596658178 | 2.474197823173117 | 3.243457113885341 | 1.62808476014642 |
| United States Virgin Islands | 0.3138235988904052 | 0.4131253470433283 | 0.2323826744187714 | 0.974932976791232 | 1.283426503949817 | 0.7219263730543081 |
| Pakistan | 782.8546673039319 | 989.7468955634848 | 613.4044085790061 | 3.948626731885124 | 4.992166762039639 | 3.093939585891108 |
| Panama | 25.17184230040982 | 30.80881973020182 | 19.85712699318594 | 3.385292840127468 | 4.143394655055978 | 2.670531184538677 |
| Cook Islands | 0.098244414078104 | 0.1348122821719391 | 0.07509047514362113 | 2.084569699551785 | 2.860474065422541 | 1.593284775304347 |
| Zimbabwe | 59.95901202315815 | 76.71505016904298 | 44.09940459728013 | 5.415024207612987 | 6.928297177297812 | 3.982709777528672 |
| Suriname | 2.318498001365137 | 3.047698535560753 | 1.690241741385867 | 2.075597135422327 | 2.728401899124379 | 1.513161070022953 |
| Niger | 4.911795053280366 | 6.920420331970313 | 3.360750518902074 | 0.3528044307834307 | 0.4970799737200475 | 0.2413959990115013 |
| Australia | 154.5925665952547 | 187.2654351831602 | 123.5915525264572 | 2.086881550594879 | 2.527940316632625 | 1.668391543379412 |
| Israel | 62.3912755796361 | 74.53961657738667 | 51.77421399343762 | 3.121835726764576 | 3.729695152547422 | 2.590596032350122 |
| Timor-Leste | 5.557066600871633 | 8.00385980171055 | 3.595749287003189 | 3.912570477343539 | 5.635287070350657 | 2.531663468285779 |
| Greenland | 0.2445531640445024 | 0.3291849443610894 | 0.1545892874295663 | 1.801364442861885 | 2.424757235157374 | 1.138695738045337 |
| Mauritius | 5.464076409999539 | 5.961241878640905 | 4.945629265975225 | 1.61500457430806 | 1.761950635416288 | 1.461768337054144 |
| Romania | 134.896555899413 | 165.4648295703069 | 108.0687849334794 | 2.246272950505661 | 2.755290292221809 | 1.799541780526249 |
| Uruguay | 35.68212788500474 | 42.80568850654817 | 29.96104203649408 | 3.94586223789823 | 4.733612030918268 | 3.313203314580513 |
| Mongolia | 8.851498416218826 | 11.46515921651029 | 6.80634883620136 | 2.241986446305907 | 2.903997759413914 | 1.723972724395747 |
| Mozambique | 59.25361671040756 | 94.0000307432836 | 38.99862164594731 | 3.311859623185077 | 5.253939315102704 | 2.179748132849393 |
| Central African Republic | 5.276888041535305 | 7.788186235341625 | 3.547650789997701 | 1.475233239561285 | 2.177304335402727 | 0.9917990161181705 |
| Qatar | 3.217273855913778 | 4.388241200357339 | 2.245211241970029 | 2.100266392889629 | 2.8646848014082 | 1.465694848382222 |
| Sierra Leone | 2.400100769438598 | 3.274912878555378 | 1.619048222532551 | 0.3973126748762581 | 0.5421290690515025 | 0.2680172383755885 |

Table S5. The DALYs cases and rates for thyroid cancer in middle-aged and elderly patients in 2021 in various countries.

| location | Number | | | Rate | | |
| --- | --- | --- | --- | --- | --- | --- |
|  | val | upper | lower | val | upper | lower |
| Democratic People's Republic of Korea | 2580.148172951264 | 3471.322195234969 | 1838.799089552449 | 45.78165035060137 | 61.59446990780678 | 32.6272955427217 |
| Timor-Leste | 127.3589400279931 | 184.7263913219004 | 79.47809521074261 | 89.66976006750261 | 130.0605295892821 | 55.95823682737131 |
| Albania | 346.2514568106682 | 491.7931546081807 | 247.2814271745836 | 44.03587395491069 | 62.5457046959247 | 31.44897600937661 |
| Slovenia | 276.4874103179445 | 359.1425176769991 | 204.172213249269 | 37.76176467148308 | 49.0505344183573 | 27.88518674433973 |
| Japan | 34257.90581687585 | 37909.61234788678 | 28722.18040528378 | 65.62702932829973 | 72.62251390024173 | 55.02237602923541 |
| Belarus | 1686.216544494437 | 2190.177658063806 | 1307.784358067068 | 58.61833453733502 | 76.13765092968731 | 45.46280918317851 |
| Marshall Islands | 3.269095803133068 | 4.56791601312451 | 2.145725707856444 | 55.79621607037057 | 77.96419689362835 | 36.62277964095309 |
| Finland | 877.497881 | 1044.409514521389 | 714.1844723771767 | 43.59876350460177 | 51.89182151828975 | 35.48448444450801 |
| Lao People's Democratic Republic | 827.4919502314382 | 1187.793568655495 | 526.1935355143836 | 107.0287799309826 | 153.6306140833265 | 68.05848938823303 |
| Singapore | 700.2520865079005 | 852.9947007133089 | 577.7262932254862 | 46.10478562494131 | 56.16140040612973 | 38.03765445653185 |
| Kazakhstan | 1931.225058843312 | 2300.375755886366 | 1575.191239426968 | 60.85577804580974 | 72.48826633703109 | 49.63662210539336 |
| Papua New Guinea | 325.4718013570201 | 544.2648126834624 | 176.8713939596227 | 39.62871529381484 | 66.26846078937344 | 21.53546355051958 |
| Portugal | 1700.682171694505 | 2106.493098205794 | 1362.205762775458 | 43.71210294888652 | 54.14253450904695 | 35.01234947425758 |
| Maldives | 16.5621381048492 | 21.49667893547396 | 12.06417397714923 | 30.44712038099428 | 39.51856742145667 | 22.17825712200615 |
| Hungary | 1592.762332742785 | 1963.860863967386 | 1296.918972780168 | 50.08491751613619 | 61.75422871508768 | 40.78202908336642 |
| Germany | 15336.07547544588 | 18049.86340634979 | 12684.89712182603 | 48.66447692728744 | 57.275876262174 | 40.25175047543711 |
| Mongolia | 224.691053155349 | 292.8926643684082 | 172.5874528436373 | 56.91175347864831 | 74.18646570993876 | 43.71448899193118 |
| Montenegro | 120.7174307388452 | 149.7685169940801 | 96.2018358886958 | 69.22935578281219 | 85.8896506046241 | 55.17008673011077 |
| Antigua and Barbuda | 15.10557613277589 | 16.09838878822976 | 14.0444723952629 | 80.06081605897865 | 85.3228061142174 | 74.43687756091681 |
| Russian Federation | 21711.11272592236 | 23627.19822026163 | 19817.82938869531 | 51.07869566446514 | 55.58657828973467 | 46.62445858276283 |
| Jamaica | 349.0261698491196 | 450.4574141540335 | 265.7870840567148 | 65.99195566559149 | 85.17001953446773 | 50.25356544221267 |
| Sweden | 1496.913478671019 | 1803.084005947539 | 1216.190902093558 | 44.46535798651905 | 53.56006004796246 | 36.12657953320664 |
| Andorra | 10.12138895656774 | 14.4666079965811 | 7.029280497185644 | 38.24911178053925 | 54.66985892162717 | 26.5639169314952 |
| Saint Vincent and the Grenadines | 26.14344895286209 | 29.96448819442979 | 22.73766917008835 | 102.8230400302058 | 117.8513123748801 | 89.42799672243012 |
| Honduras | 465.8203334204173 | 647.7618403508975 | 315.1574250297737 | 44.29341998327624 | 61.59367718692342 | 29.96734832331345 |
| Italy | 11707.17022928482 | 13061.37853040888 | 10277.06582352382 | 51.53530979453387 | 57.49657481058219 | 45.23994787991317 |
| Egypt | 4889.711885527183 | 6207.874070685052 | 3779.591602979948 | 44.18741375462707 | 56.09939941653771 | 34.15546394026092 |
| Syrian Arab Republic | 849.0878293066322 | 1276.229366899873 | 561.5228220774028 | 35.93713331060968 | 54.01564279945973 | 23.76611678726337 |
| Dominica | 8.871243783507046 | 11.07252019730146 | 7.003865371038765 | 58.81694707607414 | 73.41155877761078 | 46.43610173606138 |
| Djibouti | 70.76218188798781 | 109.1311698141337 | 45.84730805766578 | 68.23485019529988 | 105.2334569854371 | 44.20983233847467 |
| Chile | 2474.189675240847 | 2933.601154912282 | 2110.815111210277 | 55.25602912094865 | 65.51605661732562 | 47.14079216364847 |
| Pakistan | 18050.19185773242 | 23024.53609562098 | 13939.62559882748 | 91.04304165491637 | 116.1330480784199 | 70.30982961570687 |
| Cameroon | 248.0616553587655 | 362.1233322627879 | 167.4717370262962 | 12.18689956629774 | 17.79057982386282 | 8.227636941231841 |
| Uganda | 2903.57648191993 | 3804.930383265355 | 2085.345511913512 | 122.436682753302 | 160.444561090472 | 87.93389410026835 |
| Ecuador | 3607.0203060592 | 4567.937080295782 | 2796.856946334308 | 130.3340499244787 | 165.0552780296373 | 101.0600611986625 |
| Guam | 12.08274974139125 | 14.46433762912875 | 8.9527772670226 | 32.06212602367758 | 38.38177781051592 | 23.75660169587713 |
| Malawi | 822.6104051434453 | 1137.679103893529 | 565.148732432885 | 70.36102636519699 | 97.31006187580095 | 48.33934097397401 |
| Paraguay | 815.0628318100174 | 1055.244440100467 | 574.4465564204315 | 82.32083408404323 | 106.5790256669045 | 58.01874139717722 |
| Lebanon | 433.7055973424079 | 569.4647023125405 | 338.9929102233589 | 44.25438754197698 | 58.10695499905999 | 34.59010839365122 |
| Afghanistan | 1003.717388334597 | 1631.786589854801 | 504.4104029617659 | 81.68596993374068 | 132.8003996606326 | 41.05065179648506 |
| Nigeria | 397.8663552342206 | 746.7412660802397 | 265.7958302497427 | 2.652174954758006 | 4.97777320833374 | 1.771793555281869 |
| Chad | 92.08513916506328 | 123.6548900122967 | 65.22769976405212 | 9.91738563933198 | 13.31738477630919 | 7.02489303694396 |
| Congo | 187.5493928329255 | 270.6312653310168 | 125.1974772261165 | 43.16159191995885 | 62.2815998418354 | 28.81226294481144 |
| Tuvalu | 1.015581330964351 | 1.342773207618995 | 0.6590018192405539 | 55.73039108504806 | 73.685163085927 | 36.16296203196293 |
| Morocco | 2034.075897416246 | 2631.307263675595 | 1467.822597537326 | 33.85621037399574 | 43.79683786174558 | 24.43119783143429 |
| Equatorial Guinea | 32.00865889618359 | 50.88661478800557 | 18.37669892204626 | 40.46763176923672 | 64.33449136069656 | 23.23313474405405 |
| Mozambique | 1419.378522907817 | 2291.732310391259 | 917.9223193064512 | 79.33325729311642 | 128.0916866733695 | 51.30538919487545 |
| Senegal | 129.6405965590131 | 193.7819814301782 | 88.82254614288169 | 10.04058741222965 | 15.00829967701579 | 6.879253585650082 |
| Niue | 0.2254129657734875 | 0.2891447281811398 | 0.1536041425738069 | 57.61158060579284 | 73.90029565152493 | 39.25851119924979 |
| Eswatini | 65.90357798756656 | 97.83661937064149 | 42.15814113201978 | 72.78994764872395 | 108.0597233045629 | 46.56331233709613 |
| Guinea-Bissau | 15.76337654496961 | 20.63425969007655 | 11.48699154861478 | 13.72275702318276 | 17.96308876289174 | 9.999963745032666 |
| Palau | 2.30633737213577 | 3.00143908147062 | 1.611103899032112 | 55.07872986098024 | 71.67878141324117 | 38.4755315960504 |
| Ireland | 487.4508313432272 | 580.4507037511061 | 404.7609203060117 | 37.14283745228456 | 44.22925298759177 | 30.84202160151305 |
| Belize | 19.57522157819919 | 22.30154121922188 | 17.02385763953674 | 39.08291568116906 | 44.52615014083267 | 33.98898909196652 |
| Panama | 528.9542649909841 | 649.8048814907332 | 414.7511410318047 | 71.13762531396888 | 87.39049714905619 | 55.77875673195389 |
| United Arab Emirates | 444.3491381649702 | 582.8441788659676 | 342.9802397186432 | 61.88027336005479 | 81.16715892256914 | 47.76359211257705 |
| Botswana | 103.8162392598943 | 155.5190324419942 | 69.09130433636903 | 42.82434089356518 | 64.15181389937167 | 28.50025767428034 |
| Cote d'Ivoire | 466.005138945554 | 631.6425279839424 | 339.6615811916433 | 25.98760093357477 | 35.22466294484121 | 18.94182893443745 |
| Tajikistan | 4.681934020089513 | 6.735701287321398 | 3.11646710712304 | 0.4558201826807832 | 0.6557692992032649 | 0.3034106418399114 |
| Kenya | 967.1180862552196 | 1375.732443994048 | 714.9147199080833 | 25.85343283489245 | 36.77669443377199 | 19.11141974956507 |
| Poland | 6370.120658982577 | 6992.978848796538 | 5720.54789929129 | 52.57891418337283 | 57.71997964568213 | 47.21734692019942 |
| Canada | 4930.878083084713 | 5859.687731042955 | 4073.709370844753 | 40.18257373349745 | 47.75160333321479 | 33.19735844298229 |
| Ghana | 122.6138966725914 | 164.3327601097834 | 76.05090071408127 | 4.496010462849823 | 6.025759142256617 | 2.788636970185247 |
| Namibia | 101.3733073378004 | 137.1517493713752 | 69.51434002240033 | 44.86598607752334 | 60.70087520474508 | 30.76578532887897 |
| Uzbekistan | 1015.415859743886 | 1352.098859134256 | 764.5816913000367 | 21.8432380577507 | 29.0858341183798 | 16.44738925180543 |
| Jordan | 583.4033369781805 | 798.2314946635531 | 432.9764342438583 | 46.40343617938604 | 63.49069652370104 | 34.43860029611806 |
| Netherlands | 2431.544654108338 | 2815.723932973766 | 2029.120680339538 | 41.55866451296514 | 48.12485186890166 | 34.68064856142807 |
| Colombia | 8672.908724 | 10803.36605006656 | 7062.074396064682 | 90.6875714101903 | 112.9645268206859 | 73.8440120261419 |
| Serbia | 1489.642500232104 | 1970.528235325433 | 1099.235625724277 | 53.10252359738288 | 70.2450568504684 | 39.18536544507307 |
| Bangladesh | 10603.55872081967 | 16638.18442922824 | 6868.502635786914 | 45.38000492151423 | 71.20636676448513 | 29.39510136379476 |
| Guyana | 75.0041749803464 | 98.92420087619335 | 57.21093505530755 | 66.60085153367355 | 87.84092375349825 | 50.80113197324817 |
| Rwanda | 791.0804304433445 | 1097.380160884746 | 560.3051423547146 | 74.80905844387625 | 103.7745005836759 | 52.98563651401087 |
| El Salvador | 859.1794146340504 | 1058.362542708042 | 663.5736643901628 | 84.0204781175646 | 103.4989029595482 | 64.89189056284938 |
| Bahamas | 51.31252343143937 | 63.00011384457056 | 41.75911062561138 | 71.30840437541197 | 87.55050995938923 | 58.03214006474225 |
| Angola | 678.3364806285416 | 1067.736473239359 | 405.9475883918331 | 35.08098214522769 | 55.21926834719365 | 20.9940649028289 |
| Sierra Leone | 56.29655143180734 | 78.70927513191543 | 37.23822809090838 | 9.31933097163752 | 13.02953319228652 | 6.164416177372302 |
| India | 118081.5784763394 | 135808.0987288199 | 98959.73246008877 | 58.73379685448287 | 67.55097098850487 | 49.22258745253362 |
| Algeria | 2211.195498583604 | 2918.427284079499 | 1664.927856570757 | 36.40804723175194 | 48.05284673800299 | 27.41357427613984 |
| Somalia | 743.224158413102 | 1029.765079814951 | 488.8969337830576 | 78.80978347404701 | 109.1939249426858 | 51.8415084553165 |
| Benin | 76.47413819674264 | 107.2640371281234 | 54.1210894635928 | 9.24648336493106 | 12.9692881586835 | 6.54377761184593 |
| Mauritania | 38.37774623393409 | 55.21249746371497 | 26.59729846886177 | 10.69739888714734 | 15.38991126067552 | 7.413721204669119 |
| Switzerland | 1011.931955330459 | 1191.97652287929 | 840.3348822548913 | 34.52708899132529 | 40.67020441862014 | 28.67220183066016 |
| Suriname | 52.51314466071792 | 68.48497047176056 | 37.84098708227862 | 47.0115275344759 | 61.31004143500327 | 33.87652020544536 |
| Estonia | 277.7591324856114 | 353.1693444350782 | 212.5076711998933 | 63.49138113194073 | 80.72897280094568 | 48.57592052823865 |
| Croatia | 735.7855017156869 | 868.4261327384077 | 620.8790234438895 | 49.3376600823313 | 58.23179886495931 | 41.63267438607553 |
| Samoa | 17.60976825992267 | 30.50591846469917 | 12.28382435549985 | 71.52182398686183 | 123.8993551070929 | 49.89057836945351 |
| Lithuania | 576.8355321393034 | 701.79291539825 | 464.6127559125012 | 59.60398122866599 | 72.51573355870596 | 48.00808625519937 |
| Taiwan (Province of China) | 3995.785459766721 | 4484.755424500425 | 3476.49682414285 | 53.06533730373722 | 59.55901830119136 | 46.16901444435457 |
| Myanmar | 7566.122719796995 | 10128.12352596994 | 5146.392211072226 | 89.62345748931098 | 119.9712827682781 | 60.96087529026081 |
| Bosnia and Herzegovina | 468.5025455586565 | 601.3088886113444 | 349.5414108866012 | 42.77512904340878 | 54.90058815929891 | 31.91376247243376 |
| American Samoa | 8.523111460164019 | 11.243162588015 | 5.490152327467028 | 100.9104814650714 | 133.1148789088861 | 65.0013691913656 |
| France | 9747.662029448062 | 11551.57889916259 | 7978.138343090534 | 44.08687527380088 | 52.24565814903279 | 36.0836464155576 |
| Malaysia | 4208.608051736829 | 5538.455835005519 | 3428.04058970251 | 85.84108535998041 | 112.965392417331 | 69.92020194341713 |
| North Macedonia | 309.9461347715829 | 391.1021120393711 | 243.2141947175526 | 52.27578225962952 | 65.96361934088532 | 41.02071572815636 |
| China | 136671.948146868 | 167935.1566997059 | 109610.4875586707 | 36.06547032440256 | 44.31531482867752 | 28.92439772675568 |
| Republic of Moldova | 513.0614177778738 | 612.3225414347921 | 427.2530422836066 | 48.59304374461371 | 57.99425762830587 | 40.4659657777953 |
| Israel | 1198.605770831843 | 1426.581020242895 | 1005.527787655258 | 59.97393518446272 | 71.38099926221305 | 50.31300518531636 |
| Ukraine | 6499.071153780646 | 8848.568084854312 | 4677.391532281249 | 47.86446956407661 | 65.16808444800475 | 34.44813256212917 |
| Thailand | 12582.41044314655 | 18230.62970052713 | 9124.50348729088 | 64.73103831246851 | 93.78867387433108 | 46.94160848486637 |
| Comoros | 58.0543904025298 | 88.41807422444414 | 39.05743911371141 | 71.82234323027227 | 109.3869598953016 | 48.32014905798061 |
| Liberia | 35.28078730596803 | 49.11787006689855 | 24.18079029414413 | 10.68720849851792 | 14.8787189428525 | 7.324812377096296 |
| Zimbabwe | 1451.402631544441 | 1880.152732516517 | 1026.608442080707 | 131.0792176123714 | 169.8005390191281 | 92.7151628759288 |
| Cyprus | 160.3397908780678 | 209.1828405275493 | 122.5177798861696 | 45.87668987919937 | 59.85174516181245 | 35.05499266118013 |
| Nepal | 2140.96705171264 | 2978.327653113524 | 1561.664043428876 | 54.0941151822297 | 75.25104087382616 | 39.45732587225574 |
| Luxembourg | 100.8040009307025 | 115.747202663353 | 88.79866256571289 | 56.7264310406168 | 65.13556653907746 | 49.97054841102738 |
| United States Virgin Islands | 6.518755130554546 | 8.601877883326585 | 4.70796614136769 | 20.25134300567401 | 26.72282913215498 | 14.62589639869259 |
| Eritrea | 377.5137730916831 | 518.9918190110031 | 271.8241444926226 | 85.67998483830739 | 117.7896393551422 | 61.69281821979404 |
| Zambia | 1154.601951677503 | 1839.317713347223 | 626.2610777029981 | 106.8919095006731 | 170.2822191426645 | 57.97863267454523 |
| Puerto Rico | 491.9372218968592 | 584.6064807888505 | 404.1026327697379 | 42.14085054474196 | 50.07918335477307 | 34.61667036827038 |
| Cabo Verde | 6.522488472585635 | 11.09974463214811 | 2.307575216867668 | 8.509866866744327 | 14.48179624562768 | 3.010684949935681 |
| Northern Mariana Islands | 3.858121565928159 | 4.656582587245789 | 2.884357400027799 | 39.69119856340172 | 47.90552628758023 | 29.67340461828577 |
| Sao Tome and Principe | 0.7261756424000907 | 1.060258450413551 | 0.5232567926687204 | 3.971674344575687 | 5.7988743222084 | 2.861849747808421 |
| Democratic Republic of the Congo | 2014.890298325828 | 3336.543035121562 | 1173.454924279162 | 33.36013481288281 | 55.24247426925727 | 19.42865797871029 |
| Mauritius | 124.3228447276467 | 135.9404882270359 | 112.6026791185092 | 36.74581902967147 | 40.17961936230547 | 33.28171647141196 |
| South Africa | 3383.667897474697 | 3801.155999552482 | 2711.704842121864 | 42.89339383438581 | 48.18572219703555 | 34.3751890788549 |
| Guinea | 243.9922744879196 | 321.8229521229595 | 177.2353125561718 | 26.70790535425274 | 35.2274143276277 | 19.40054849325159 |
| Tokelau | 0.1376672023983941 | 0.1849285078842317 | 0.08932403317617013 | 54.46968352861036 | 73.16918717300628 | 35.34212749181166 |
| Cambodia | 2968.546576945827 | 4173.531384271519 | 1848.897185242956 | 136.5595553073058 | 191.991459498533 | 85.05326457955567 |
| New Zealand | 536.6835430156292 | 633.8948073850959 | 436.4908505527488 | 37.62444042550584 | 44.43947970247991 | 30.60038679520684 |
| Palestine | 266.3968674497658 | 343.8894439416836 | 158.8258835912895 | 61.91525633126923 | 79.92587628787335 | 36.91389237694152 |
| Gabon | 78.43449250592802 | 110.9217618853137 | 53.52282600535061 | 43.87895121414137 | 62.05344642198627 | 29.94250866038636 |
| Sri Lanka | 2911.056555518362 | 4169.111063123932 | 1838.273566021158 | 60.44170716514553 | 86.5624508526371 | 38.16772036127409 |
| Cook Islands | 2.104434787475599 | 2.903982622830333 | 1.596716201 | 44.65231976615807 | 61.61728623842654 | 33.87944486829453 |
| Greece | 1669.025458995721 | 1862.819979659743 | 1469.941602427262 | 45.41010314424238 | 50.6827783600162 | 39.99351802722023 |
| San Marino | 6.990479602025695 | 9.87117899627498 | 4.51913398376186 | 59.10612141676923 | 83.46310088815962 | 38.21032277463118 |
| Tonga | 6.106188145408411 | 8.774023394652483 | 3.92039660833958 | 45.46504551951727 | 65.32903401071066 | 29.19022571991162 |
| Qatar | 91.07589640421152 | 127.3892514207427 | 61.82743832091604 | 59.45519498393275 | 83.16089197149259 | 40.36152863555185 |
| Burundi | 512.5943781092565 | 748.5681135610109 | 333.245056460866 | 64.03201448596414 | 93.50926646537089 | 41.62814340919278 |
| Fiji | 180.5335597501116 | 251.0097577174116 | 113.28957938003 | 132.0262828819658 | 183.5663425925009 | 82.84998133039656 |
| Monaco | 8.896053337635314 | 11.17148731070193 | 6.843900800046832 | 56.86066891225189 | 71.40450007689527 | 43.74397979533688 |
| Brunei Darussalam | 52.19110753979497 | 68.19242143783873 | 39.95717976552068 | 87.16736927105433 | 113.8920835590069 | 66.73478314280509 |
| Denmark | 626.9146144665085 | 742.8539059798179 | 523.6633971016832 | 32.56322615407272 | 38.58534987327101 | 27.20014693379435 |
| United States of America | 50212.37595440632 | 54156.97291573192 | 45930.29299422215 | 50.0882776238099 | 54.02312563602741 | 45.81677769891801 |
| Norway | 756.3379794256928 | 843.1399696900953 | 656.0712217240557 | 46.63757607172148 | 51.98999064596617 | 40.45489231528001 |
| Costa Rica | 714.6440419763703 | 830.6966768489374 | 607.9076635322012 | 74.63933857570248 | 86.7601867155689 | 63.49150521938371 |
| Haiti | 876.177547166571 | 1202.338722807112 | 613.1458887268684 | 73.9335243555679 | 101.4556233879415 | 51.73841380014968 |
| Azerbaijan | 685.9562826515122 | 885.4741792241821 | 505.8824511807375 | 35.80522961350918 | 46.21957274216322 | 26.40581882559018 |
| Bhutan | 55.80534005434854 | 81.2831095314367 | 38.97884398939651 | 56.03501974062679 | 81.61764882604032 | 39.13927036884789 |
| Bulgaria | 1252.78598 | 1592.14513879259 | 998.2673375405452 | 52.70654631143051 | 66.9838845829731 | 41.99857317749136 |
| Czechia | 1619.990463342003 | 2012.361555738671 | 1296.250445536258 | 46.37203885244939 | 57.60361579868668 | 37.10501844492781 |
| Iceland | 79.52350933127457 | 95.62478682290345 | 66.67168577338308 | 81.65789757147414 | 98.19132874469126 | 68.46113474596541 |
| Latvia | 502.856075444956 | 633.0692794747334 | 386.8766283628855 | 76.41963272325313 | 96.20828739719444 | 58.79409893286348 |
| Nicaragua | 462.7355926353995 | 598.6675385356502 | 336.6148608697284 | 56.84478269177703 | 73.5433510503568 | 41.35147354450264 |
| Australia | 3250.526293711413 | 3913.166763054781 | 2642.96737979381 | 43.87962177916338 | 52.82476190204333 | 35.6780405758856 |
| Trinidad and Tobago | 213.4835763899597 | 273.0663963405113 | 162.7937171943869 | 61.2861910805161 | 78.39103891169204 | 46.73430634522531 |
| Barbados | 62.67229231510206 | 76.70992933323511 | 49.03839215791986 | 68.7808165223761 | 84.18666973893795 | 53.81805147014619 |
| Ethiopia | 10331.12216267791 | 15451.49101005867 | 6992.409910458518 | 150.9167480038844 | 225.7149550968885 | 102.1449313811036 |
| Venezuela (Bolivarian Republic of) | 3642.14490255038 | 4708.259699088077 | 2762.675964008679 | 69.61034972751318 | 89.98642641372606 | 52.80153458578074 |
| Bolivia (Plurinational State of) | 2531.003619845017 | 3491.32001489662 | 1584.180568999759 | 163.7690048338879 | 225.9064348676304 | 102.5046638527367 |
| United Kingdom | 8251.954426800654 | 8701.196891323625 | 7611.959583430991 | 39.23861787120457 | 41.37479707012063 | 36.19539782905752 |
| Madagascar | 1053.094229581559 | 1453.09388913865 | 716.6831224775677 | 57.0263374763365 | 78.68680710533256 | 38.80926555094673 |
| Malta | 76.95990935006984 | 92.43111812141632 | 63.65550631965152 | 48.76954989338726 | 58.5736660164074 | 40.33854012644122 |
| Central African Republic | 135.0504670747899 | 199.8441829321472 | 90.49578722111796 | 37.75538470379237 | 55.86944029775032 | 25.29945534148874 |
| Romania | 2859.286454529471 | 3525.350504834225 | 2283.143564013441 | 47.61231877073245 | 58.70349637366128 | 38.01849199017833 |
| Grenada | 17.06802071392473 | 19.84551265302266 | 14.594974250498 | 84.96988865848822 | 98.79710300106636 | 72.65829810169912 |
| Belgium | 1917.462952736964 | 2313.584908519696 | 1566.559748600956 | 50.71616488386225 | 61.1934397615433 | 41.43490876684846 |
| Slovakia | 903.1338317822316 | 1164.252601985974 | 715.0540201122013 | 55.05984721888629 | 70.97903780554589 | 43.59350044824974 |
| Armenia | 693.011434588678 | 883.1912073462177 | 531.3997105211396 | 88.10002041020715 | 112.2768824723639 | 67.55491036115073 |
| Oman | 99.55414551087814 | 127.7594296432683 | 69.29789518521108 | 31.8721598624069 | 40.90205329596479 | 22.18565165857433 |
| Indonesia | 34898.60571524833 | 46213.54574467456 | 22447.13932483376 | 83.33089894140231 | 110.3487154070086 | 53.59928456350903 |
| Kiribati | 0.3022441829354107 | 0.5997710038992319 | 0.1982316854378105 | 2.403179818119624 | 4.768851324334658 | 1.576163951707177 |
| Seychelles | 5.91684419529208 | 7.209845577309565 | 4.596251421180258 | 28.52460201470078 | 34.75805157145158 | 22.15813670621723 |
| Mali | 618.4335450022243 | 869.756429013026 | 450.1486676215327 | 42.7280037693428 | 60.09207663071545 | 31.10108454228221 |
| Iraq | 2253.531899389362 | 3047.564719572761 | 1577.763575257046 | 58.00310348065581 | 78.44051900985892 | 40.60966873752343 |
| Burkina Faso | 160.9567672063962 | 219.6617167422271 | 107.0865795518929 | 10.70608135376595 | 14.61085638440134 | 7.122891770738391 |
| Bermuda | 16.21581586966574 | 20.51363039777869 | 13.2808123096346 | 69.62308355336357 | 88.07587694918782 | 57.02155923094421 |
| Nauru | 0.7378456840676373 | 1.114465786972381 | 0.4432410371011741 | 79.8294247655251 | 120.5769236250054 | 47.95538930199651 |
| South Sudan | 459.2143974375146 | 652.796790917969 | 317.13432597019 | 73.74302549114853 | 104.8294884956303 | 50.9270720052285 |
| Sudan | 1098.981535808783 | 1634.349223296232 | 751.9728824793949 | 35.2935026426582 | 52.48669495523554 | 24.14941111404766 |
| Georgia | 1060.034462226986 | 1314.899405716145 | 847.0234481737543 | 100.9683047904478 | 125.24419601058 | 80.67900122812298 |
| Turkey | 9293.022036229611 | 11683.97540427604 | 7218.887004835235 | 56.24888685029472 | 70.72087076890168 | 43.69454379178313 |
| Viet Nam | 19755.28402541792 | 26636.14927436983 | 14317.17537346841 | 113.0903054233896 | 152.4802302445134 | 81.9595270664058 |
| Vanuatu | 14.01576879161696 | 18.94991257986081 | 9.404480960258816 | 48.87850321990685 | 66.08580498314737 | 32.7971272737107 |
| Republic of Korea | 9071.296661153294 | 13190.07866410892 | 7230.503763206818 | 54.24197362751217 | 78.87030110119305 | 43.23492099172837 |
| Spain | 6302.813807239749 | 7483.266501413382 | 5319.016976981373 | 40.72880082065073 | 48.35688950129257 | 34.37150289419834 |
| Cuba | 2365.215150232156 | 2778.17037819223 | 1970.664829833843 | 69.2180996354985 | 81.30324804626142 | 57.67157145354111 |
| Saint Lucia | 31.73167054703056 | 38.15634368072109 | 25.73579465914875 | 75.40460470494122 | 90.67168423945132 | 61.15648465984237 |
| Guatemala | 1385.986345241982 | 1600.268055168228 | 1190.209739839287 | 75.70498306268453 | 87.40942248685307 | 65.01132461001542 |
| Bahrain | 107.1948815038164 | 143.2159671467991 | 74.26947527404454 | 66.01794083505186 | 88.20218944311728 | 45.74022337361006 |
| Saudi Arabia | 2038.806536021317 | 2620.599004405929 | 1550.025103898409 | 67.7186355106645 | 87.04278001055013 | 51.4838378182336 |
| Greenland | 5.865642484942262 | 8.064083439171604 | 3.653638602390166 | 43.20598283076098 | 59.39957157516764 | 26.91249034183658 |
| Philippines | 18861.09293555803 | 22314.54376178919 | 14664.99549931135 | 135.2527460813479 | 160.0174142424427 | 105.162565039542 |
| Solomon Islands | 26.6047222938408 | 40.85396024963748 | 16.41770469842453 | 49.04482887482021 | 75.31277594902939 | 30.26543590864599 |
| United Republic of Tanzania | 2958.53980128376 | 4148.239040706141 | 2106.659003467674 | 71.77121993596543 | 100.632134949914 | 51.10544282769266 |
| Niger | 119.8718448766404 | 171.4762304440065 | 79.41038538475868 | 8.610155256868538 | 12.31679522831118 | 5.703889415184734 |
| Kuwait | 209.9590784035223 | 256.7736953650015 | 169.9823708909641 | 45.03397156558498 | 55.07520505321317 | 36.45939635266552 |
| Gambia | 26.39419041431423 | 35.88631474642501 | 18.94030507553259 | 17.0681215969389 | 23.2063182898697 | 12.24797673419031 |
| Saint Kitts and Nevis | 7.260351944480168 | 8.75969527512786 | 5.831552896177565 | 56.37197272396072 | 68.01341131887379 | 45.27826520194849 |
| Turkmenistan | 346.6899748628383 | 450.4399671879901 | 265.9205346937434 | 48.98819624027088 | 63.6483403242236 | 37.57526401808638 |
| Yemen | 834.6588513341837 | 1256.820358180677 | 552.0543204065914 | 37.11366899918234 | 55.88536524879608 | 24.54746784795723 |
| Lesotho | 123.2447658550883 | 177.901325002743 | 85.33882754124292 | 68.25905601960767 | 98.53056578161618 | 47.26486978468982 |
| Togo | 65.86397193076931 | 96.30193544606524 | 43.55281304087414 | 10.54350619048359 | 15.41601611269967 | 6.971935345656074 |
| Micronesia (Federated States of) | 7.665293248083724 | 10.4969880838836 | 5.011805653458486 | 58.25060856994904 | 79.76941315201262 | 38.08604836106583 |
| Kyrgyzstan | 486.9255032721506 | 619.6683168244366 | 378.1652670377958 | 57.57319331332841 | 73.26846417970043 | 44.71357913529676 |
| Austria | 1816.418059998122 | 2155.302933397057 | 1506.804909631065 | 61.46570566959483 | 72.93321876194891 | 50.98871736442616 |
| Argentina | 5465.714430223056 | 6408.289144470626 | 4598.807045814614 | 58.56137436349372 | 68.66041473804955 | 49.27305743348753 |
| Dominican Republic | 794.6121406662581 | 1049.40832587731 | 592.8005947585339 | 47.55241353173118 | 62.80032247420531 | 35.47529364474388 |
| Mexico | 19255.46923567678 | 21689.21027101656 | 17105.6550643604 | 89.35068356354523 | 100.6439126436942 | 79.37495337537537 |
| Iran (Islamic Republic of) | 4311.345444278258 | 5331.957745891761 | 1872.119793640873 | 33.18732266052586 | 41.04366129140528 | 14.41096392152306 |
| Tunisia | 852.721752321269 | 1223.497625722767 | 594.1344078373861 | 36.48578253519121 | 52.35033372014312 | 25.42149152642038 |
| Peru | 5334.33650567928 | 7288.581150050797 | 3544.014444548841 | 95.36767640492054 | 130.3058117591576 | 63.36016154253814 |
| Uruguay | 687.2581987654285 | 827.461110876306 | 577.6300545868094 | 75.99956434588351 | 91.50372313160892 | 63.87647696389286 |
| Brazil | 20633.01704546381 | 21984.00925592129 | 18865.21829216523 | 47.64278376611041 | 50.7622999091302 | 43.56083813693645 |
| Libya | 508.3400080150624 | 704.7722725099281 | 375.1049889883668 | 60.78320993033841 | 84.27100034940258 | 44.85203787249733 |

Table S6. Estimated annual percentage change (EAPC) in incidence rates of thyroid cancer for both sex by Country, 1990-2021.

| location | val | upper | lower |
| --- | --- | --- | --- |
| Iran (Islamic Republic of) | 5.451 | 4.923 | 5.982 |
| Cabo Verde | 4.533 | 4.218 | 4.848 |
| Saudi Arabia | 4.015 | 3.779 | 4.251 |
| Ecuador | 3.914 | 3.316 | 4.515 |
| Syrian Arab Republic | 3.815 | 3.595 | 4.035 |
| Viet Nam | 3.684 | 3.467 | 3.902 |
| Uzbekistan | 3.572 | 2.823 | 4.326 |
| Afghanistan | 3.261 | 3.044 | 3.478 |
| Armenia | 3.102 | 2.746 | 3.459 |
| Yemen | 3.004 | 2.76 | 3.248 |
| Georgia | 2.989 | 1.69 | 4.304 |
| Australia | 2.923 | 2.472 | 3.376 |
| Iraq | 2.873 | 2.521 | 3.226 |
| Oman | 2.83 | 2.53 | 3.13 |
| Algeria | 2.716 | 2.557 | 2.876 |
| Egypt | 2.69 | 2.516 | 2.864 |
| Sudan | 2.606 | 2.413 | 2.799 |
| India | 2.577 | 2.427 | 2.726 |
| Lesotho | 2.476 | 2.078 | 2.876 |
| Honduras | 2.47 | 2.234 | 2.706 |
| Libya | 2.447 | 2.092 | 2.803 |
| Cuba | 2.425 | 2.076 | 2.776 |
| Zambia | 2.397 | 2.047 | 2.747 |
| Republic of Korea | 2.335 | 1.336 | 3.344 |
| Nicaragua | 2.323 | 2.111 | 2.535 |
| Morocco | 2.3 | 2.164 | 2.436 |
| Belize | 2.276 | 2.044 | 2.508 |
| Peru | 2.252 | 2.043 | 2.462 |
| Latvia | 2.223 | 1.821 | 2.627 |
| Guam | 2.201 | 1.219 | 3.193 |
| Uganda | 2.192 | 2.038 | 2.347 |
| Kenya | 2.183 | 1.957 | 2.41 |
| Equatorial Guinea | 2.077 | 1.871 | 2.284 |
| China | 2.073 | 1.909 | 2.237 |
| Tunisia | 2.019 | 1.931 | 2.106 |
| Nepal | 2.007 | 1.797 | 2.217 |
| Belarus | 2.003 | 1.561 | 2.446 |
| Cambodia | 1.964 | 1.827 | 2.101 |
| Antigua and Barbuda | 1.922 | 1.442 | 2.405 |
| Lebanon | 1.922 | 1.686 | 2.158 |
| Panama | 1.913 | 1.739 | 2.087 |
| Namibia | 1.883 | 1.782 | 1.984 |
| Kuwait | 1.835 | 0.846 | 2.833 |
| Mozambique | 1.784 | 1.634 | 1.935 |
| Turkey | 1.776 | 1.559 | 1.993 |
| Zimbabwe | 1.765 | 1.266 | 2.267 |
| South Africa | 1.724 | 1.524 | 1.923 |
| Jamaica | 1.723 | 1.394 | 2.053 |
| Estonia | 1.677 | 1.209 | 2.147 |
| Timor-Leste | 1.674 | 1.512 | 1.836 |
| Nigeria | 1.667 | 1.462 | 1.872 |
| El Salvador | 1.665 | 1.568 | 1.762 |
| Mexico | 1.659 | 1.533 | 1.785 |
| Jordan | 1.619 | 1.184 | 2.056 |
| Bahamas | 1.612 | 1.327 | 1.898 |
| Taiwan (Province of China) | 1.585 | 1.245 | 1.926 |
| Barbados | 1.566 | 1.351 | 1.781 |
| Bhutan | 1.563 | 1.485 | 1.641 |
| Malawi | 1.529 | 1.355 | 1.704 |
| Paraguay | 1.527 | 1.454 | 1.6 |
| Venezuela (Bolivarian Republic of) | 1.524 | 1.406 | 1.642 |
| Philippines | 1.522 | 1.488 | 1.555 |
| Sao Tome and Principe | 1.487 | 1.359 | 1.615 |
| Dominican Republic | 1.482 | 1.357 | 1.606 |
| Bangladesh | 1.471 | 1.399 | 1.542 |
| Northern Mariana Islands | 1.461 | 0.862 | 2.063 |
| Qatar | 1.41 | 1.1 | 1.722 |
| Nauru | 1.391 | 1.328 | 1.454 |
| Guyana | 1.389 | 1.196 | 1.583 |
| Eswatini | 1.329 | 1.02 | 1.639 |
| Djibouti | 1.322 | 1.245 | 1.4 |
| Indonesia | 1.295 | 1.177 | 1.414 |
| Grenada | 1.275 | 0.809 | 1.743 |
| Solomon Islands | 1.257 | 1.154 | 1.36 |
| Democratic People's Republic of Korea | 1.256 | 1.094 | 1.417 |
| American Samoa | 1.254 | 0.991 | 1.519 |
| Bolivia (Plurinational State of) | 1.248 | 1.178 | 1.318 |
| South Sudan | 1.224 | 1.023 | 1.426 |
| Eritrea | 1.22 | 1.149 | 1.292 |
| Montenegro | 1.214 | 1.113 | 1.316 |
| Greenland | 1.214 | 0.693 | 1.738 |
| Pakistan | 1.17 | 1.005 | 1.335 |
| Netherlands | 1.161 | 0.944 | 1.379 |
| Malaysia | 1.157 | 0.999 | 1.315 |
| Saint Kitts and Nevis | 1.154 | 0.895 | 1.413 |
| France | 1.137 | 0.604 | 1.673 |
| United States of America | 1.131 | 0.923 | 1.339 |
| Comoros | 1.098 | 1.003 | 1.192 |
| Tonga | 1.096 | 0.922 | 1.269 |
| Gambia | 1.072 | 0.895 | 1.249 |
| Angola | 1.065 | 0.947 | 1.182 |
| Saint Vincent and the Grenadines | 1.063 | 0.544 | 1.585 |
| Lithuania | 1.055 | 0.57 | 1.541 |
| Russian Federation | 1.048 | 0.738 | 1.359 |
| Tokelau | 1.043 | 0.875 | 1.213 |
| Colombia | 1.024 | 0.661 | 1.389 |
| Norway | 0.981 | 0.591 | 1.373 |
| Serbia | 0.976 | 0.788 | 1.163 |
| Bermuda | 0.969 | 0.608 | 1.332 |
| Uruguay | 0.948 | 0.849 | 1.047 |
| Monaco | 0.945 | 0.822 | 1.067 |
| Vanuatu | 0.917 | 0.833 | 1 |
| Puerto Rico | 0.915 | 0.559 | 1.273 |
| Ghana | 0.908 | 0.77 | 1.045 |
| Palestine | 0.907 | 0.781 | 1.033 |
| Lao People's Democratic Republic | 0.906 | 0.849 | 0.963 |
| Albania | 0.897 | 0.724 | 1.071 |
| Tuvalu | 0.886 | 0.729 | 1.044 |
| Botswana | 0.882 | 0.518 | 1.248 |
| Republic of Moldova | 0.882 | 0.687 | 1.077 |
| Myanmar | 0.879 | 0.78 | 0.977 |
| Kyrgyzstan | 0.868 | -0.068 | 1.814 |
| Niue | 0.864 | 0.786 | 0.942 |
| United Republic of Tanzania | 0.861 | 0.759 | 0.962 |
| Marshall Islands | 0.849 | 0.744 | 0.954 |
| Thailand | 0.837 | 0.573 | 1.101 |
| Somalia | 0.831 | 0.792 | 0.87 |
| Azerbaijan | 0.826 | 0.695 | 0.958 |
| Singapore | 0.817 | 0.454 | 1.182 |
| Suriname | 0.808 | 0.594 | 1.022 |
| Guatemala | 0.748 | 0.369 | 1.129 |
| Micronesia (Federated States of) | 0.745 | 0.67 | 0.82 |
| Costa Rica | 0.745 | 0.502 | 0.989 |
| Japan | 0.699 | 0.469 | 0.93 |
| Cote d'Ivoire | 0.698 | 0.621 | 0.774 |
| Trinidad and Tobago | 0.682 | 0.402 | 0.964 |
| Ukraine | 0.652 | 0.263 | 1.042 |
| Mongolia | 0.642 | 0.205 | 1.08 |
| Chile | 0.605 | 0.405 | 0.807 |
| Samoa | 0.59 | 0.478 | 0.702 |
| Madagascar | 0.565 | 0.431 | 0.699 |
| Bulgaria | 0.557 | 0.206 | 0.91 |
| Canada | 0.541 | 0.293 | 0.791 |
| Denmark | 0.536 | 0.1 | 0.974 |
| United Kingdom | 0.534 | 0.37 | 0.698 |
| Bahrain | 0.531 | 0.357 | 0.706 |
| Sri Lanka | 0.521 | 0.281 | 0.761 |
| Ireland | 0.51 | 0.066 | 0.956 |
| New Zealand | 0.484 | 0.036 | 0.934 |
| Brunei Darussalam | 0.471 | 0.34 | 0.602 |
| Spain | 0.456 | 0.207 | 0.705 |
| Papua New Guinea | 0.437 | 0.35 | 0.524 |
| North Macedonia | 0.431 | 0.296 | 0.567 |
| Brazil | 0.421 | 0.283 | 0.559 |
| Romania | 0.398 | 0.094 | 0.703 |
| Democratic Republic of the Congo | 0.368 | 0.193 | 0.542 |
| Seychelles | 0.348 | -0.02 | 0.716 |
| Congo | 0.329 | 0.199 | 0.459 |
| Haiti | 0.317 | 0.223 | 0.411 |
| Guinea | 0.312 | 0.211 | 0.412 |
| Finland | 0.306 | -0.17 | 0.785 |
| Andorra | 0.283 | 0.015 | 0.552 |
| Dominica | 0.282 | 0.198 | 0.366 |
| Fiji | 0.209 | 0.063 | 0.356 |
| Argentina | 0.199 | -0.067 | 0.466 |
| Belgium | 0.175 | -0.093 | 0.442 |
| Gabon | 0.158 | 0.035 | 0.281 |
| Bosnia and Herzegovina | 0.158 | 0.045 | 0.27 |
| Portugal | 0.113 | -0.221 | 0.448 |
| Saint Lucia | 0.102 | -0.446 | 0.652 |
| Sweden | 0.101 | -0.16 | 0.364 |
| Mali | 0.101 | 0.036 | 0.167 |
| Germany | 0.083 | -0.242 | 0.409 |
| Palau | 0.039 | -0.039 | 0.118 |
| Slovenia | -0.005 | -0.339 | 0.33 |
| Greece | -0.026 | -0.151 | 0.099 |
| Cyprus | -0.068 | -0.355 | 0.22 |
| Turkmenistan | -0.095 | -1.151 | 0.971 |
| Slovakia | -0.098 | -0.198 | 0.003 |
| Kiribati | -0.107 | -0.306 | 0.093 |
| United Arab Emirates | -0.113 | -0.566 | 0.341 |
| Maldives | -0.128 | -0.369 | 0.113 |
| Israel | -0.137 | -0.566 | 0.293 |
| Luxembourg | -0.18 | -0.422 | 0.063 |
| Austria | -0.192 | -0.33 | -0.054 |
| Ethiopia | -0.24 | -0.467 | -0.012 |
| San Marino | -0.252 | -0.629 | 0.127 |
| Iceland | -0.286 | -0.925 | 0.357 |
| Czechia | -0.3 | -0.56 | -0.038 |
| Switzerland | -0.334 | -0.989 | 0.325 |
| Central African Republic | -0.339 | -0.392 | -0.287 |
| Malta | -0.348 | -0.756 | 0.063 |
| Rwanda | -0.373 | -0.624 | -0.12 |
| Mauritius | -0.394 | -1.34 | 0.562 |
| Cook Islands | -0.457 | -0.835 | -0.077 |
| Croatia | -0.578 | -0.928 | -0.226 |
| Liberia | -0.631 | -0.778 | -0.484 |
| Italy | -0.702 | -0.986 | -0.417 |
| Togo | -0.752 | -0.89 | -0.614 |
| Hungary | -0.779 | -1.038 | -0.518 |
| Kazakhstan | -0.782 | -1.548 | -0.011 |
| Sierra Leone | -0.81 | -1.005 | -0.614 |
| Senegal | -0.813 | -0.978 | -0.648 |
| Burundi | -0.871 | -1.037 | -0.705 |
| United States Virgin Islands | -0.922 | -1.153 | -0.69 |
| Chad | -0.926 | -1.109 | -0.744 |
| Guinea-Bissau | -0.991 | -1.163 | -0.819 |
| Mauritania | -1.077 | -1.294 | -0.861 |
| Tajikistan | -1.103 | -1.281 | -0.923 |
| Cameroon | -1.149 | -1.294 | -1.003 |
| Niger | -1.172 | -1.328 | -1.016 |
| Benin | -1.28 | -1.453 | -1.108 |
| Burkina Faso | -1.296 | -1.492 | -1.099 |
| Poland | -1.612 | -2.121 | -1.1 |

Table S7. Estimated annual percentage change (EAPC) in deaths rates of thyroid cancer for both sex by Country, 1990-2021.

| location | val | upper | lower |
| --- | --- | --- | --- |
| Iran (Islamic Republic of) | 3.202 | 2.759 | 3.648 |
| Cabo Verde | 2.707 | 2.421 | 2.993 |
| Saudi Arabia | 2.488 | 2.148 | 2.83 |
| Ecuador | 2.11 | 1.842 | 2.378 |
| Syrian Arab Republic | 2.087 | 1.909 | 2.265 |
| Viet Nam | 1.961 | 1.733 | 2.189 |
| Uzbekistan | 1.826 | 1.454 | 2.199 |
| Afghanistan | 1.817 | 1.743 | 1.892 |
| Armenia | 1.754 | 1.067 | 2.445 |
| Yemen | 1.74 | 1.59 | 1.891 |
| Georgia | 1.699 | 1.384 | 2.015 |
| Australia | 1.568 | 1.331 | 1.805 |
| Iraq | 1.551 | 1.142 | 1.962 |
| Oman | 1.503 | 1.373 | 1.632 |
| Algeria | 1.475 | 1.323 | 1.627 |
| Egypt | 1.289 | 1.196 | 1.382 |
| Sudan | 1.259 | 1.066 | 1.453 |
| India | 1.252 | 1.057 | 1.448 |
| Lesotho | 1.209 | 1.021 | 1.397 |
| Honduras | 1.201 | 0.724 | 1.68 |
| Libya | 1.196 | 0.91 | 1.482 |
| Cuba | 1.159 | 0.98 | 1.339 |
| Zambia | 1.137 | 1.04 | 1.234 |
| Republic of Korea | 1.108 | 0.971 | 1.246 |
| Nicaragua | 1.035 | 0.864 | 1.206 |
| Morocco | 1.009 | 0.783 | 1.237 |
| Belize | 0.989 | 0.758 | 1.22 |
| Peru | 0.988 | 0.923 | 1.053 |
| Latvia | 0.95 | 0.823 | 1.076 |
| Guam | 0.914 | 0.715 | 1.115 |
| Uganda | 0.914 | 0.731 | 1.096 |
| Kenya | 0.898 | 0.742 | 1.055 |
| Equatorial Guinea | 0.89 | 0.683 | 1.098 |
| China | 0.89 | 0.846 | 0.933 |
| Tunisia | 0.884 | 0.742 | 1.026 |
| Nepal | 0.862 | 0.576 | 1.148 |
| Belarus | 0.803 | 0.664 | 0.943 |
| Cambodia | 0.789 | 0.753 | 0.826 |
| Antigua and Barbuda | 0.747 | 0.728 | 0.766 |
| Lebanon | 0.734 | 0.586 | 0.881 |
| Panama | 0.699 | 0.634 | 0.764 |
| Namibia | 0.683 | 0.33 | 1.036 |
| Kuwait | 0.682 | 0.584 | 0.781 |
| Mozambique | 0.673 | 0.329 | 1.019 |
| Turkey | 0.663 | 0.505 | 0.822 |
| Zimbabwe | 0.656 | 0.595 | 0.717 |
| South Africa | 0.654 | 0.588 | 0.719 |
| Jamaica | 0.649 | 0.479 | 0.819 |
| Estonia | 0.648 | 0.51 | 0.787 |
| Timor-Leste | 0.626 | 0.46 | 0.792 |
| Nigeria | 0.6 | 0.377 | 0.823 |
| El Salvador | 0.593 | 0.365 | 0.822 |
| Mexico | 0.562 | 0.374 | 0.75 |
| Jordan | 0.517 | 0.391 | 0.643 |
| Bahamas | 0.507 | 0.382 | 0.633 |
| Taiwan (Province of China) | 0.502 | 0.325 | 0.68 |
| Barbados | 0.495 | 0.433 | 0.557 |
| Bhutan | 0.49 | 0.429 | 0.551 |
| Malawi | 0.487 | 0.337 | 0.636 |
| Paraguay | 0.48 | 0.24 | 0.722 |
| Venezuela (Bolivarian Republic of) | 0.478 | 0.289 | 0.668 |
| Philippines | 0.477 | 0.428 | 0.527 |
| Sao Tome and Principe | 0.43 | 0.256 | 0.604 |
| Dominican Republic | 0.426 | 0.178 | 0.675 |
| Bangladesh | 0.421 | 0.328 | 0.514 |
| Northern Mariana Islands | 0.417 | 0.32 | 0.515 |
| Qatar | 0.403 | 0.337 | 0.468 |
| Nauru | 0.381 | 0.255 | 0.507 |
| Guyana | 0.38 | 0.238 | 0.523 |
| Eswatini | 0.374 | 0.256 | 0.492 |
| Djibouti | 0.361 | 0.246 | 0.475 |
| Indonesia | 0.341 | 0.163 | 0.519 |
| Grenada | 0.33 | 0.21 | 0.45 |
| Solomon Islands | 0.278 | 0.181 | 0.374 |
| Democratic People's Republic of Korea | 0.276 | 0.223 | 0.329 |
| American Samoa | 0.256 | 0.092 | 0.421 |
| Bolivia (Plurinational State of) | 0.244 | 0.054 | 0.436 |
| South Sudan | 0.241 | 0.13 | 0.351 |
| Eritrea | 0.23 | 0.194 | 0.267 |
| Montenegro | 0.226 | 0.119 | 0.332 |
| Greenland | 0.225 | 0.183 | 0.268 |
| Pakistan | 0.216 | 0.067 | 0.365 |
| Netherlands | 0.192 | 0.002 | 0.382 |
| Malaysia | 0.187 | -0.134 | 0.508 |
| Saint Kitts and Nevis | 0.162 | -0.001 | 0.326 |
| France | 0.153 | 0.011 | 0.295 |
| United States of America | 0.131 | -0.065 | 0.328 |
| Comoros | 0.125 | 0.018 | 0.233 |
| Tonga | 0.122 | -0.041 | 0.285 |
| Gambia | 0.108 | -0.171 | 0.388 |
| Angola | 0.086 | -0.017 | 0.189 |
| Saint Vincent and the Grenadines | 0.066 | 0.013 | 0.118 |
| Lithuania | 0.06 | -0.319 | 0.44 |
| Russian Federation | 0.044 | -0.039 | 0.127 |
| Tokelau | 0.042 | -0.128 | 0.212 |
| Colombia | 0.033 | -0.058 | 0.123 |
| Norway | 0.027 | -0.07 | 0.123 |
| Serbia | 0.014 | -0.042 | 0.071 |
| Bermuda | -0.023 | -0.386 | 0.341 |
| Uruguay | -0.043 | -0.1 | 0.014 |
| Monaco | -0.057 | -0.22 | 0.106 |
| Vanuatu | -0.063 | -0.262 | 0.135 |
| Puerto Rico | -0.089 | -0.193 | 0.016 |
| Ghana | -0.11 | -0.26 | 0.039 |
| Palestine | -0.142 | -0.276 | -0.008 |
| Lao People's Democratic Republic | -0.158 | -0.309 | -0.006 |
| Albania | -0.186 | -0.295 | -0.078 |
| Tuvalu | -0.191 | -0.499 | 0.117 |
| Botswana | -0.196 | -0.343 | -0.048 |
| Republic of Moldova | -0.207 | -0.324 | -0.089 |
| Myanmar | -0.228 | -0.348 | -0.109 |
| Kyrgyzstan | -0.242 | -0.43 | -0.053 |
| Niue | -0.242 | -0.402 | -0.081 |
| United Republic of Tanzania | -0.251 | -0.395 | -0.107 |
| Marshall Islands | -0.26 | -0.438 | -0.081 |
| Thailand | -0.264 | -0.373 | -0.155 |
| Somalia | -0.266 | -0.319 | -0.214 |
| Azerbaijan | -0.275 | -0.49 | -0.058 |
| Singapore | -0.278 | -0.537 | -0.019 |
| Suriname | -0.279 | -0.458 | -0.1 |
| Guatemala | -0.284 | -0.38 | -0.187 |
| Micronesia (Federated States of) | -0.301 | -0.36 | -0.242 |
| Costa Rica | -0.328 | -1.035 | 0.385 |
| Japan | -0.332 | -0.369 | -0.296 |
| Cote d'Ivoire | -0.358 | -0.556 | -0.159 |
| Trinidad and Tobago | -0.367 | -0.67 | -0.063 |
| Ukraine | -0.373 | -0.454 | -0.293 |
| Mongolia | -0.374 | -0.543 | -0.204 |
| Chile | -0.389 | -0.434 | -0.345 |
| Samoa | -0.391 | -0.528 | -0.255 |
| Madagascar | -0.4 | -0.705 | -0.094 |
| Bulgaria | -0.403 | -0.809 | 0.004 |
| Canada | -0.417 | -0.482 | -0.352 |
| Denmark | -0.461 | -0.621 | -0.3 |
| United Kingdom | -0.509 | -0.722 | -0.295 |
| Bahrain | -0.529 | -0.711 | -0.346 |
| Sri Lanka | -0.532 | -0.707 | -0.357 |
| Ireland | -0.551 | -0.881 | -0.219 |
| New Zealand | -0.551 | -0.648 | -0.455 |
| Brunei Darussalam | -0.575 | -0.843 | -0.307 |
| Spain | -0.601 | -0.905 | -0.297 |
| Papua New Guinea | -0.63 | -0.761 | -0.499 |
| North Macedonia | -0.64 | -0.783 | -0.497 |
| Brazil | -0.642 | -0.894 | -0.39 |
| Romania | -0.687 | -0.782 | -0.593 |
| Democratic Republic of the Congo | -0.739 | -0.846 | -0.631 |
| Seychelles | -0.767 | -1.439 | -0.09 |
| Congo | -0.818 | -1.066 | -0.57 |
| Haiti | -0.828 | -1.064 | -0.592 |
| Guinea | -0.842 | -1.071 | -0.613 |
| Finland | -0.851 | -1.014 | -0.687 |
| Andorra | -0.854 | -0.966 | -0.741 |
| Dominica | -0.859 | -1.09 | -0.627 |
| Fiji | -0.89 | -0.975 | -0.804 |
| Argentina | -0.893 | -1.131 | -0.653 |
| Belgium | -0.913 | -1.287 | -0.538 |
| Gabon | -0.923 | -1.096 | -0.75 |
| Bosnia and Herzegovina | -0.924 | -1.184 | -0.664 |
| Portugal | -0.927 | -1.186 | -0.667 |
| Saint Lucia | -0.943 | -1.163 | -0.723 |
| Sweden | -0.963 | -1.156 | -0.77 |
| Mali | -0.993 | -1.111 | -0.874 |
| Germany | -1.016 | -1.106 | -0.926 |
| Palau | -1.023 | -1.368 | -0.677 |
| Slovenia | -1.038 | -1.266 | -0.811 |
| Greece | -1.06 | -1.321 | -0.797 |
| Cyprus | -1.061 | -1.188 | -0.934 |
| Turkmenistan | -1.064 | -1.333 | -0.794 |
| Slovakia | -1.072 | -1.272 | -0.873 |
| Kiribati | -1.088 | -1.2 | -0.975 |
| United Arab Emirates | -1.124 | -1.283 | -0.966 |
| Maldives | -1.127 | -1.298 | -0.956 |
| Israel | -1.128 | -1.475 | -0.781 |
| Luxembourg | -1.138 | -1.397 | -0.879 |
| Austria | -1.175 | -1.391 | -0.959 |
| Ethiopia | -1.201 | -1.436 | -0.967 |
| San Marino | -1.233 | -1.419 | -1.046 |
| Iceland | -1.238 | -1.571 | -0.904 |
| Czechia | -1.257 | -1.446 | -1.068 |
| Switzerland | -1.275 | -1.508 | -1.042 |
| Central African Republic | -1.291 | -1.595 | -0.986 |
| Malta | -1.297 | -1.559 | -1.035 |
| Rwanda | -1.33 | -1.478 | -1.183 |
| Mauritius | -1.345 | -1.663 | -1.027 |
| Cook Islands | -1.379 | -1.545 | -1.213 |
| Croatia | -1.393 | -1.577 | -1.209 |
| Liberia | -1.396 | -1.534 | -1.258 |
| Italy | -1.409 | -1.877 | -0.94 |
| Togo | -1.413 | -1.59 | -1.234 |
| Hungary | -1.45 | -1.81 | -1.088 |
| Kazakhstan | -1.464 | -1.755 | -1.172 |
| Sierra Leone | -1.465 | -1.662 | -1.267 |
| Senegal | -1.583 | -1.725 | -1.44 |
| Burundi | -1.629 | -2.157 | -1.098 |
| United States Virgin Islands | -1.72 | -1.901 | -1.538 |
| Chad | -1.725 | -1.86 | -1.589 |
| Guinea-Bissau | -1.733 | -1.812 | -1.653 |
| Mauritania | -1.797 | -2.122 | -1.472 |
| Tajikistan | -1.831 | -2.08 | -1.582 |
| Cameroon | -1.85 | -1.956 | -1.744 |
| Niger | -1.873 | -2.071 | -1.675 |
| Benin | -1.93 | -2.095 | -1.765 |
| Burkina Faso | -1.957 | -2.194 | -1.719 |
| Poland | -2.259 | -2.632 | -1.884 |

Table S8. Estimated annual percentage change (EAPC) in DALYs rates of thyroid cancer for both sex by Country, 1990-2021.

| location | val | upper | lower |
| --- | --- | --- | --- |
| Iran (Islamic Republic of) | 3.976 | 3.382 | 4.572 |
| Cabo Verde | 3.317 | 2.961 | 3.674 |
| Uzbekistan | 2.606 | 1.969 | 3.248 |
| Ecuador | 2.515 | 2.002 | 3.031 |
| Georgia | 2.504 | 1.312 | 3.71 |
| Lesotho | 2.333 | 1.917 | 2.75 |
| Afghanistan | 2.308 | 2.14 | 2.476 |
| Armenia | 1.873 | 1.601 | 2.146 |
| Zimbabwe | 1.833 | 1.357 | 2.311 |
| Kenya | 1.799 | 1.55 | 2.048 |
| Yemen | 1.796 | 1.602 | 1.991 |
| Syrian Arab Republic | 1.777 | 1.674 | 1.88 |
| Viet Nam | 1.755 | 1.543 | 1.968 |
| Zambia | 1.698 | 1.423 | 1.975 |
| Honduras | 1.607 | 1.401 | 1.813 |
| Guam | 1.567 | 0.815 | 2.324 |
| Uganda | 1.491 | 1.299 | 1.684 |
| Latvia | 1.475 | 1.021 | 1.931 |
| Mozambique | 1.39 | 1.212 | 1.568 |
| Cuba | 1.362 | 0.953 | 1.773 |
| Belize | 1.266 | 0.996 | 1.537 |
| India | 1.256 | 1.177 | 1.335 |
| Sudan | 1.225 | 1.049 | 1.402 |
| Saudi Arabia | 1.122 | 0.883 | 1.362 |
| Egypt | 1.121 | 0.935 | 1.308 |
| Northern Mariana Islands | 0.97 | 0.393 | 1.551 |
| Algeria | 0.963 | 0.81 | 1.116 |
| Libya | 0.957 | 0.734 | 1.18 |
| Philippines | 0.956 | 0.908 | 1.005 |
| Namibia | 0.925 | 0.78 | 1.07 |
| South Africa | 0.895 | 0.585 | 1.206 |
| Oman | 0.887 | 0.585 | 1.19 |
| Eswatini | 0.885 | 0.5 | 1.27 |
| Jamaica | 0.855 | 0.507 | 1.204 |
| Nicaragua | 0.847 | 0.666 | 1.028 |
| Morocco | 0.842 | 0.75 | 0.934 |
| Iraq | 0.842 | 0.612 | 1.072 |
| Nepal | 0.836 | 0.619 | 1.053 |
| Malawi | 0.794 | 0.601 | 0.987 |
| Panama | 0.724 | 0.579 | 0.87 |
| Guyana | 0.709 | 0.52 | 0.899 |
| Lithuania | 0.706 | 0.215 | 1.199 |
| Timor-Leste | 0.702 | 0.578 | 0.826 |
| Solomon Islands | 0.693 | 0.611 | 0.776 |
| Nigeria | 0.691 | 0.538 | 0.844 |
| Djibouti | 0.691 | 0.653 | 0.728 |
| Nauru | 0.685 | 0.501 | 0.868 |
| South Sudan | 0.683 | 0.48 | 0.887 |
| Antigua and Barbuda | 0.662 | 0.154 | 1.172 |
| Sao Tome and Principe | 0.622 | 0.476 | 0.769 |
| Bahamas | 0.622 | 0.278 | 0.967 |
| Dominican Republic | 0.622 | 0.512 | 0.731 |
| American Samoa | 0.607 | 0.356 | 0.859 |
| Australia | 0.598 | 0.35 | 0.847 |
| Tonga | 0.589 | 0.392 | 0.787 |
| Eritrea | 0.588 | 0.517 | 0.66 |
| Paraguay | 0.569 | 0.492 | 0.646 |
| Cambodia | 0.566 | 0.451 | 0.681 |
| Belarus | 0.552 | -0.019 | 1.126 |
| Vanuatu | 0.543 | 0.456 | 0.63 |
| Somalia | 0.541 | 0.505 | 0.578 |
| Pakistan | 0.48 | 0.263 | 0.698 |
| Mexico | 0.425 | 0.295 | 0.554 |
| Barbados | 0.425 | 0.221 | 0.629 |
| Kuwait | 0.41 | -0.508 | 1.337 |
| Gambia | 0.403 | 0.216 | 0.59 |
| Comoros | 0.358 | 0.291 | 0.426 |
| Tunisia | 0.347 | 0.234 | 0.459 |
| Ghana | 0.33 | 0.195 | 0.465 |
| Saint Vincent and the Grenadines | 0.292 | -0.216 | 0.802 |
| Lebanon | 0.286 | 0.065 | 0.508 |
| Indonesia | 0.281 | 0.111 | 0.452 |
| Grenada | 0.277 | -0.244 | 0.8 |
| Montenegro | 0.263 | 0.109 | 0.417 |
| United Republic of Tanzania | 0.252 | 0.166 | 0.338 |
| Bhutan | 0.244 | 0.181 | 0.308 |
| Venezuela (Bolivarian Republic of) | 0.239 | 0.069 | 0.41 |
| Marshall Islands | 0.208 | 0.084 | 0.331 |
| Equatorial Guinea | 0.193 | 0.018 | 0.368 |
| Peru | 0.19 | -0.014 | 0.394 |
| Democratic People's Republic of Korea | 0.183 | 0.023 | 0.343 |
| Estonia | 0.168 | -0.222 | 0.559 |
| Angola | 0.155 | 0.083 | 0.228 |
| United States of America | 0.153 | 0.045 | 0.261 |
| Papua New Guinea | 0.106 | 0.044 | 0.168 |
| Botswana | 0.07 | -0.21 | 0.351 |
| Bolivia (Plurinational State of) | 0.058 | 0.014 | 0.102 |
| Madagascar | 0.021 | -0.114 | 0.155 |
| Niue | 0.014 | -0.076 | 0.105 |
| Suriname | 0.008 | -0.178 | 0.194 |
| Tokelau | -0.025 | -0.207 | 0.158 |
| Bangladesh | -0.028 | -0.122 | 0.065 |
| El Salvador | -0.042 | -0.246 | 0.162 |
| Japan | -0.067 | -0.131 | -0.003 |
| Tuvalu | -0.088 | -0.197 | 0.02 |
| Ukraine | -0.099 | -0.449 | 0.251 |
| Guinea | -0.104 | -0.204 | -0.004 |
| Uruguay | -0.148 | -0.225 | -0.07 |
| Dominica | -0.165 | -0.244 | -0.087 |
| Cote d'Ivoire | -0.196 | -0.319 | -0.074 |
| Republic of Moldova | -0.23 | -0.453 | -0.006 |
| Monaco | -0.241 | -0.271 | -0.212 |
| Micronesia (Federated States of) | -0.247 | -0.33 | -0.163 |
| Democratic Republic of the Congo | -0.252 | -0.366 | -0.138 |
| Samoa | -0.271 | -0.353 | -0.188 |
| Saint Kitts and Nevis | -0.275 | -0.556 | 0.008 |
| Haiti | -0.279 | -0.353 | -0.204 |
| Lao People's Democratic Republic | -0.285 | -0.338 | -0.232 |
| Fiji | -0.324 | -0.463 | -0.185 |
| Taiwan (Province of China) | -0.347 | -0.557 | -0.136 |
| Russian Federation | -0.366 | -0.806 | 0.077 |
| Malaysia | -0.372 | -0.515 | -0.229 |
| Jordan | -0.393 | -0.768 | -0.017 |
| Myanmar | -0.425 | -0.58 | -0.27 |
| Greenland | -0.429 | -0.918 | 0.062 |
| Azerbaijan | -0.431 | -0.576 | -0.287 |
| Netherlands | -0.498 | -0.614 | -0.383 |
| Costa Rica | -0.508 | -0.752 | -0.263 |
| Republic of Korea | -0.518 | -1.21 | 0.18 |
| Mali | -0.528 | -0.603 | -0.453 |
| China | -0.532 | -0.623 | -0.441 |
| Kiribati | -0.545 | -0.727 | -0.363 |
| Guatemala | -0.557 | -0.998 | -0.113 |
| Serbia | -0.568 | -0.788 | -0.348 |
| Trinidad and Tobago | -0.57 | -0.849 | -0.291 |
| Kyrgyzstan | -0.581 | -1.375 | 0.219 |
| Palestine | -0.589 | -0.78 | -0.397 |
| Central African Republic | -0.613 | -0.669 | -0.557 |
| Congo | -0.618 | -0.721 | -0.514 |
| Turkey | -0.635 | -0.809 | -0.462 |
| Puerto Rico | -0.653 | -0.982 | -0.323 |
| Canada | -0.664 | -0.836 | -0.492 |
| Norway | -0.665 | -0.922 | -0.408 |
| United Kingdom | -0.675 | -0.823 | -0.527 |
| Palau | -0.694 | -0.749 | -0.64 |
| Brazil | -0.706 | -0.819 | -0.592 |
| Greece | -0.726 | -0.806 | -0.646 |
| Gabon | -0.764 | -0.905 | -0.623 |
| Bermuda | -0.789 | -1.298 | -0.278 |
| Bulgaria | -0.799 | -1.092 | -0.506 |
| Brunei Darussalam | -0.803 | -0.903 | -0.702 |
| Seychelles | -0.811 | -1.172 | -0.448 |
| Colombia | -0.817 | -1.21 | -0.423 |
| Thailand | -0.862 | -1.098 | -0.626 |
| Qatar | -0.884 | -1.125 | -0.643 |
| New Zealand | -0.906 | -1.296 | -0.515 |
| Argentina | -0.915 | -1.219 | -0.611 |
| Sweden | -0.925 | -1.207 | -0.643 |
| Denmark | -1.004 | -1.307 | -0.699 |
| Spain | -1.02 | -1.133 | -0.906 |
| Saint Lucia | -1.061 | -1.673 | -0.444 |
| Mongolia | -1.061 | -1.444 | -0.676 |
| Andorra | -1.072 | -1.258 | -0.885 |
| Albania | -1.137 | -1.403 | -0.871 |
| San Marino | -1.173 | -1.52 | -0.825 |
| North Macedonia | -1.215 | -1.34 | -1.09 |
| Belgium | -1.229 | -1.481 | -0.977 |
| Turkmenistan | -1.285 | -2.26 | -0.3 |
| Chad | -1.318 | -1.551 | -1.086 |
| Ethiopia | -1.333 | -1.522 | -1.145 |
| Burundi | -1.339 | -1.514 | -1.163 |
| Romania | -1.342 | -1.654 | -1.03 |
| United Arab Emirates | -1.398 | -1.868 | -0.927 |
| Mauritius | -1.402 | -2.266 | -0.531 |
| Chile | -1.402 | -1.632 | -1.172 |
| France | -1.404 | -1.74 | -1.067 |
| Sierra Leone | -1.415 | -1.63 | -1.199 |
| Sri Lanka | -1.425 | -1.695 | -1.155 |
| Togo | -1.428 | -1.6 | -1.256 |
| Rwanda | -1.462 | -1.725 | -1.198 |
| Singapore | -1.484 | -1.713 | -1.254 |
| Bosnia and Herzegovina | -1.485 | -1.686 | -1.283 |
| Finland | -1.511 | -1.836 | -1.184 |
| Senegal | -1.514 | -1.668 | -1.361 |
| Portugal | -1.547 | -1.72 | -1.373 |
| Iceland | -1.597 | -2.124 | -1.068 |
| Guinea-Bissau | -1.621 | -1.818 | -1.424 |
| Slovakia | -1.63 | -1.769 | -1.491 |
| Austria | -1.647 | -1.788 | -1.506 |
| Switzerland | -1.652 | -2.105 | -1.198 |
| Liberia | -1.677 | -1.841 | -1.513 |
| Italy | -1.696 | -1.79 | -1.602 |
| Ireland | -1.698 | -2.092 | -1.303 |
| United States Virgin Islands | -1.711 | -1.946 | -1.475 |
| Tajikistan | -1.734 | -2.02 | -1.446 |
| Bahrain | -1.736 | -1.966 | -1.505 |
| Germany | -1.777 | -1.996 | -1.557 |
| Cameroon | -1.817 | -2.004 | -1.629 |
| Cook Islands | -1.818 | -2.212 | -1.423 |
| Burkina Faso | -1.848 | -2.06 | -1.636 |
| Israel | -1.861 | -2.168 | -1.553 |
| Niger | -1.868 | -2.058 | -1.678 |
| Slovenia | -1.919 | -2.132 | -1.705 |
| Benin | -1.935 | -2.128 | -1.742 |
| Croatia | -1.955 | -2.254 | -1.654 |
| Luxembourg | -1.963 | -2.078 | -1.847 |
| Malta | -1.982 | -2.276 | -1.687 |
| Czechia | -2.063 | -2.195 | -1.931 |
| Mauritania | -2.258 | -2.447 | -2.068 |
| Hungary | -2.324 | -2.634 | -2.014 |
| Kazakhstan | -2.425 | -2.986 | -1.861 |
| Maldives | -2.427 | -2.61 | -2.243 |
| Cyprus | -2.447 | -2.606 | -2.289 |
| Poland | -3.297 | -3.888 | -2.703 |

Table S9. Global age and sex structure of thyroid cancer incidence, deaths and DALYs, 2021.

| sex | age | metric | incidence | | | deaths | | | DALYs | | |
| --- | --- | --- | --- | --- | --- | --- | --- | --- | --- | --- | --- |
|  |  |  | val | upper | lower | val | upper | lower | val | upper | lower |
| Male | 55-59 years | Number | 11440.9637804548 | 13132.13806537958 | 9759.37018101268 | 1791.646325216141 | 2061.698257774985 | 1453.721750849365 | 66017.00700782858 | 75564.33479987933 | 53302.70322463838 |
| Female | 55-59 years | Number | 19600.76603528122 | 22578.21877029521 | 17266.17197555629 | 2082.952757826295 | 2374.259086734096 | 1763.31109013966 | 79538.0040549936 | 92534.64428719658 | 67627.16746441492 |
| Male | 55-59 years | Rate | 5.875462486 | 6.743958467040001 | 5.011886628 | 0.9200930073023914 | 1.058777127744475 | 0.7465531554384937 | 33.90277738191902 | 38.80577046501534 | 27.37339608663442 |
| Female | 55-59 years | Rate | 9.751453935 | 11.23274773 | 8.589984715 | 1.036276736796297 | 1.181202717855856 | 0.8772538194066108 | 39.5704525624547 | 46.03632937052868 | 33.64476710068764 |
| Male | 60-64 years | Number | 7914.968997604027 | 8551.352367722187 | 7163.894635214687 | 1806.604988007405 | 1992.618077561072 | 1544.259466000453 | 56330.54791477493 | 62144.72621455597 | 48497.35345290645 |
| Female | 60-64 years | Number | 16904.67961625687 | 19380.73409619244 | 14772.90651275888 | 2675.789353790012 | 3054.902062268193 | 2227.211829568771 | 85646.76770684123 | 98536.19702229043 | 70633.75402484345 |
| Male | 60-64 years | Rate | 5.088803983 | 5.497956593 | 4.605912615 | 1.16152806925644 | 1.28112223964783 | 0.9928571701514272 | 36.21683378156246 | 39.95496765127237 | 31.18060544177498 |
| Female | 60-64 years | Rate | 10.27571804 | 11.7808183 | 8.979893462 | 1.62651156717576 | 1.856959903749667 | 1.353838185436092 | 52.0614442871142 | 59.89644290020676 | 42.93559872032846 |
| Male | 65-69 years | Number | 7281.465207113903 | 7892.208749285091 | 6630.650501534072 | 2142.253284314159 | 2348.606180453574 | 1857.47688698033 | 55764.88565554381 | 61211.80676487346 | 48403.38512838179 |
| Female | 65-69 years | Number | 15244.02991940682 | 17609.84345180391 | 13308.89672827658 | 3368.290524161976 | 3917.934416988176 | 2837.428816835929 | 89174.87067692749 | 103397.8099578637 | 75120.97915037573 |
| Male | 65-69 years | Rate | 5.523239357870001 | 5.986509136 | 5.029574238 | 1.624972078819917 | 1.781497778682492 | 1.408959482287686 | 42.29956505719843 | 46.43124023445428 | 36.71561618315764 |
| Female | 65-69 years | Rate | 10.58547603 | 12.22830031 | 9.241716794 | 2.338945724821432 | 2.720619224799554 | 1.97031460113575 | 61.92315687590425 | 71.79958611705979 | 52.16411463553489 |
| Male | 70-74 years | Number | 5855.876354350109 | 6399.277553044709 | 5281.932366053621 | 2223.823690727589 | 2460.317870971824 | 1914.455912465924 | 47533.78142246254 | 52664.94831343314 | 40825.75437366355 |
| Female | 70-74 years | Number | 11899.10327976988 | 13538.59701230451 | 10418.64287530946 | 3679.716033320429 | 4321.545154422717 | 3147.421686406964 | 79174.3979436363 | 92646.22110987772 | 68056.4464490744 |
| Male | 70-74 years | Rate | 6.075101728 | 6.638846138 | 5.479671103 | 2.30707657192313 | 2.55242434158338 | 1.986127048671186 | 49.31329491269081 | 54.63655636104461 | 42.35414068088782 |
| Female | 70-74 years | Rate | 10.87194297 | 12.36991151 | 9.519279608 | 3.36207375732265 | 3.948498586087571 | 2.875728387537666 | 72.33986622003557 | 84.64876797233765 | 62.18164405925573 |
| Male | 75-79 years | Number | 4928.475079537791 | 5504.944956832702 | 4205.384096739785 | 2577.470684350884 | 2937.455805877684 | 2110.774917677469 | 43403.28845915473 | 49225.95739217251 | 35509.86064224452 |
| Female | 75-79 years | Number | 7467.601697561277 | 8458.72881910346 | 6435.908658182425 | 3293.497299593252 | 3806.483415179211 | 2803.234864121786 | 55708.07489757477 | 64488.47848599231 | 47683.90193552188 |
| Male | 75-79 years | Rate | 8.243427333 | 9.207637857 | 7.033976564280001 | 4.311108800478573 | 4.913224291016681 | 3.5305077876067 | 72.59686792251364 | 82.33593476498592 | 59.39422459701094 |
| Female | 75-79 years | Rate | 10.35761699 | 11.7323174 | 8.926651361 | 4.568104321025715 | 5.279619733995334 | 3.888106814973294 | 77.26749849986636 | 89.44598110483264 | 66.13791318484621 |
| Male | 80-84 years | Number | 2608.540206679306 | 2882.202679529226 | 2223.023494483018 | 1987.309698294052 | 2200.999981878374 | 1642.828645105062 | 25752.87049062313 | 28530.60858491412 | 21295.56963587228 |
| Female | 80-84 years | Number | 4568.427516780241 | 5170.850192543429 | 3759.142564715402 | 3095.575837573343 | 3590.752382043569 | 2577.877222197151 | 40190.07578494089 | 46420.55456969829 | 33551.44653773589 |
| Male | 80-84 years | Rate | 7.117074138830001 | 7.863727805 | 6.065240237 | 5.422124766704562 | 6.005151851019241 | 4.482251503991724 | 70.2634707719731 | 77.84218008406607 | 58.10228553851679 |
| Female | 80-84 years | Rate | 8.969802383 | 10.15261908 | 7.380825418 | 6.07795645720066 | 7.050202538006232 | 5.061489794030103 | 78.91053020492436 | 91.14365927307004 | 65.87602495706031 |
| Male | 85-89 years | Number | 1425.953428672257 | 1573.806663304267 | 1210.294925252249 | 1253.376201385561 | 1388.206383199996 | 1038.77170990278 | 12846.88760256123 | 14245.5776096483 | 10670.54986284349 |
| Female | 85-89 years | Number | 2685.058067136576 | 3072.777686578506 | 2038.379240190161 | 2126.849727774394 | 2420.775002395116 | 1654.789123225761 | 21838.28043223976 | 24884.85010238684 | 16997.14219307694 |
| Male | 85-89 years | Rate | 8.265122423 | 9.122110498 | 7.015120917300001 | 7.264828947174404 | 8.046332702164706 | 6.020936714184611 | 74.46323045946582 | 82.57032842444774 | 61.84872462087446 |
| Female | 85-89 years | Rate | 9.431471635 | 10.79336642 | 7.15996284 | 7.470722188259155 | 8.503157175143254 | 5.812573243107829 | 76.70862875172783 | 87.40993751634002 | 59.70376075967195 |
| Male | 90-94 years | Number | 394.1311192590081 | 437.3765054763717 | 329.2089067835354 | 520.8487343839626 | 578.4921983500899 | 434.004702734489 | 4585.098596868189 | 5083.920687486053 | 3820.324421949274 |
| Female | 90-94 years | Number | 935.1493268040248 | 1074.812630379479 | 704.2627602115818 | 1204.023913237746 | 1379.257119819383 | 908.5098285721964 | 10584.01085219324 | 12137.08048888722 | 8031.011443576413 |
| Male | 90-94 years | Rate | 6.762137523 | 7.504101896 | 5.648262197 | 8.936241262296457 | 9.92523454804393 | 7.446251620816204 | 78.66688458308886 | 87.2252130467197 | 65.54559602637897 |
| Female | 90-94 years | Rate | 7.753578078 | 8.911564613 | 5.839234593 | 9.98289059464988 | 11.43579689544753 | 7.532702733794855 | 87.75492017089924 | 100.6318440413699 | 66.58730588665111 |
| Male | 95+ years | Number | 82.67293257778553 | 93.75659106633825 | 63.04571762673379 | 116.9576515410382 | 132.1961599429567 | 89.27940489898204 | 961.2169494358632 | 1090.863838537571 | 733.9802963664268 |
| Female | 95+ years | Number | 355.4436154100725 | 417.967233781856 | 244.5054241347332 | 495.0568834239163 | 581.7835440934244 | 339.1562335303707 | 4037.875576106198 | 4735.858257180325 | 2767.775445779014 |
| Male | 95+ years | Rate | 5.467635095 | 6.200660987 | 4.169574824 | 7.735080155669697 | 8.742890097034051 | 5.904558992464068 | 63.57078868214004 | 72.14508088039828 | 48.54232579289113 |
| Female | 95+ years | Rate | 9.025381826 | 10.61297408 | 6.208452524299999 | 12.57042525106229 | 14.77257825958189 | 8.611814570760494 | 102.5292543181737 | 120.2523471843833 | 70.27902351798706 |
